# The origin and development of the Miocene northwest African savanna

**DOI:** 10.1073/pnas.2527340123

**Published:** 2026-08-10

**Authors:** Anna K. Schartman, Pratigya J. Polissar, Caroline A. E. Strömberg

**Affiliations:** ^a^ Ocean Sciences Department, University of California Santa Cruz, Santa Cruz, CA 95064; ^b^ Department of Biology, Burke Museum of Natural History and Culture, University of Washington, Seattle, WA 98195

**Keywords:** plant wax, C_4_ plants, Miocene, savanna ecosystem, International Ocean Discovery Program (IODP)

## Abstract

Like many of Earth’s subtropical open ecosystems, the African savanna originated in the Miocene Epoch (23.0 to 5.3 Ma), one of the best historical analogs for future climate states. The first C_4_ grass, C_3_ tree, and shrub savanna developed in the subtropics after ~10 Ma in northwest Africa, but the terrestrial sedimentary record of the region is not well preserved, making it difficult to determine when, how, and why the African landscape opened. Here, we leverage plant-wax biomarkers extracted from the marine sedimentary archive to document the origination and history of open ecosystems through Miocene northwest Africa. We show that a C_3_ grass-rich savanna with no modern analog replaced closed woodlands and forests during the middle Miocene (~15 to 14 Ma), a period of global cooling and aridification. This nonanalog ecosystem transitioned into the C_4_ savanna in stages in the late Miocene (most rapidly between 7.4 to 6.4 Ma) as global temperatures cooled and aridity increased, roughly coincident with several C_4_ expansions in other subtropical regions. Our new vegetation history, put in the context of other records, links initial ecosystem opening and C_4_ expansion to periods of global cooling and drying. It is consistent with a nonlinear system of feedbacks between climate, consumers (herbivory and fire), and plant-trait interactions that allows small changes in one parameter to have dramatic effects on regional vegetation structure and composition. The geologic record indicates that the response of subtropical open ecosystems to future climate perturbations will depend on the nature and strength of these feedbacks.

## Significance

In modern Africa, savannas cover ~50% of the continent, provide critical ecosystem services, and affect global climate. Here, we uncover the history of savanna development in northwest Africa. We show that C_3_, grass-rich ecosystems with no modern counterpart initially expanded in the region ~15 Mya, before staggered replacement in the late Miocene (~10 to 5 Ma) by savannas composed of C_4_ grasses. The original landscape opening and subsequent transformation into the C_4_ savanna occurred during periods of global cooling and aridification. Our results indicate that the system of climate, herbivore, fire, and plant-trait interactions that control modern open ecosystems, in which small changes to one parameter lead to dramatic ecosystem reorganization, was operating through geologic time.

Open, grass-rich ecosystems cover 40% of terrestrial landscapes and are intimately entwined with Earth’s climate and human evolution. Defined by a discontinuous canopy and an understory of grasses, these ecosystems provide habitat for a diverse array of animal and plant species. Although long-standing biases regarding grassland- and savanna ecosystems as recent, man-made, degraded, and early successional have led to poor policy decisions regarding their protection (1), a rapidly growing body of research in the last 20 y highlights their economic, biogeographic, and cultural significance (2).

A central question in the study of open ecosystems is their origin(s) and geologic history, including which drivers caused them to expand to global prominence and persist to the present (1). There is now widespread recognition that grassland and savanna ecosystems originated millions of years ago, yet the factors determining their rise remain unclear, as does the history of their assembly and spread. This is especially true for northwest (NW) and east Africa, likely the first regions on Earth to host extensive grass-rich, C_4_ ecosystems akin to modern savannas (e.g., refs. 3–13).

**Fig. 1.**
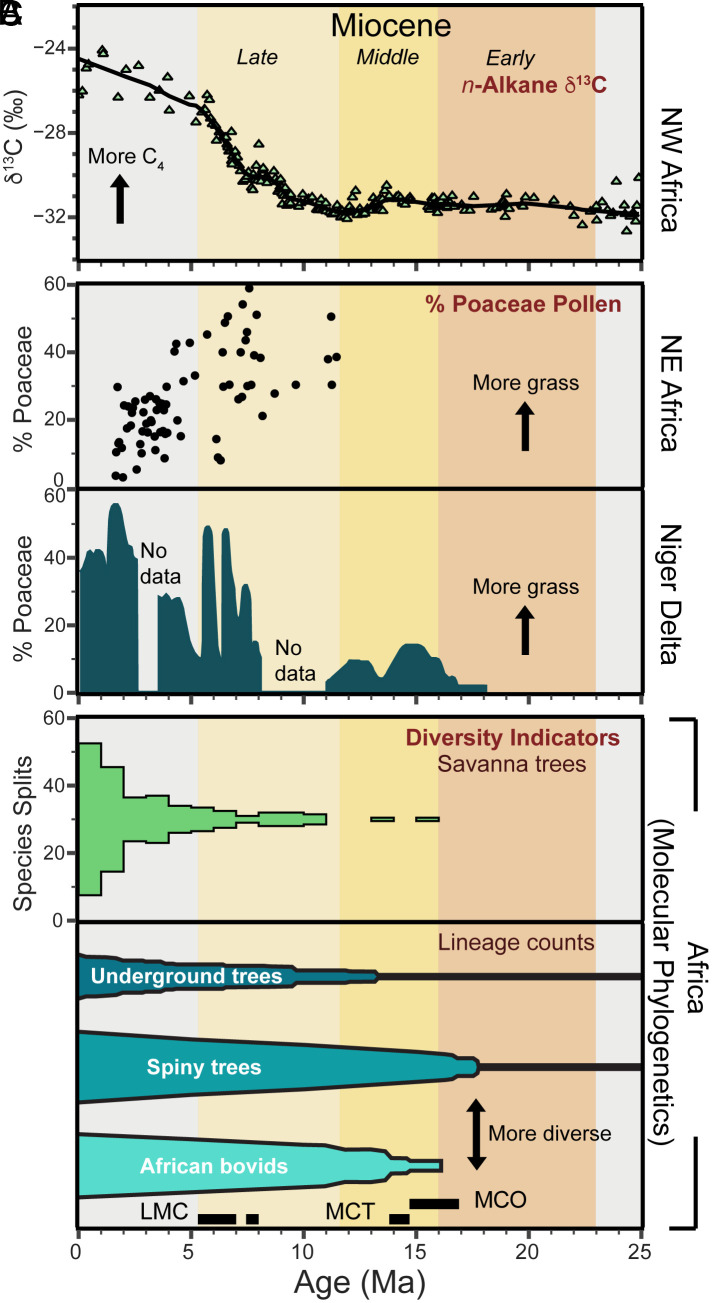
Neogene vegetation history of Africa suggestive of landscape opening in the middle and late Miocene. (*A*) Compilation of *n*-alkane C_31_ δ^13^C from marine cores off the NW African coast showing expansion of C_4_ open ecosystems occurring primarily after 11 Ma (8, 38, 39; this study) (*B*) High abundances of grass pollen (% Poaceae) occur in Gulf of Aden and Niger Delta sediments before increases in C_4_ (18, 27). (*C*) Savanna tree species evolutionary splits (35) and the diversity of tree spinescence, bovids, and underground trees (22, 32, 34) increase in the middle to late Miocene, suggesting that herbivore pressure led to opening of vegetation before fire became an important consumer (line width ∝ log lineage counts). MCT = Miocene Climate Transition. MCO = Miocene Climatic Optimum. LMC = Late Miocene Cooling as identified by Herbert et al. (40) and a possible earlier period (8.0 to 7.0 Ma) as depicted in Steinthorsdottir et al. (41). Line in (*A*) is a locally weighted scatterplot smoothing curve fit to new data and that of Polissar et al. (8), O’Mara et al. (39), and O’Mara et al. (38). Orbitally sampled datasets were averaged before fit. Lineage counts and % Poaceae digitized from figure 5 of Charles-Dominique et al. (34) and figure 3 of Morley and Richards (27), respectively. NW Africa = northwest Africa. NE Africa = northeast Africa.

The origin of Africa’s grass-rich biomes can be traced to the Neogene Period (23.03 to 2.58 Ma). Early–middle Miocene fossil flora and fauna records in Eastern Africa are generally interpreted as representing moist evergreen to dry deciduous forests and woodlands (12–17). The earliest widespread occurrence of modern-like C_4_ grass–C_3_ tree savanna began in NW and east Africa at ~10 Ma based on terrestrial plant-wax biomarker δ^13^C (8, 18, 19). This timing is congruent with increases in grazing and C_4_ diet composition in fossil faunas from east Africa (20, 21) as well as the late Miocene diversification of certain ecological strategies associated with open ecosystems (e.g., underground trees, diapausing mating strategies in butterflies) (22, 23). On the other hand, there is evidence that the first widespread, open ecosystems in Africa were not dominated by C_4_ grasses but constituted C_3_ grass-rich savannas, an ecosystem type that does not exist in the subtropics today (Fig. 1). For example, floral assemblages from east African localities demonstrate that grassland- to open woodland vegetation was present, at least locally, since the early Miocene (13, 24–26). Regional pollen records also document medium to high abundances of grass pollen from tropical west, east, and southwest Africa as well as western South Africa in the middle and early late Miocene, always prior to increases in plant-wax δ^13^C when both measurements are present (18, 27–31). Molecular phylogenetic studies show middle-Miocene increases in the diversification of some plant and animal species and functional traits associated with open ecosystems (e.g., the thorny trees and shrubs that grow in savannas, and the browsers that consume them) (32–37). Taken together, the fossil record from Africa paints an equivocal picture of the development of open ecosystems through the Miocene, with no clear answer to whether savannas existed prior to C_4_ vegetation expansion at 10 Ma. This problem is particularly acute for NW Africa, where the terrestrial fossil record is largely nonexistent.

Documenting the history of open ecosystems is fundamental for revealing the complex system of controls that governs their current distributions and response to future climate changes (9, 42–45). Among these, climate-focused controls include atmospheric CO2 concentrations and precipitation amounts and seasonality, whereas consumer controls (disturbance) encompass herbivory and fire regimes (21, 34, 44, 46–55). This system of controls is complex and involves both direct effects and feedbacks. For example, the direct effects of lower CO_2_ favor plants with the C_4_ photosystem, a carbon-concentrating mechanism that evolved independently 18 to 24 times in grasses (52). C_4_ plants are favored under low CO_2_, but also under high light and growing season temperatures (53, 54). However, atmospheric CO_2_ concentrations have an indirect effect through global temperature change, which in turn can influence precipitation dynamics (55). Increased seasonality of precipitation selects for open-ecosystem grasses, which accumulate biomass, die back, and cure quickly, therefore promoting recurrent fires (45, 56). Frequent fires and herbivory are important feedback mechanism for maintaining open ecosystems in regions where forests and woodlands would also be competitive (57, 58). The effects of these climate- and consumer controls differ geographically (42), resulting in part from the unique evolutionary trajectories of regional biotic communities. For these reasons, comparing ecosystem histories across regions is crucial to determine how climate and disturbance interacted with plant evolution to cause the global rise of grasslands and savannas in the Neogene.

**Fig. 2.**
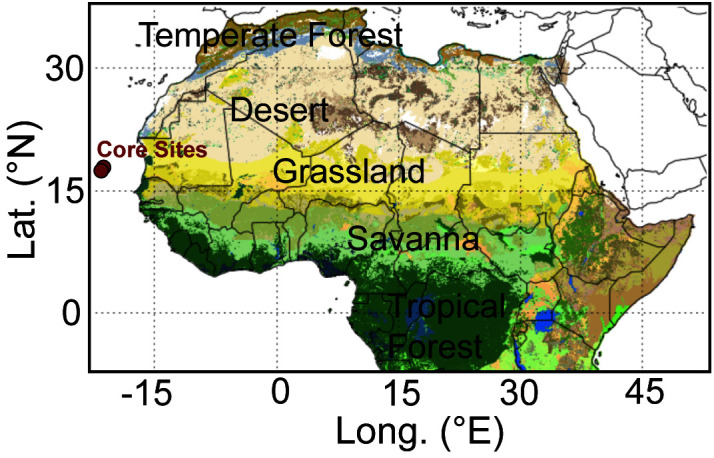
Map of study area showing the major ecosystem divisions in the modern day summarized from Sayre et al. (64). The dark red circles mark the locations of the core sites (ODP 659 and DSDP 368) investigated in this study.

Here, we investigate the origin and development of NW African open ecosystems to understand the controls and feedbacks that determine their distribution. We provide a regional record of Miocene NW African vegetation structure and composition using the distributions and carbon isotope composition of plant-wax biomarkers preserved in offshore sediments. We leverage the continuous Oligocene to present marine record available for NW Africa, a crucial advantage for this region since there are very few terrestrial sites that provide either floral or faunal fossil assemblages (12). Previous work on NW African marine cores showed that C_4_ vegetation spread in NW Africa started at ~10 Ma (8, 59) and that C_4_ grasslands and savannas have been widespread in this region since at least the Plio-Pleistocene, although the relative amounts of C_3_ and C_4_ vegetation and their northern and southern boundaries have shifted (38, 39, 60, 61; Fig. 2). These studies highlight the importance of NW Africa as a site of early C_4_ savanna development but lack the sample coverage and resolution to uncover the timing and drivers of open ecosystem origination. We use two marine drill core sites with near continuous deposition through the Miocene Epoch (ODP 659 and DSDP 368; 62, 63; Fig. 2) to provide a record of regional vegetation change at a 100 to 250 ky resolution. Plant-wax biomarkers are well preserved and abundant in these sediments (unlike pollen grains, see *SI Appendix*, section S1), and can provide information on plant functional type through compound distributions and C_3_/C_4_ photosystem through molecular carbon isotope ratios. Together these parameters capture vegetation structure and document the development of open ecosystems for this region. They provide evidence for a nonanalog C_3_ open ecosystem arising in the middle Miocene of NW Africa, and trace its subsequent, stepwise demise and replacement in the late Miocene by a C_4_ grass-rich savanna.

## Results

Miocene NW African sediment samples contain *n*-alkanes with long-chain (*n*-C_25_ to *n*-C_35_) distributions characteristic of terrestrial plant waxes. They do not exhibit overprinting of the primary plant-wax signal by thermal maturation, petroleum sources, or microbial alteration, as evidenced by their high carbon preference index (CPI, an indicator of biosynthetic origin; 65). They all display CPI_(24–36)_ values above one (mean = 4.9, 1σ = 0.9), suggesting that, to a first order, plant-wax sources are the main contributors to the long-chain *n*-alkanes of most samples (*SI Appendix*, Fig. S5 and section S6; 65). We identify all samples with a CPI_(24–36)_ value above 3.0 on subsequent plots as representative of terrestrial vascular plants (158 samples, ~96% of total) and therefore reliable indicators of past vegetation (see *SI Appendix*, section S6 for discussion on *n*-alkane source and screening).

### *n*-Alkane Distributions.

Plant-wax *n*-alkane distributions reflect vegetation structure because plant growth forms tend to produce different *n*-alkane homologs. For African plants, *n*-alkane distributions are distinct between woody plants (trees and shrubs with cellulose- and lignin-rich cell walls) and herbaceous vegetation (plants that lack lignification like grasses and forbs). Tree and shrub *n*-alkane compositions favor the *n*-C_29_ *n*-alkane with lower amounts of C_31_, C_33_, and C_35_, while grasses, and to a degree forbs, show the opposite pattern, favoring C_31_, C_33_, and C_35_ (66–78). Importantly, preference for C_33_ and C_35_ compared to C_29_ chain lengths are shared across African grasses from the PACMAD clade (Panicoideae-Arundinoideae-Chloridoideae-Micrairoideae-Aristidoideae-Danthonioideae), independent of photosystem (73). Grasses of Miocene northwest Africa are likely to be from the warm-adapted PACMAD clade, rather than grasses from the BOP (Bambusoideae-Oryzoideae-Pooideae) sister clade. Modern savannas in Africa largely contain PACMADs, and phytolith analysis demonstrates that the same was true also of east African ecosystems in the early Miocene (24, 79).This preference can be assessed with three metrics (%C_31_, %C_33_, and %C_35_) that quantify the relative abundance of the longer chain lengths with respect to C_29_:

[1] $$\%C_{n}=\frac{C_{n}}{\left(C_{n}+C_{29}\right)},n=31,33,or\;35.$$

We quantitatively investigated %C_31_, %C_33_, and %C_35_ differences using a dataset of 967 modern African plant distributions (*SI Appendix*, section S7). Median distribution values for woody plants are always lower than those of grasses, albeit with a high degree of variation across taxa (*SI Appendix*, Fig. S8). Two-sample Kolmogorov–Smirnov tests show that for %C_33_, grass distributions can be distinguished from both tree and shrub distributions (*P* always < 2.35 × 10^−24^), while tree and shrub distributions are not statistically distinguishable (*P* = 0.435, *SI Appendix*, Fig. S9 and Table S1). These relationships translate into modern sediments: *n*-alkanes of soils, lake sediments, and marine sediments sourced from open, grass-rich subtropical vegetation tend to have higher values of %C_31_, %C_33_, and %C_35_ compared with those of forests and woodlands (71, 80–84; *SI Appendix*, Fig. S10 and section S7). These relationships persist in older sediments, tracking vegetation change during (green Sahara) episodes of the Pleistocene (39). Taken together, this evidence demonstrates that sedimentary plant-wax distributions can be used to track large-scale shifts in vegetation structure in Africa.

**Fig. 3.**
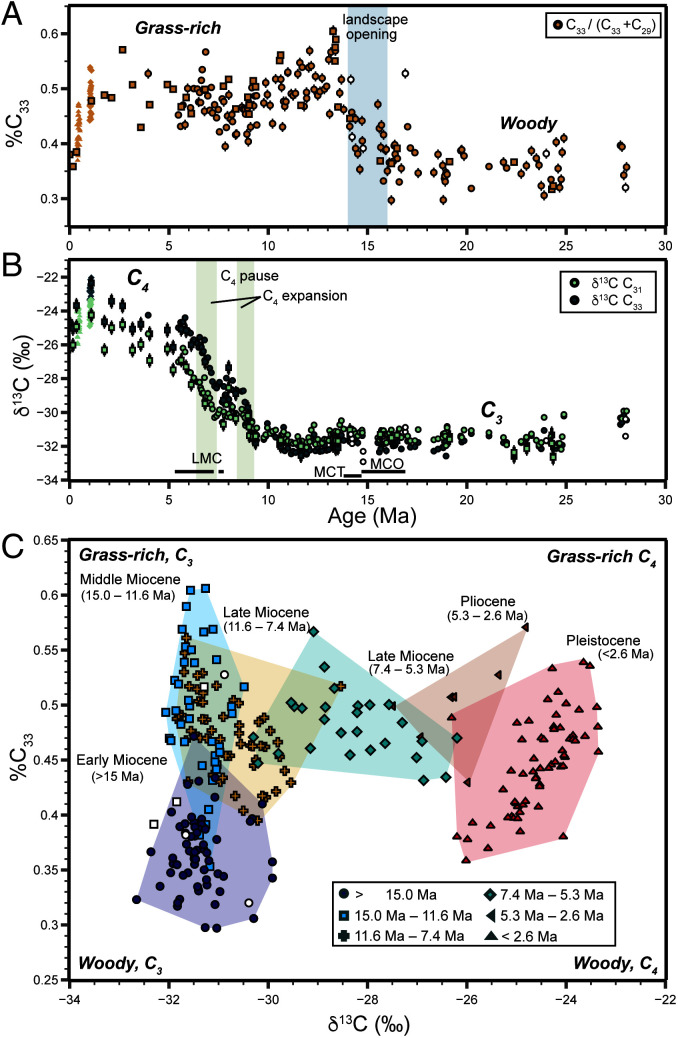
Distribution and carbon isotope data showing C_3_ open vegetation development in the middle Miocene (*A*), and stepwise increase of C_4_ vegetation during the late Miocene (*B*) integrating new data and that of Polissar et al. (8), O’Mara et al. (38), and O’Mara et al. (39). Datasets in (*A* and *B*) are indicated with symbol shape as follows: circles = new data; squares = Polissar et al. (8); diamonds = O’Mara et al. (38); triangles = O’Mara et al. (39). Cross plot (*C*) shows the evolution of the NW African landscape from woody, C_3_ vegetation in the late Oligocene to early Miocene through grass-rich, C_3_ vegetation in the middle Miocene, and the transition to grass-rich, C_4_ vegetation in the late Miocene. Major age periods are indicated with differently styled and colored symbols. Samples with CPI < 3.0 indicated with open symbols in all plots. All other abbreviations as in Fig. 1.

Our new *n*-alkane distribution record exhibits a dramatic shift between ~16 and 14 Ma from shorter chain lengths (greater relative abundance of woody vegetation) to longer chain lengths (greater relative abundance of grasses and forbs), suggesting NW African open ecosystem expansion in the middle Miocene (Fig. 3). We integrated the lower-resolution record of Polissar et al. (8) with our new data and use two-tailed *t* tests of the integrated dataset to show that samples older than 16 Ma have overall lower values, while those after 14 Ma are significantly higher (%C_31_: *P* = 3.83 × 10^−54^; %C_33_: *P* = 3.84 × 10^−48^; %C_35_: *P* = 1.89 × 10^−12^; *SI Appendix*, Fig. S12). Samples between 16 and 14 Ma are largely transitional between the two. To determine when open ecosystems started expanding, we compared mean %C_33_ values in moving 1-My windows against the early Miocene 17 to 23 Ma mean. Sample windows begin to exhibit *P* values below 0.05 at ~16 Ma (two-sample *t* test), indicating ecosystem opening may have begun as early as this age. By 15 Ma, sample bins always have *P* values less than 0.05, marking the unequivocal opening of the landscape (*SI Appendix*, Fig. S13 and section S9). From 14 Ma through the late Miocene, the distributions of %C_31_, %C_33_, and %C_35_ remain elevated above that of the mean early Miocene value, indicating that open ecosystems persisted on the landscape and never returned to the more tree- and shrub dominated plant communities of the middle- and early Miocene. Subsequent decreases in %C_33_ (and C_31_ δ^13^C) observed in Fig. 3*C* are consistent with an expanded, nonvegetated Sahara Desert, constraining grass-rich ecosystems closer to the tropics during dry, glacial periods (59, 85–87). These interpretations assume that the relationships observed in modern through Pleistocene *n*-alkane distributions and their correlation with growth form persists in older taxa. Our multihomolog *n*-alkane δ^13^C record (see below) support this assumption: C_4_ vegetation, which is herbaceous, exhibits chain length distributions skewed toward long chain components since at least 10 Ma, suggesting continuity in the *n*-alkane distribution of growth forms through geologic time.

### *n*-Alkane Carbon Isotope Ratios.

The carbon isotope composition of long chain *n*-alkanes for all NW African sediment samples demonstrates a shift from C_3_-dominated vegetation prior to 10 Ma into C_4_-dominated vegetation by the Pliocene (Fig. 3). Modern African C_3_ plants produce odd, long chain *n*-alkanes whose δ^13^C values are considerably more negative than those of C_4_ plants (e.g., mean δ^13^C_31_ of C_3_ plants = −33.4 ‰, compared to mean δ^13^C_31_ of C_4_ plants = −19.9 ‰) (7 and references therein). The carbon isotopic composition of NW African sedimentary *n*-alkanes (C_29_ to C_35_, odd) ranges from −35.9 to −23.3‰, reflecting mixtures of C_3_ and C_4_ plant sources.

Late Oligocene to middle Miocene δ^13^C values are consistent with a C_3_ vegetation source (C_29_ to C_35_ odd, mean = −31.1 ± 0.8‰). Beginning after 11 Ma, δ^13^C values become progressively more positive, reflecting increasing proportions of C_4_ vegetation and the spread of C_4_ grasslands and savanna ecosystems. This change is particularly evident in the δ^13^C of *n*-C_33_ and *n*-C_35_ (Fig. 3 and *SI Appendix*, Fig. S15) and is also consistent with a herbaceous source with higher abundances of long-chain *n*-alkanes. Our new higher-resolution record reveals a complex development of NW African C_4_ vegetation, a pattern that was not discernable in previous, low-resolution records (8, 59). During the late Miocene, we observe two periods of accelerated C_4_ expansion separated by slowed growth (*SI Appendix*, Fig. S14). We establish the age of these periods from a locally weighted scatterplot smoothing (lowess) curve of the new δ^13^C data combined with that of Polissar et al. (8), using first and second derivatives to identify transitions between rapid and slow δ^13^C change (*SI Appendix*, Fig. S14 and section S10). Our analysis shows δ^13^C values for the most abundant *n*-alkane (C_31_) increase between ~9.3 and 8.4 Ma, slow between 8.4 and 7.4 Ma, and increase again until the end of the Miocene (7.4 to 5.3 Ma). Assuming a relatively stable geographic source area, the greatest rate of C_4_ expansion occurs from 7.4 to 6.4 Ma, with periods of smaller increase or pauses prior to that. These patterns are found in all plant-wax chain lengths at similar times and demonstrate that the expansion of C_4_ vegetation was pulsed rather than monotonic.

## Discussion

### Middle Miocene Development of C_3_ Open Ecosystems.

Our combined *n*-alkane distribution and carbon isotope record shows that open, grass- or forb-rich ecosystems arose in the middle Miocene (~16 Ma), predating the expansion of C_4_ grasses by approximately 5 My in NW Africa (Fig. 3). These novel, C_3_ open ecosystems began spreading during the Miocene Climatic Optimum (16.9. to 14.7 Ma) and accelerated in the Miocene Climate Transition (14.7 to 13.8 Ma), a period of global climate aridification and cooling (41). These middle Miocene NW African ecosystems have no modern analog as herbaceous, C_3_ open ecosystems are not found in subtropical, low elevation regions of Africa today. Our data therefore place new constraints on the history of ecosystem assembly for the region: Early Miocene C_3_ woody vegetation was replaced by C_3_ grasslands or savannas, and C_4_ open vegetation replaced C_3_ grasslands or savannas instead of C_3_ closed woodlands and forests. These patterns suggest that the C_4_ photosynthetic pathway is not a precondition for open, subtropical vegetation to be widespread in Africa. Further, the combination of climate, consumer, and plant trait interactions that allowed for C_3_ open vegetation to proliferate in the middle Miocene was distinct from the drivers of the later spread of C_4_ grasslands and savannas.

Our findings are consistent with other vegetation records from Africa showing the prevalence of closed ecosystems in the early Miocene and the later occurrence of extensive, open vegetation. Additionally, ours is the first continuous record (to our knowledge) that spans the Miocene and precisely determines the timing of the origination and demise of this ecosystem. In the early Miocene, low values of %C_33_ indicate the dominance of closed forests and woodlands. This matches much of the pollen and macrofossil evidence from the early Miocene of east, north, and tropical west Africa, which point to the widespread existence of dry forests and woodlands, coastal swamplands, and mangrove forests to megathermal tropical forests (12–17). East African phytoliths and δ^13^C data indicate locally abundant PACMAD grasses, including likely C_4_ chloridoids by 18 Ma, but overall suggest a heterogeneous landscape that included forest, with little indication of extensive open grasslands (24, 88). The middle Miocene origination of C_3_ savannas that we observe in our record is similarly consistent with low to medium abundances of grass pollen in coarsely dated early- and middle-Miocene sediments from the Niger Delta and high abundances of grass pollen from Saudi Arabia during the same time interval (27, 89, 90). Studies combining *n*-alkane biomarkers and pollen assemblages provide some of the best evidence of Miocene C_3_ grass-rich, open ecosystems. For example, grass pollen abundances in east Africa are as high as 40% at ~11 Ma (the maximum age of this record) whereas plant-wax biomarker δ^13^C values indicate C_3_ vegetation (18, 30). Likewise, in southwest Africa, the oldest available samples (8 to 9 Ma) contain 30 to 40% Poaceae pollen, but C_3_ *n*-alkane δ^13^C values (28, 29). Interpretations of these grass-rich ecosystems range from low-lying swamplands, woodlands with a grassy understory, to savanna with a C_3_ grass layer (12, 15, 18, 27–29, 90). In sum, previous work documents dominantly closed woody (C_3_) vegetation across much of Africa (although with locally abundant grasses) in the early—middle Miocene and the subsequent spread of herbaceous C_3_ vegetation. However, these studies were unable to date the transition. Our new *n*-alkane record locates the rapid shift between these vegetation types in NW Africa between 15 and 14 Ma, during the Miocene Climate Transition.

### The Late Miocene Development of C_4_ Grasslands and Savannas.

**Fig. 4.**
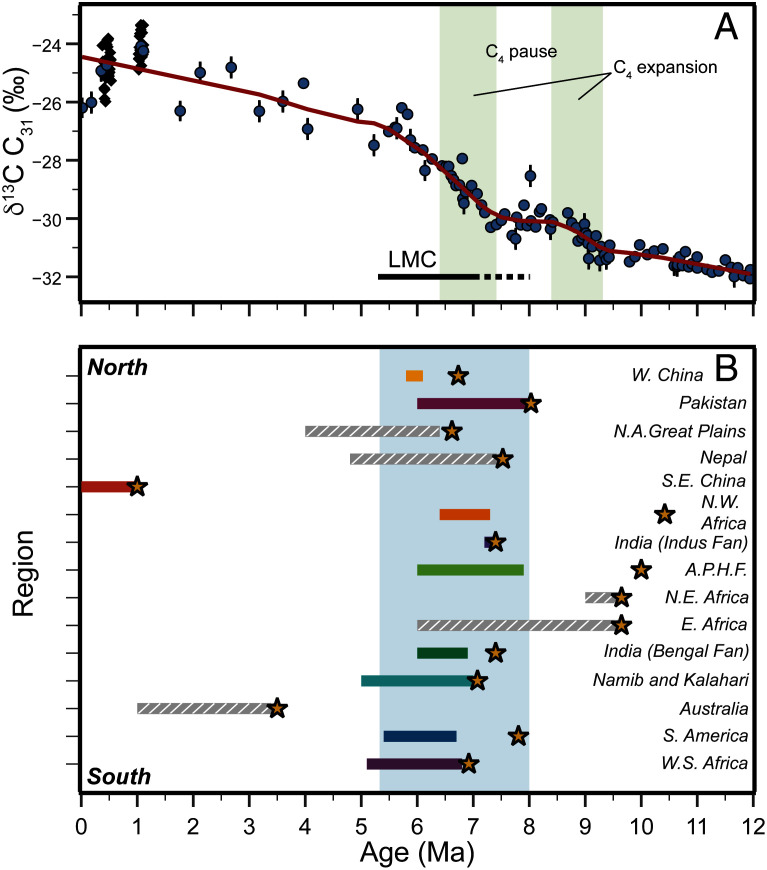
Development of widespread C_4_ vegetation in the late Miocene of northwest Africa (*A*) and other tropical and subtropical regions (*B*). In (*A*), carbon isotope ratios of C_31_ *n*-alkane from this study and Polissar et al. (8) are shown with blue circles. Orbitally resolved data (38, 39) are shown with black diamonds. The red line is a locally weighted scatter plot smoothing curve fit to the data as described in the caption of Fig. 1. Green bars show the inferred ages of rapid C_4_ spread. (*B*) Estimated timing of the most rapid expansion of C_4_ vegetation for each region is shown with solid-colored bars, ordered from most northerly latitude to most southerly latitude. Earliest inception of C_4_ vegetation expansion is shown with yellow stars. White hashes indicate that data are from localities with less than three samples per 1 My during the time of expansion, making estimation of timing difficult. The light blue box indicates the timing of the Late Miocene Cooling (LMC), as described in the caption of Fig. 1. Data source, selection, and analysis described in *SI Appendix*, section S13. W. China = west China; N.A. Great Plains = North American Great Plains; S.E. China = southeast China; N.W. Africa = northwest Africa; A.P.H.F = Arabian Peninsula/Himalayan foreland; N.E. Africa = northeast Africa; E. Africa = east Africa; S.W. Africa. = southwest Africa; S. America = South America; W.S. Africa = western South Africa.

In our new, high-resolution record, the late Miocene is marked by periods of stepwise C_4_ ecosystem expansion during global climate cooling and aridification, potentially linking climate conditions and C_4_ savanna development. Following the beginning of C_4_ ecosystem expansion after 11 Ma (*SI Appendix*, section S11), an initial pulse of C_4_ expansion is evident in increases in the δ^13^C values between ~ 9.3 and 8.4 Ma. This is mirrored in the plant-wax δ^13^C record from Site 231 offshore east Africa, suggesting that this increase in C_4_ vegetation was likely occurring across northern Africa (18). In east Africa, pollen evidence suggests that grass abundances were high and stable (~30% of total pollen counts) through this time period, although data points are sparse, and that more arid adapted and potentially C_4_ forbs (Chenopodioideae or other Amaranthaceae) were proliferating (18, 30). In NW Africa, δ^13^C values increase first in C_35_ and C_33_ followed by C_31_ and C_29_ which is consistent with a C_4_ grass source, although increases in other C_4_ plants with grass-like plant-wax distributions (e.g., certain C_4_ forbs) cannot be precluded. The second and greatest increase in C_4_ vegetation suggested by our record occurs between ~7.4 and 6.4 Ma, coincident with expansions of C_4_ ecosystems across part of the African continent and the subtropics generally (Fig. 4). For example, in the southwestern region of Africa, C_4_ vegetation and grasses expanded between ~7.0 and 5.0 Ma (28, 29, 31), and the abundances of grass pollen increased between 7.4 and 6.4 Ma in tropical west Africa (27) although the photosynthetic pathways of these grasses are not known. In Central Africa, phytolith assemblages suggest grass dominated ecosystems were present on the landscape since at least 7.0 to 7.5 Ma, and carbon isotope ratios of mammal teeth indicate dominantly C_4_ diets for some herbivores (6.0 to 5.0 Ma), consistent with an expanding C_4_ savanna (91, 92).

The late Miocene increases in %C_4_ across Africa is mirrored in many other subtropical regions, such as Nepal (~7.5 Ma), the Indus and Bengal Fans (~7.4 and 6.9 Ma, respectively), Argentina (~6.7 Ma), North American Great Plains (~6.4 Ma), and the Chinese Loess Plateau (~6.1 Ma) (4, 7, 8, 11, 93–95), although there are exceptions, notably Australia (~3.5 Ma) (3). This interval is roughly coincident with the LMC, which spanned 7.0 to 5.3 Ma, but began perhaps as early as 8.0 Ma, and is characterized by global SST declines and the steepening of the pole-to-equator temperature gradient (40, 41). The spread of subtropical C_4_ ecosystems co-occurring with this period of climate reorganization points to the potential for a global mechanism driving the increase in competitiveness of these ecosystems in the latest Miocene (see discussion below in “*Causes of Miocene Ecosystem Turnover*”).

The 5 My over which C_4_ vegetation increased to become the primary herbaceous component of savanna and grassland ecosystems in NW Africa was unlikely to have been the product of a simple replacement of C_3_- for C_4_ grasses. Rather, we propose that the long-term spread of C_4_ vegetation is the result of complex turnover of plant communities with unique species composition, traits, and interactions (96, 97). To test this hypothesis, we investigated (dis)continuity in late Miocene to present C_3_- and C_4_ plant communities with a coupled analysis of the plant-wax distribution and δ^13^C records. We separated the *n*-alkane distributions for the C_3_- and C_4_ components of the sedimentary *n*-alkane archive from 9.0 Ma to Recent by apportioning a fraction of each chain length’s abundance based upon its measured δ^13^C value compared to C_3_- and C_4_ δ^13^C values of modern plants (*SI Appendix*, section S12). The resultant *n*-alkane distributions show distinct C_3_- and C_4_ components broadly similar to woody vegetation (C_3_ component) and grasses/forbs (C_4_), respectively. Metric dimensional analyses of the C_3_- and C_4_ components reveals clustering of distributions through time: Pleistocene plant-wax distributions, whether C_3_ or C_4_, always cluster separately from the Miocene distributions, while C_3_ Pliocene distributions cluster with C_3_ Miocene distributions and C_4_ Pliocene distributions cluster with C_4_ Pleistocene distributions (*SI Appendix*, Fig. S17). These results suggest that the relative composition of plant communities of the late Miocene savanna differed from those of the Pleistocene and today, even if we do not know the detailed taxonomic make-up of the C_3_- and C_4_ components of vegetation. This distinction is significant, because species within a particular functional group (e.g., grasses) can differ in plant traits that determine their interactions with climate, fire, and other processes (98–100). Therefore, the strength of the feedbacks operating between climate, consumers, and plant traits that caused the late Miocene expansion of C_4_ savanna ecosystems were not necessarily the same as those that maintain their existence on the landscape today.

### Causes of Miocene Ecosystem Turnover.

What caused open vegetation assembly and turnover in the Miocene of NW Africa? Answering this question has proved challenging because of the complex interactions that control the ecological success of open ecosystems, and the lack of proxy data for the region (8, 12, 59, 101). In modern subtropical Africa, a system of climate conditions and consumer dynamics interact with each other and with plant traits to promote the C_4_ grass-dominated ecosystems (56, 57, 102, 103). C_4_ savannas in Africa are promoted by low to medium rainfall regimes that are highly seasonal, and low (at least prior to the preindustrial) atmospheric CO_2_ concentrations (42, 43, 56, 103). However, it is the interaction of these conditions with consumer dynamics that maintain C_4_ savanna ecosystems at their current distribution. In particular, both herbivory and frequent fires are required to limit the establishment of forest and woodland C_3_ trees and shrubs, while promoting both C_4_ grasses and C_3_ savanna tree- and shrub taxa that are well adapted to these disturbances (44, 45, 100). Climate affects these disturbance mechanisms and specific plant traits can encourage or preclude different fire and herbivory regimes. For example, a highly seasonal climate causes grass dieback and fuel load accumulation, which promotes frequent fires. Grasses that promote fire are tall, can resprout frequently, and amass large amounts of biomass above-ground, while grasses associated with high levels of herbivory are much shorter and burn less easily (44, 45, 100). Because of these nonlinear feedbacks, small changes in climate or consumer patterns can lead to large changes in ecosystem distribution (104).

Although we currently do not have the data to decisively test whether a combination of climate- and consumer mechanisms were active also during the Miocene, available evidence is suggestive of a complex set of drivers. The coincidence of middle Miocene C_3_ landscape opening in NW Africa with extensive global climate change suggests that climate played a role in initiating C_3_ open ecosystem expansion. Our new record points to the spread of grass-rich C_3_ landscapes beginning perhaps as early as 16 Ma during the Miocene Climatic Optimum and accelerating during the cooling and aridification of the Miocene Climate Transition. We hypothesize that the region experienced increasing aridity and seasonality during the Miocene Climate Transition when we observe the greatest shift in our record (41, 105–107). Such climatic changes could have helped limit the range of forest and woodland vegetation and opened up space for C_3_ grasses, trees, and shrubs that were better adapted to limited and/or seasonal rainfall to expand. Concordant with this hypothesis, changes to hydroclimate during this interval in north Africa are supported by several global climate model experiments. Declining CO_2_ coupled to both the closure of the Tethys seaway, now estimated to have ended during the Miocene Climate Transition (41), and progressive uplift of east Africa have been shown to increase aridity and decrease vegetation cover in north Africa (108–110). In particular, the closure of the Tethys seaway appears to be a strong driver of aridity in the region. Replacing the Tethys seaway with the Arabian peninsula decreases atmospheric convergence over north Africa and shifts moisture to south Asia, an effect that would be magnified by uplift of the Tibetan Plateau (109–111). Additionally, community earth system modeling (109) demonstrates that both the closure of the Tethys and declining *p*CO_2_ through the Miocene Climate Transition would lead to heightened seasonality in precipitation during the Miocene Climate Transition. Even relatively minor changes to the seasonality or precipitation regime for the region could have promoted widespread landscape opening once the feedbacks associated with fire or herbivory, also promoted by the limited or seasonal rainfall, became stronger. However, the only study from Africa using plant-wax δ^2^H does not have the sample resolution to interrogate hydroclimate change during the middle Miocene, rendering our interpretations above somewhat speculative (8).

There is evidence that consumers may have played a part in the NW African middle Miocene vegetation shift, potentially amplifying the effects of regional climate changes. Increased incidence of charred grass cuticle in middle Miocene Niger Delta sediments co-occurring with higher relative abundances of grass pollen suggest that these grass-rich ecosystems may have burned—a feedback that maintains seasonally dry savannas at their current extent today (27). The African faunal record is spotty but shows that mammalian lineages that today are associated with high-intensity herbivory in subtropical Africa were present and diverse since the early Miocene (<20 Ma), although most were C_3_ browsers (21). Phylogenetic analysis further demonstrates parallel diversification of African bovids and spiny plants (a common feature of savanna woody plants and presumed to be an adaptation to herbivory) during the middle Miocene, consistent with the existence of a C_3_ savanna ecosystem driven by increased browser pressure (34).

The step-wise increase in C_4_ vegetation in NW Africa spans the late Miocene, when global climate is characterized by progressive cooling, aridification, and increased pole-to-equator temperature gradients (40, 41). This global cooling culminated in the LMC, a climate feature most apparent in reconstructions of SST (40) and during which many of the world’s subtropical ecosystems, including NW Africa, saw increased rates of C_4_ spread (Fig. 4 and *SI Appendix*, section S13). This widespread pattern points to a global, or cross-regional, driver for the C_4_ expansion of the latest Miocene—such as declining CO_2_ or decreased and more seasonal precipitation—which would favor C_4_ plants due to decreased rates of photorespiration and improved water use efficiencies compared to C_3_ plants.

A global or regional climate mechanism associated with the LMC that would directly translate into the spread of C_4_ ecosystems in NW Africa is difficult to determine from current records. First, reconstructions of atmospheric CO_2_ vary widely for the late Miocene (112), rendering the effect of CO_2_ on ecosystem change ambiguous. While some records point to declining CO_2_ during the LMC (113–115), many others do not (112). Until better constraints are placed on paleo-*p*CO_2_ for the Late Miocene, the effects of this driver on global C_4_ ecosystems will remain uncertain. Second, analyses of regional hydroclimate for NW Africa, which have focused exclusively on overall rainfall, show conflicting trends (8, 59, 110). For example, studies using Earth system- and atmospheric models tend to reconstruct increasing overall aridity from the middle Miocene through Pleistocene for North Africa, although these models are forced by assumed parameters such as sea surface temperatures, topography and *p*CO_2_ (110, 116, 117). Drying in the late Miocene would have resulted from the shrinkage of the Tethys seaway weakening the African summer monsoon, driving moisture convergence to the south, and increasing the strength of the Somali Jet (part of the western arm of the southeast Asian monsoon) (110). However, these links hinge on the timing of the Tethys closure, which was recently placed in the early–middle Miocene (41). Sedimentological study of marine and terrestrial deposits for north Africa suggests that the region experienced orbitally controlled, wet and dry intervals through the entire late Miocene, with the Sahara Desert likely present in some form since at least 11 Ma (59, 87, 118). Several of these records even infer increased precipitation on the north African continent during the LMC (59, 118). In contrast, low-resolution records of plant-wax hydrogen isotopes from sites offshore tropical, north and east Africa find no evidence for a multimillion-year change to the precipitation that plants experienced during the time when C_4_ vegetation would have been expanding (8). Third, the influence of precipitation seasonality, an equally important control on vegetation structure and composition (119, 120), has yet to be directly tested for north Africa. Finally, it is not known if other plant traits associated with the C_4_ pathway, such as fast growth- and curing rates, nitrogen reallocation strategies between light and dark reactions, or high nitrogen use efficiencies promoted the competitive edge of C_4_ grasses during the late Miocene (49, 53, 97). Overall, although C_4_ expansion occurred during periods of global climate change, the specific climate drivers in NW Africa are not readily apparent, a fact that in and of itself might point to the influence of multiple drivers and feedbacks.

The role of consumers, and their interactions with climate and plant traits may also be a crucial part of the explanation for the rise to dominance of C_4_ open ecosystems in the late Miocene. Geoxyles, underground woody structures that allow for rapid resprouting and a potential adaptation to frequent fires (22) diversified in tandem with the late Miocene spread of C_4_ ecosystems, supporting a link between fires and C_4_ fire prone savannas. In the southwestern region of Africa, charcoal abundance in a deep-sea drill core also tracks increases in C_4_ vegetation through the LMC, tying the expansion of the C_4_ savannas to frequent fires in this region and time period (29). After 10 Ma, an increasing number of large-mammal lineages in east Africa adopted C_4_ grazing diets, including equids, rhinos, and bovids, pointing to increased herbivore pressure on grassy vegetation (20).

Our new vegetation history from NW Africa shows that the initial development of nonanalog, C_3_ open ecosystems in the middle Miocene and the late Miocene transition into C_4_ savanna both occurred during well-documented periods of global cooling and aridification, which may have translated into regional hydroclimate change (59, 109, 110), providing a possible link between climate change and ecosystem turnover. In addition, records of African herbivory and fire dynamics indicate that consumers also influenced vegetation structure during the assembly of these ecosystems. Thus, our new data evaluated in the context of previously published work point to the same set of controls that govern modern African savannas—climate, fire, herbivory, and plant adaptation—although their relative importance during the Miocene is unknown. It is also not clear if current paleoproxies would allow for detection of important thresholds in climate or consumer parameters that led to ecosystem change (although important advances have recently been made in quantifying herbivore diversity and abundance on the landscape, see refs. 121 and 122). Instead, we echo a previous suggestion that studying the evolution and proliferation of specific plant functional traits could provide insights into the forcings that these plants were responding to and ultimately help better understand the drivers of open ecosystem assembly (97). We also propose that adding other regional Miocene vegetation records to ours (which has a biomarker source area of ~10 to 25°N in the modern), thereby constraining the timing and pattern of vegetation change across latitudes (84), would allow further testing for drivers in the expansion of widespread open C_3_ and C_4_ ecosystems on the North African continent.

Our new findings help build the case that the strong feedbacks between climate, consumers, and plant traits that act on subtropical ecosystems can cause landscape transformation even under modest, relative to modern, global climate perturbations (41, 123). Because future climate change is projected to equal or exceed these Miocene changes in magnitude (e.g., in atmospheric CO_2_ concentrations and global mean surface temperatures), the geologic record indicates that a dramatic reshaping of subtropical open ecosystems could occur, with the outcome depending upon the nature and strength of the feedbacks operating on these ecosystems, including those of human systems (112, 123).

## Materials and Methods

### Sampling and Age Control.

Both marine sediment cores used in this study (ODP 659 and DSDP 368, ~500 km off the continental margin on the Cape Verde Plateau and Rise) capture a near complete sedimentary record of the southern North Atlantic and NW African continent from the late Oligocene through end-Miocene. Sedimentation through this time interval consists primarily of nannofossil oozes, with some lithogenic components (62, 63). Study sites are located underneath the African Easterly Jet and Saharan Air layer, which carry dust from the NW African continent over the Atlantic Ocean and are the likely source of much of the biomarker deposition (84, 124). Source area for biomarker deposition is interpreted as approximately the continental region of northwest and northcentral Africa between the modern latitudes of ~10 to 25°N. This is based on modern biomarker deposition, vegetation distributions, and atmospheric air parcel trajectories, as well as radiogenic isotope signatures of dust from ODP 659 from 11 Ma to the Recent (59, 84, 125). We sampled ODP 659 sediments for biomarker analysis every 100 kyr from ~5 to 17 Ma, and at a ~250 ky resolution to the base of the core (late Oligocene) due to lower overall sedimentation rates through this interval. We additionally analyzed ~40 samples spanning 10 to 20 Ma from DSDP 368 to 1) provide a replicate of ODP 659 and 2) cover minor gaps in the sedimentary record suggested by the initial reports volume (62, 63). We requested 60 ml of core material per sample from both sites (mean sample mass = ~77 g), and temporal integration ranges from 1.4 to 86.8 ky, with an average value of 8.3 ky.

We established age control at ODP 659 by extending the orbitally tuned late Miocene through Recent age model published in Crocker et al. (59) with five late Oligocene through middle Miocene nannofossil biohorizons (8). To do this, we used the composite depth scale of Crocker et al. (59) for all of hole C, and the majority of holes B and A. The last tie-points of this composite depth scale are at 185.42 and 199.40 m below sea floor in holes A and B respectively, and we used the composite depth scale of Polissar et al. (8) for those samples that fell below these depths. Sample ages were then calculated by linear interpolation between age-depth tie-points. For DSDP 368, we used eight nannofossil zonations and one radiolarian biohorizon described in the initial reports volume for the single hole (Z) to create an age model (62). Due to lack of fossil preservation and identification, boundaries between biohorizons were difficult to identify. We therefore used averaged nannofossil zonation ages, and in one case the age range of a specific taxon, updated using the chronology of Backman et al. (126) and Raffi et al. (127), and the averaged sediment depths for those identified nannofossil zones to calculate age-depth tie-points. These tie-points were then used to calculate sample ages by linear interpolation. Biomarker analyses (distribution metrics and carbon isotope ratios) from both core sites agree well. See *SI Appendix*, sections S1 and S2 for additional details on the study site and chronology. Age model data for both sites are reported in Dataset S1.

### Plant Wax *n*-Alkane Extraction and Quantification.

Sediment samples were freeze-dried, crushed, and extracted by accelerated solvent extraction on an ASE 350 in 9:1 dichloromethane:methanol. After the addition of a recovery standard, extracts were purified by aminopropyl and silica gel column chromatography to separate extracts into an aliphatic, ketone, and polar fraction. Quantification of the aliphatic fraction was performed by gas chromatography mass spectrometry on an Agilent 7890A GC interfaced to an Agilent 5975B MS. Compounds were identified by retention time and fragmentation pattern and quantified with external calibration of *n*-alkane response factors. Detailed methodology is described in *SI Appendix*, section S3, and all replicate analyses are reported in Dataset S2.

### Compound-Specific δ^13^C Analysis.

Compound specific carbon isotope analysis was completed using gas chromatography, isotope ratio mass spectrometry by combustion reduction (Thermo Instruments, Delta V advantage connected by a ConFlo IV to a GC Ultra and Isolink device). Samples were run in duplicate or triplicate and a molecular standard (Mix A, Arndt Schimmelmann, University of Indiana) was run every 2 to 5 samples to convert values to the VPDB scale and calculate uncertainties (128). Raw δ^13^C values of C_25_ to C_35_ were corrected for changes in the δ^13^C composition of atmospheric CO_2_ through time following methods outlined in Polissar et al. (8) using the benthic foraminifera-based CO_2_ carbon isotope reconstruction of Tipple et al. (129) (*SI Appendix*, section S5). Raw and corrected values are reported in Dataset S3.

### Modern Plant Compilation and Analysis.

We compiled 13 datasets of modern African plant *n*-alkane concentrations to investigate the link between growth form and compound distributions (*SI Appendix*, section S7; 66–78). We first identified taxa to the family, genus, and species level using the Taxonomic Name Recognition Service database (130) to match reported taxon information to accepted nomenclature. We calculated %C_31_, %C_33_, and %C_35_ for all samples and averaged results by species to remove the effect of weighting toward overrepresented taxa, leaving 616 samples for statistical evaluation. Using two-sample Kolmogorov–Smirnov tests, we pairwise compared distributions of %C_31_, %C_33_, and %C_35_ of trees, shrubs, forbs, and grasses to test whether the *n*-alkane metric values of growth forms derived from the same underlying distribution. Methodology is described in detail in *SI Appendix*, section S7 and results of the statistical tests are shown in *SI Appendix*, Table S1.

## Supplementary Material

Appendix 01 (PDF)

Dataset S01 (XLSX)

Dataset S02 (XLSX)

Dataset S03 (XLSX)

## Data Availability

Study data are included in the article and/or supporting information.

## References

[1] C. A. E. Strömberg, A. C. Staver, The history and challenge of grassy biomes. Science **377**, 592–593 (2022). 35926015 10.1126/science.add1347

[2] E. Buisson, S. Archibald, A. Fidelis, K. N. Suding, Ancient grasslands guide ambitious goals in grassland restoration. Science **377**, 594–598 (2022). 35926035 10.1126/science.abo4605

[3] J. W. Andrae , Initial expansion of C_4_ vegetation in Australia during the late Pliocene. Geophys. Res. Lett. **45**, 4831–4840 (2018).

[4] S. J. Feakins , Miocene C_4_ grassland expansion as recorded by the Indus Fan. Paleoceanogr. Paleoclimatol. **35**, e2020PA003856 (2020).

[5] D. L. Fox , Climatic controls on C_4_ grassland distributions during the Neogene: A model-data comparison. Front. Ecol. Evol. **6**, 147 (2018).

[6] A. T. Karp, J. W. Andrae, F. A. McInerney, P. J. Polissar, K. H. Freeman, Soil carbon loss and weak fire feedbacks during Pliocene C_4_ grassland expansion in Australia. Geophys. Res. Lett. **48**, e2020GL090964 (2021).

[7] P. J. Polissar , Hydrologic changes drove the late Miocene expansion of C_4_ grasslands on the northern Indian subcontinent. Paleoceanogr. Paleoclimatol. **36**, e2020PA004108 (2021).

[8] P. J. Polissar, C. Rose, K. T. Uno, S. R. Phelps, P. deMenocal, Synchronous rise of African C_4_ ecosystems 10 Mya in the absence of aridification. Nat. Geosci. **12**, 657–660 (2019).

[9] C. A. E. Strömberg, Evolution of grasses and grassland ecosystems. Annu. Rev. Earth Planet. Sci. **39**, 517–544 (2011).

[10] C. A. E. Strömberg, F. McInerney, The Neogene transition from C_3_ to C_4_ grasslands in North America: Assemblage analysis of fossil phytoliths. Paleobiology **37**, 50–71 (2011).

[11] L. Tauxe, S. J. Feakins, A reassessment of the chronostratigraphy of late Miocene C_3_–C_4_ transitions. Paleoceanogr. Paleoclimatol. **35**, e2020PA003857 (2020).

[12] B. Jacobs, A. Pan, C. Scotese, “A review of the Cenozoic vegetation history of Africa” in Cenozoic Mammals of Africa, L. Werdelin, Ed. (University of California Press, 2010), pp. 57–72.

[13] B. F. Jacobs, J. D. Kingston, L. L. Jacobs, The origin of grass-dominated ecosystems. Ann. Mo. Bot. Gard. **86**, 590–643 (1999).

[14] R. J. Morley, Origin and Evolution of Tropical Rain Forests (John Wiley and Sons Ltd., Chichester, United Kingdom, 2000).

[15] H. P. Linder, East African Cenozoic vegetation history. Evol. Anthropol. **26**, 300–312 (2017). 29265653 10.1002/evan.21570

[16] H. P. Linder, The evolution of African plant diversity. Front. Ecol. Evol. **2**, 38 (2014).

[17] J. Maley, The African rain forest—main characteristics of changes in vegetation and climate from the upper cretaceous to the quaternary. Proc. R. Soc. Edinb. Sec. B Biol. Sci. **104**, 31–73 (1996).

[18] S. J. Feakins , Northeast African vegetation change over 12 My. Geology **41**, 295–298 (2013).

[19] K. T. Uno, P. J. Polissar, K. E. Jackson, P. B. deMenocal, Neogene biomarker record of vegetation change in eastern Africa. Proc. Natl. Acad. Sci. U.S.A. **113**, 6355–6363 (2016). 27274042 10.1073/pnas.1521267113 PMC4988583

[20] K. T. Uno , Late Miocene to Pliocene carbon isotope record of differential diet change among East African herbivores. Proc. Natl. Acad. Sci. U.S.A. **108**, 6509–6514 (2011). 21464327 10.1073/pnas.1018435108 PMC3080981

[21] J. T. Faith, J. Rowan, A. Du, Late Cenozoic faunal and ecological change in Africa. Annu. Rev. Earth Planet. Sci. **52**, 379–407 (2024).

[22] O. Maurin , Savanna fire and the origins of the “underground forests” of Africa. New Phytol. **204**, 201–214 (2014). 25039765 10.1111/nph.12936

[23] S. Halali, P. M. Brakefield, S. C. Collins, O. Brattström, To mate, or not to mate: The evolution of reproductive diapause facilitates insect radiation into African savannahs in the late Miocene. J. Anim. Ecol. **89**, 1230–1241 (2020). 31955425 10.1111/1365-2656.13178

[24] D. J. Peppe , Oldest evidence of abundant C_4_ grasses and habitat heterogeneity in eastern Africa. Science **380**, 173–177 (2023). 37053309 10.1126/science.abq2834

[25] D. P. Dugas, G. J. Retallack, Middle Miocene fossil grasses from Fort Ternan, Kenya. J. Paleontol. **67**, 113–128 (1993).

[26] T. E. Cerling, J. M. Harris, S. H. Ambrose, M. G. Leakey, N. Solounias, Dietary and environmental reconstruction with stable isotope analyses of herbivore tooth enamel from the Miocene locality of Fort Ternan, Kenya. J. Hum. Evol. **33**, 635–650 (1997). 9467773 10.1006/jhev.1997.0151

[27] R. J. Morley, K. Richards, Gramineae cuticle: A key indicator of late Cenozoic climatic change in the Niger Delta. Rev. Palaeobot. Palynol. **77**, 119–127 (1993).

[28] S. Hoetzel, L. M. Dupont, G. Wefer, Miocene-Pliocene vegetation change in south-western Africa (ODP site 1081, offshore Namibia). Palaeogeogr. Palaeoclimatol. Palaeoecol. **423**, 102–108 (2015).

[29] S. Hoetzel, L. Dupont, E. Schefuß, F. Rommerskirchen, G. Wefer, The role of fire in Miocene to Pliocene C_4_ grassland and ecosystem evolution. Nat. Geosci. **6**, 1027–1030 (2013).

[30] R. Bonnefille, Cenozoic vegetation, climate changes and hominid evolution in tropical Africa. Glob. Planet. Change **72**, 390–411 (2010).

[31] L. M. Dupont, F. Rommerskirchen, G. Mollenhauer, E. Schefuß, Miocene to Pliocene changes in South African hydrology and vegetation in relation to the expansion of C_4_ plants. Earth Planet. Sci. Lett. **375**, 408–417 (2013).

[32] F. Bibi, A multi-calibrated mitochondrial phylogeny of extant Bovidae (Artiodactyla, Ruminantia) and the importance of the fossil record to systematics. BMC Evol. Biol. **13**, 166 (2013). 23927069 10.1186/1471-2148-13-166 PMC3751017

[33] Y. Bouchenak-Khelladi, O. Maurin, J. Hurter, M. van der Bank, The evolutionary history and biogeography of Mimosoideae (Leguminosae): An emphasis on African acacias. Mol. Phylogenet. Evol. **57**, 495–508 (2010). 20696261 10.1016/j.ympev.2010.07.019

[34] T. Charles-Dominique , Spiny plants, mammal browsers, and the origin of African savannas. Proc. Natl. Acad. Sci. U.S.A. **113**, E5572–E5579 (2016). 27601649 10.1073/pnas.1607493113 PMC5035896

[35] T. J. Davies , Savanna tree evolutionary ages inform the reconstruction of the paleoenvironment of our hominin ancestors. Sci. Rep. **10**, 12430 (2020). 32709951 10.1038/s41598-020-69378-0 PMC7381606

[36] A. S. L. Donkpegan, J.-L. Doucet, O. J. Hardy, M. Heuertz, R. Piñeiro, Miocene diversification in the savannahs precedes tetraploid rainforest radiation in the African tree genus *Afzelia* (Detarioideae, Fabaceae). Front. Plant Sci. **11**, 798 (2020). 32625223 10.3389/fpls.2020.00798 PMC7313659

[37] M. C. Veranso-Libalah, G. Kadereit, R. D. Stone, T. L. P. Couvreur, Multiple shifts to open habitats in Melastomateae (Melastomataceae) congruent with the increase of African Neogene climatic aridity. J. Biogeogr. **45**, 1420–1431 (2018).

[38] N. A. O’Mara , Constraining Plio-Pleistocene shifts in Northwest African hydroclimate, ecosystem distributions, and marine productivity: New paleo-records across the mid-Pleistocene transition. Paleoceanogr. Paleoclimatol. **39**, e2023PA004777 (2024).

[39] N. A. O’Mara , Pleistocene drivers of Northwest African hydroclimate and vegetation. Nat. Commun. **13**, 3552 (2022). 35729104 10.1038/s41467-022-31120-x PMC9213457

[40] T. D. Herbert , Late Miocene global cooling and the rise of modern ecosystems. Nat. Geosci. **9**, 843–847 (2016).

[41] M. Steinthorsdottir , The Miocene: The future of the past. Paleoceanogr. Paleoclimatol. **31**, e2020PA004037 (2020).

[42] C. E. R. Lehmann , Savanna vegetation-fire-climate relationships differ among continents. Science **343**, 548–552 (2014). 24482480 10.1126/science.1247355

[43] S. I. Higgins, S. Scheiter, Atmospheric CO_2_ forces abrupt vegetation shifts locally, but not globally. Nature **488**, 209–212 (2012). 22763447 10.1038/nature11238

[44] A. C. Staver, J. O. Abraham, G. P. Hempson, A. T. Karp, J. T. Faith, The past, present, and future of herbivore impacts on savanna vegetation. J. Ecol. **109**, 2804–2822 (2021).

[45] S. Archibald , Biological and geophysical feedbacks with fire in the Earth system. Environ. Res. Lett. **13**, 033003 (2018).

[46] E. Schefuß, L. M. Dupont, Multiple drivers of Miocene C_4_ ecosystem expansions. Nat. Geosci. **13**, 463–464 (2020).

[47] Z.-H. Tang, Z.-L. Ding, A palynological insight into the Miocene aridification in the Eurasian interior. Palaeoworld **22**, 77–85 (2013).

[48] B. J. Tipple, M. Pagani, The early origins of terrestrial C_4_ photosynthesis. Annu. Rev. Earth Planet. Sci. **35**, 435–461 (2007).

[49] W. J. Bond, What limits trees in C_4_ grasslands and savannas? Annu. Rev. Ecol. Evol. Syst. **39**, 641–659 (2008).

[50] W. J. Bond, Large parts of the world are brown or black: A different view on the “green world” hypothesis. J. Veg. Sci. **16**, 261–266 (2005).

[51] W. J. Bond, J. E. Keeley, Fire as a global “herbivore”: The ecology and evolution of flammable ecosystems. Trends Ecol. Evol. **20**, 387–394 (2005). 16701401 10.1016/j.tree.2005.04.025

[52] T. J. Gallaher , Grasses through space and time: An overview of the biogeographical and macroevolutionary history of *Poaceae*. J. Syst. Evol. **60**, 522–569 (2022).

[53] H. Zhou, B. R. Helliker, M. Huber, A. Dicks, E. Akcay, C_4_ photosynthesis and climate through the lens of optimality. Proc. Natl. Acad. Sci. U.S.A. **115**, 12057–12062 (2018). 30401739 10.1073/pnas.1718988115 PMC6255158

[54] I. Grass Phylogeny Working Group, New grass phylogeny resolves deep evolutionary relationships and discovers C_4_ origins. New Phytol. **193**, 304–312 (2012). 22115274 10.1111/j.1469-8137.2011.03972.x

[55] S. Bony , Robust direct effect of carbon dioxide on tropical circulation and regional precipitation. Nat. Geosci. **6**, 447–451 (2013).

[56] C. E. Lehmann, S. A. Archibald, W. A. Hoffmann, W. J. Bond, Deciphering the distribution of the savanna biome. New Phytol. **191**, 197–209 (2011). 21463328 10.1111/j.1469-8137.2011.03689.x

[57] J. C. Aleman , Floristic evidence for alternative biome states in tropical Africa. Proc. Natl. Acad. Sci. U.S.A. **117**, 28183–28190 (2020). 33109722 10.1073/pnas.2011515117 PMC7668043

[58] S. Archibald, G. P. Hempson, Competing consumers: Contrasting the patterns and impacts of fire and mammalian herbivory in Africa. Philos. Trans. R. Soc. B Biol. Sci. **371**, 20150309 (2016). 10.1098/rstb.2015.0309 PMC4978867 27502374

[59] A. J. Crocker , Astronomically controlled aridity in the Sahara since at least 11 Mya. Nat. Geosci. **15**, 671–676 (2022).

[60] R. R. Kuechler, L. M. Dupont, E. Schefuß, Hybrid insolation forcing of Pliocene monsoon dynamics in West Africa. Clim. Past **14**, 73–84 (2018).

[61] R. R. Kuechler, E. Schefuß, B. Beckmann, L. Dupont, G. Wefer, NW African hydrology and vegetation during the last glacial cycle reflected in plant-wax-specific hydrogen and carbon isotopes. Quat. Sci. Rev. **82**, 56–67 (2013).

[62] Y. Lancelot , “Site 368; Cape Verde rise” in Proceedings of the Initial Reports of the Deep Sea Drilling Project, J. Gardner, J. Herring, Eds. (Texas A&M University, Ocean Drilling Program, College Station, TX, 1978), **vol. 41**, pp. 233–326.

[63] W. Ruddiman , “Site 659” in Proceedings of the Ocean Drilling Program, Part A: Initial Reports (Texas A&M University, Ocean Drilling Program, College Station, TX, 1988), **Vol. 108**, pp. 221.

[64] R. Sayre , A New Map of Standardized Terrestrial Ecosystems of Africa (Association of American Geographers, Washington, DC, 2013), pp. 24.

[65] E. E. Bray, E. D. Evans, Distribution of n-paraffins as a clue to recognition of source beds. Geochim. Cosmochim. Acta **22**, 2–15 (1961).

[66] H. A. M. Ali, R. W. Mayes, B. L. Hector, A. K. Verma, E. R. ØRskov, The possible use of n-alkanes, long-chain fatty alcohols and long-chain fatty acids as markers in studies of the botanical composition of the diet of free-ranging herbivores. J. Agric. Sci. **143**, 85–95 (2005).

[67] T. Badewien, A. Vogts, L. Dupont, J. Rullkötter, Influence of late Pleistocene and Holocene climate on vegetation distributions in southwest Africa elucidated from sedimentary n-alkanes—Differences between 12°S and 20°S. Quat. Sci. Rev. **125**, 160–171 (2015).

[68] M. Griepentrog , Influence of plant growth form, habitat and season on leaf-wax n-alkane hydrogen-isotopic signatures in equatorial East Africa. Geochim. Cosmochim. Acta **263**, 122–139 (2019).

[69] A. Vogts, H. Moossen, F. Rommerskirchen, J. Rullkötter, Distribution patterns and stable carbon isotopic composition of alkanes and alkan-1-ols from plant waxes of African rain forest and savanna C_3_ species. Org. Geochem. **40**, 1037–1054 (2009).

[70] F. Rommerskirchen, A. Plader, G. Eglinton, Y. Chikaraishi, J. Rullkötter, Chemotaxonomic significance of distribution and stable carbon isotopic composition of long-chain alkanes and alkan-1-ols in C_4_ grass waxes. Org. Geochem. **37**, 1303–1332 (2006).

[71] Y. Garcin , Reconstructing C_3_ and C_4_ vegetation cover using n-alkane carbon isotope ratios in recent lake sediments from Cameroon, Western Central Africa. Geochim. Cosmochim. Acta **142**, 482–500 (2014).

[72] C. R. Magill, G. Eglinton, T. I. Eglinton, Isotopic variance among plant lipid homologues correlates with biodiversity patterns of their source communities. PLoS One **14**, e0212211 (2019). 30811453 10.1371/journal.pone.0212211 PMC6392421

[73] R. R. Tweedy , African C_3_ grass n-alkane distributions and plant chemotaxonomy. Org. Geochem. **208**, 104994 (2025).

[74] M. Bezabih, W. F. Pellikaan, A. Tolera, W. H. Hendriks, Evaluation of n-alkanes and their carbon isotope enrichments (δ^13^C) as diet composition markers. Animal **5**, 57–66 (2011). 22440702 10.1017/S1751731110001515

[75] R. S. Dodd, F. Fromard, Z. A. Rafii, F. Blasco, Biodiversity among West African *Rhizophora*: Foliar wax chemistry. Biochem. Syst. Ecol. **23**, 859–868 (1995).

[76] I. Kristen , Biomarker and stable carbon isotope analyses of sedimentary organic matter from Lake Tswaing: Evidence for deglacial wetness and early Holocene drought from South Africa. J. Paleolimnol. **44**, 143–160 (2010).

[77] M. Sonibare, M. Soladoye, Y. Ekine-Ogunlana, A chemotaxonomic approach to the alkane content of three species of *Anthocleista Afzel* (*Loganiaceae*). Afr. J. Biotechnol. **6**, 1516–1520 (2010).

[78] J. Gensel , Origin, transport, and retention of fluvial sedimentary organic matter in South Africa’s largest freshwater wetland, Mkhuze wetland system. Biogeosciences **19**, 2881–2902 (2022).

[79] G. Bocksberger , Climate and the distribution of grasses in West Africa. J. Veg. Sci. **27**, 306–317 (2016).

[80] V. F. Schwab , Effect of aridity on δ^13^C and δD values of C_3_ plant- and C_4_ graminoid-derived leaf wax lipids from soils along an environmental gradient in Cameroon (Western Central Africa). Org. Geochem. **78**, 99–109 (2015).

[81] Y. Garcin , Hydrogen isotope ratios of lacustrine sedimentary n-alkanes as proxies of tropical African hydrology: Insights from a calibration transect across Cameroon. Geochim. Cosmochim. Acta **79**, 106–126 (2012).

[82] D. Zhang , Carbon isotopic composition of plant waxes, bulk organics and carbonates from soils of the Serengeti grasslands. Geochim. Cosmochim. Acta **311**, 316–331 (2021).

[83] C. Häggi , Using multi-homolog plant-wax carbon isotope compositions to reconstruct tropical vegetation types. J. Geophy. Res. Biogeosci. **129**, e2023JG007946 (2024).

[84] N. A. O’Mara , Capturing vegetation gradients along the West African margin using terrestrial plant biomarkers in marine sediments. Geochem. Geophys. Geosys. **26**, e2025GC012274 (2025).

[85] M. H. Trauth, J. C. Larrasoaña, M. Mudelsee, Trends, rhythms and events in Plio-Pleistocene African climate. Quat. Sci. Rev. **28**, 399–411 (2009).

[86] J. E. Tierney, F. S. R. Pausata, P. B. deMenocal, Rainfall regimes of the Green Sahara. Sci. Adv. **3**, e1601503 (2017). 28116352 10.1126/sciadv.1601503 PMC5242556

[87] J. C. Larrasoaña, A. P. Roberts, E. J. Rohling, Dynamics of Green Sahara periods and their role in hominin evolution. PLoS One **8**, e76514 (2013). 24146882 10.1371/journal.pone.0076514 PMC3797788

[88] D. L. Fox , Stable isotope ecology of the early Miocene Rusinga Island mammalian communities from the Hiwegi and Kulu Formations (Nyanza Province, Western Kenya). Palaeogeogr. Palaeoclimatol. Palaeoecol. **679**, 113248 (2025).

[89] F. E. Oboh, Depositional history of the E2.0 reservoir in the Kolo Creek Field, Niger Delta. J. Pet. Geol. **16**, 197–212 (1993).

[90] P. J. Whybrow, H. A. McClure, Fossil mangrove roots and palaeoenvironments of the Miocene of the eastern Arabian Peninsula. Palaeogeogr. Palaeoclimatol. Palaeoecol. **32**, 213–225 (1980).

[91] A. Novello , Phytoliths indicate significant arboreal cover at Sahelanthropus type locality TM266 in northern Chad and a decrease in later sites. J. Hum. Evol. **106**, 66–83 (2017). 28434541 10.1016/j.jhevol.2017.01.009

[92] A. Zazzo , Herbivore paleodiet and paleoenvironmental changes in Chad during the Pliocene using stable isotope ratios of tooth enamel carbonate. Paleobiology **26**, 294–309 (2000).

[93] B. H. Passey , Strengthened East Asian summer monsoons during a period of high-latitude warmth? Isotopic evidence from Mio-Pliocene fossil mammals and soil carbonates from northern China. Earth Planet. Sci. Lett. **277**, 443–452 (2009).

[94] D. L. Fox, P. L. Koch, Carbon and oxygen isotopic variability in Neogene paleosol carbonates: Constraints on the evolution of the C_4_-grasslands of the Great Plains, USA. Palaeogeogr. Palaeoclimatol. Palaeoecol. **207**, 305–329 (2004).

[95] C. Latorre, J. Quade, W. C. McIntosh, The expansion of C_4_ grasses and global change in the late Miocene: Stable isotope evidence from the Americas. Earth Planet. Sci. Lett. **146**, 83–96 (1997).

[96] T. L. P. Couvreur , Tectonics, climate and the diversification of the tropical African terrestrial flora and fauna. Biol. Rev. **96**, 16–51 (2021). 32924323 10.1111/brv.12644 PMC7821006

[97] E. J. Edwards , The origins of C_4_ grasslands: Integrating evolutionary and ecosystem science. Science **328**, 587–591 (2010). 20431008 10.1126/science.1177216

[98] H. P. Linder, C. E. R. Lehmann, S. Archibald, C. P. Osborne, D. M. Richardson, Global grass (Poaceae) success underpinned by traits facilitating colonization, persistence and habitat transformation. Biol. Rev. **93**, 1125–1144 (2018). 29230921 10.1111/brv.12388

[99] D. D’Onofrio, J. von Hardenberg, M. Baudena, Not only trees: Grasses determine African tropical biome distributions via water limitation and fire. Glob. Ecol. Biogeogr. **27**, 714–725 (2018).

[100] W. A. Hoffmann , Ecological thresholds at the savanna-forest boundary: How plant traits, resources and fire govern the distribution of tropical biomes. Ecol. Lett. **15**, 759–768 (2012). 22554474 10.1111/j.1461-0248.2012.01789.x

[101] W. J. Bond, Open Ecosystems: Ecology and Evolution Beyond the Forest Edge (Oxford University Press, 2019), pp. 192.

[102] J. R. Ehleringer, T. E. Cerling, B. R. Helliker, C_4_ photosynthesis, atmospheric CO_2_, and climate. Oecologia **112**, 285–299 (1997). 28307475 10.1007/s004420050311

[103] M. Sankaran , Determinants of woody cover in African savannas. Nature **438**, 846–849 (2005). 16341012 10.1038/nature04070

[104] S. Scheiter , Fire and fire-adapted vegetation promoted C_4_ expansion in the late Miocene. New Phytol. **195**, 653–666 (2012). 22712748 10.1111/j.1469-8137.2012.04202.x

[105] K. Methner , Middle Miocene long-term continental temperature change in and out of pace with marine climate records. Sci. Rep. **10**, 7989 (2020). 32409728 10.1038/s41598-020-64743-5 PMC7224295

[106] X. Yang, J. Groeneveld, Z. Jian, S. Steinke, L. Giosan, Middle Miocene intensification of South Asian monsoonal rainfall. Paleoceanogr. Paleoclimatol. **35**, e2020PA003853 (2020).

[107] E. Wubben , Tropical warming and intensification of the West African monsoon during the Miocene Climatic Optimum. Paleoceanogr. Paleoclimatol. **39**, e2023PA004767 (2024).

[108] N. Werner, Z. Wang, L. Werdelin, Q. Zhang, East African uplift as a catalyst for Middle Miocene faunal transitions. Sci. Adv. **11**, eadx6569 (2025). 41091858 10.1126/sciadv.adx6569 PMC12524987

[109] J. Zhang , Modeling the effects of global cooling and the Tethyan Seaway closure on North African and South Asian climates during the Middle Miocene climate transition. Palaeogeogr. Palaeoclimatol. Palaeoecol. **619**, 111541 (2023).

[110] Z. Zhang , Aridification of the Sahara desert caused by Tethys sea shrinkage during the late Miocene. Nature **513**, 401–404 (2014). 25230661 10.1038/nature13705

[111] Z. Chen, Q. Wen, H. Yang, Impact of Tibetan Plateau on North African precipitation. Clim. Dyn. **57**, 2767–2777 (2021).

[112] CenCO_2_PIP Consortium, Toward a Cenozoic history of atmospheric CO_2_. Science **382**, 1136 (2023). 10.1126/science.adi5177 38060645

[113] L. M. Mejía , A diatom record of CO_2_ decline since the late Miocene. Earth Planet. Sci. Lett. **479**, 18–33 (2017).

[114] C. T. Bolton , Decrease in coccolithophore calcification and CO_2_ since the middle Miocene. Nat. Commun. **7**, 10284 (2016). 26762469 10.1038/ncomms10284 PMC4735581

[115] T. Tanner, I. Hernández-Almeida, A. J. Drury, J. Guitián, H. Stoll, Decreasing atmospheric CO_2_ during the late Miocene cooling. Paleoceanogr. Paleoclimatol. **35**, e2020PA003925 (2020).

[116] R. Gladstone, R. Flecker, P. Valdes, D. Lunt, P. Markwick, The Mediterranean hydrologic budget from a late Miocene global climate simulation. Palaeogeogr. Palaeoclimatol. Palaeoecol. **251**, 254–267 (2007).

[117] A. Micheels, J. Eronen, V. Mosbrugger, The late Miocene climate response to a modern Sahara Desert. Glob. Planet. Change **67**, 193–204 (2009).

[118] D. L. Griffin, Aridity and humidity: Two aspects of the late Miocene climate of North Africa and the Mediterranean. Palaeogeogr. Palaeoclimatol. Palaeoecol. **182**, 65–91 (2002).

[119] A. E. Holbourn , Late Miocene climate cooling and intensification of Southeast Asian winter monsoon. Nat. Commun. **9**, 1584 (2018). 29679005 10.1038/s41467-018-03950-1 PMC5910391

[120] P. Sanyal , Intensification of monsoon, microclimate and asynchronous C_4_ appearance: Isotopic evidence from the Indian Siwalik sediments. Palaeogeogr. Palaeoclimatol. Palaeoecol. **296**, 165–173 (2010).

[121] A. T. Karp, J. M. Russell, J. O. Abraham, T. Strydom, A. C. Staver, Fecal biomarkers in soils record landscape-scale wild herbivore abundance. Geochem. Geophys. Geosys. **26**, e2025GC012285 (2025).

[122] K. Prost, J. J. Birk, E. Lehndorff, R. Gerlach, W. Amelung, Steroid biomarkers revisited—Improved source identification of faecal remains in archaeological soil material. PLoS One **12**, e0164882 (2017). 28060808 10.1371/journal.pone.0164882 PMC5217961

[123] IPCC, “Climate change 2023: Synthesis report” in Contribution of working groups I, II and III to the Sixth Assessment Report of the Intergovernmental Panel on Climate Change, H. Lee, J. Romero, Eds. (IPCC, Geneva, Switzerland, 2023), pp. 35–115.

[124] S. E. Nicholson, The West African Sahel: A review of recent studies on the rainfall regime and its interannual variability. Int. Sch. Res. Not. **2013**, 453521 (2013).

[125] A. M. Jewell , Three North African dust source areas and their geochemical fingerprint. Earth Planet. Sci. Lett. **554**, 116645 (2021).

[126] J. Backman, I. Raffi, D. Rio, E. Fornaciari, H. Pälike, Biozonation and biochronology of Miocene through Pleistocene calcareous nannofossils from low and middle latitudes. Newslett. Stratigr. **45**, 221–244 (2012).

[127] I. Raffi, C. Agnini, J. Backman, R. Catanzariti, A Cenozoic calcareous nannofossil biozonation from low and middle latitudes: A synthesis. J. Nannoplankton Res. **36**, 121–132 (2016).

[128] P. J. Polissar, W. J. D’Andrea, Uncertainty in paleohydrologic reconstructions from molecular δD values. Geochim. Cosmochim. Acta **129**, 146–156 (2014).

[129] B. J. Tipple, S. R. Meyers, M. Pagani, Carbon isotope ratio of Cenozoic CO_2_: A comparative evaluation of available geochemical proxies. Paleoceanography **25**, PA3202 (2010).

[130] B. Boyle , The taxonomic name resolution service: An online tool for automated standardization of plant names. BMC Bioinformatics **14**, 16 (2013). 23324024 10.1186/1471-2105-14-16 PMC3554605

