# Supplementary material for: The origin and development of the Miocene northwest African savanna
Source: Proc Natl Acad Sci U S A. 2026 Aug 10;123(33):e2527340123. doi: 10.1073/pnas.2527340123 (PMC13486532; doi:10.1073/pnas.2527340123)
Supplement: Supplementary file 1 [file pnas.2527340123.sapp.pdf]

PNAS

Supporting Information for
The Origin and Development of the Miocene Northwest African Savanna

Anna K. Schartman[a]*; Pratigya J. Polissar[a]; Caroline A.E. Strömberg[b]

*Anna K. Schartman.
Email: aschartm@ucsc.edu

This PDF file includes:

Supporting text
Figures S1 to S17
Table S1
Dataset Legends S1 to S3
SI References

Other supporting materials for this manuscript include the following:

Datasets S1 to S3

Table of contents:

Supporting Information Text 2
S1: Study Site 2
S2: Age Models 3
S3: *n*-Alkane Extraction, Purification and Quantification 4
S4: *n*-Alkane $\delta^{13}C$ Determination 5
S5: Correction for Changing $\delta^{13}C$ of Atmospheric CO_2. 6
S6: *n*-Alkane Sourcing and Screening 6
S7: Modern Plant and Sediment *n*-Alkane Distribution Analysis 8
S8: Relationship of Middle Miocene Carbon Isotope Record to Modern Plant $\delta^{13}C$ 10
S9: Establishing the Timing of Open Ecosystem Expansion from %C_{33} Data 12
S10: Trends in Late Miocene $\delta^{13}C$ 13
S11: Establishing the Initial Age of C_4 Expansion 13
S12: Calculation and MDS Analysis of Late Miocene C_3 and C_4 Distributions 14
S13: Compilation and Analysis of Tropical and Subtropical C_4 Expansions 15
Supporting Figures 17
Supporting Tables 36
SI References 37

Supporting Information Text

S1: Study Site

ODP 659 (18°04 N, 21°01 W; 3,070 m water depth) and DSDP 368 (17°30 N, 21°21 W; 3,366 m water depth) are located ~ 500 – 525 km offshore Mauritania on the Cape Verde Plateau and Rise, respectively. Both sites lie within the Canary Current system today, and the region experiences high productivity in boreal summer (May – October) driven by seasonal upwelling dynamics (Lathuilière et al., 2008). This pattern of upwelling has likely been in place since at least the early Miocene (Wubben et al., 2024), and Neogene deposition at both core sites primarily consists of nannofossil ooze with varying (but usually minor) inputs of silt and clay. Core recovery at ODP 659 ends in the latest Oligocene. Early Miocene to Cretaceous sediments at DSDP 368 are classified as clays, claystones and shales. The Neogene lithogenic fraction is usually interpreted as aeolian in origin (dust deposits), with some evidence for distal fluvial inputs during times of increased wetness on the continent (Crocker et al., 2022; Tiedemann et al., 1994). While ODP 659 sits on the Cape Verde Plateau at an elevated position and contains little evidence for the influence of mass transport events, DSDP 368 on the rise contains several well-developed turbidite deposits. These deposits largely end in the middle Miocene, as the Cape Verde Plateau became elevated and the site was separated from the continental rise by the recently uplifted plateau. Samples were selected to avoid these deposits. Sedimentology and burial depths at both sites (< 300 m below sea floor for the latest Oligocene) suggest no thermal maturation of core material, and *n*-alkane distributions from samples are indicative of well-preserved plant waxes, making them appropriate for use as terrestrial plant biomarkers (Lancelot et al., 1978; Polissar et al., 2019; Ruddiman et al., 1988).

Wind patterns and rainfall in NW Africa are largely controlled by the path of the intertropical convergence zone (ITCZ) and its influence on the position and expression of the West African Monsoon. During boreal winter when the ITCZ is positioned south of the equator, the dry northeast trade winds blowing off the continent export dust to the core sites. When the ITCZ swings north during boreal summer, moisture is delivered to the NW African continent from the tropical Atlantic. At the same time dust is entrained in both the African Easterly Jet (a lower tropospheric feature with a stable core over the modern Sahel) and the Saharan Air Layer (a hot, dry, low-level feature extending thousands of kilometers across the Sahara and Sahel) and exported westward over the Atlantic Ocean (Nicholson, 2013; Prospero et al., 2021; Sarnthein et al., 1982).

Because of the aeolian depositional regime recorded at ODP 659 and DSDP 368, terrestrial sedimentary plant waxes must be deposited to the core sites in wind systems like the features just described (Crocker et al., 2022; Lancelot et al., 1978; Nicholson, 2013; Ruddiman et al., 1988). This is supported by numerous studies of *n*-alkane $\delta^{13}C$ from marine sediments along the margin of West Africa which find good correlation between nearby landscape C_4 composition and the carbon isotope ratios of plant waxes in sediments and dust (Huang et al., 2000; Niedermeyer et al., 2016; Rommerskirchen et al., 2003; Schefuß et al., 2003; Vogts et al., 2012). Further, O'Mara et al. (O'Mara et al., 2025) modeled the continental source regions for cores sites off the NW African margin and found that plant-wax biomarkers are largely transported zonally from the regions of the savanna and Sahel grasslands between ~10 and 25°N. Investigation into the geographical source of dust deposited to ODP 659 from 11 Ma to Recent using radiogenic

isotopes imply that the dust source largely remained unchanged and is derived from the central and western source regions (Crocker et al., 2022; Jewell et al., 2021). Altogether, these data suggest that plant-wax biomarkers are derived roughly zonally from the nearby continent, and this has likely been the case since at least the beginning of the late Miocene.

Numerous pollen analyses from ODP 659 and neighboring sites have been undertaken for the Plio-Pleistocene (Hooghiemstra et al., 2006; Leroy and Dupont, 1994; Leroy and Dupont, 1997; Vallé et al., 2014) to understand vegetation change on the continent, but none exist for the Miocene Epoch. We extensively explored obtaining pollen spores from both sites, but due to extremely low concentrations were unable to recover enough for assemblage counts for the late or middle Miocene, even using a method appropriate for deep sea sediments with high sediment volumes in order to obtain greater quantities of pollen grains (Carina Hoorn, pers. comm.).

S2: Age Models

ODP 659. Our age model and stratigraphy integrate those outlined in Crocker et al. (2022) for the late Miocene and younger and Polissar et al. (2019) for the late Oligocene to late Miocene (Fig. S1). In summary, Crocker et al. (2022) created a revised spliced composite section for the holes A – C by correlating XRF data and core images. They then produced the age model by tuning XRF geochemical measurements to the La2004 astronomical solution (Laskar et al., 2004) for summer insolation, guided by the benthic foraminifer oxygen isotope values measured at ODP 659 (Tiedemann et al., 1994) as well as the LR04 benthic oxygen isotope stack (Lisiecki and Raymo, 2005), and updated paleomagnetic, nannofossil and planktonic foraminiferal datums (Crocker et al., 2022 and references therein; Ruddiman et al., 1988). This age model extends to the base of holes B and C, after which the splice is entirely composed of hole A. To extend the age model into the middle and early Miocene through the sedimentary sequence of hole A, we used five nannofossil bio-horizons reported in the age model of Polissar et al. (2019) which occurred below the last tie point in the splice of Crocker et al. (2022). To move samples from the meters below seafloor scale (mbsf) to an updated meter composite depth scale (mcd) across three holes, the Crocker et al. (2022) splice was again integrated with that of Polissar et al. (2019). We used the former publication's composite depth scale for all samples above 185.4 mbsf (hole A) and 199.4 mbsf (hole B) with five additional depths from Polissar et al. (2019), which occurred deeper in the core. For hole C, the splice of Crocker et al. (2022) was used unchanged. Updating the splice did not affect the depths or ages of the nannofossil bio-horizons used as tie points in the integrated age model. Linear interpolations between tie points were used to calculate sample ages from the combined age models. To integrate our analysis with that of Polissar et al. (2019), we also recalculated the depth on the updated mcd scale and ages for all samples published in that study. In all figures, the Polissar et al. (2019) data is plotted using these updated ages. Finally, note that the last tie point occurs at 24.4 Ma at this site, and all sample ages older than this are extrapolated.

DSDP 368. Age control at this site is less well established that that of ODP 659 and to our knowledge no updates have been made to the original depth-age model published in the Initial Reports Volume for Leg 41 of the Deep-Sea Drilling Program (DSDP; Bukry, 1978; Čepek et al., 1978; Johnson, 1978; Lancelot et al., 1978). Only one hole was drilled at this site (hole Z) and coring was undertaken intermittently through the Plio-Pleistocene and continually beginning in the late Miocene through to the base of the sequence (Lancelot et al., 1978). Age control is

established by eight calcareous nannoplankton zonations and one well-constrained radiolarian bio-horizon as identified and described in the DSDP Initial Reports Volume (Bukry, 1978; Čepek et al., 1978; Johnson, 1978; Lancelot et al., 1978). To produce the most conservative estimate of the age-depth relationship for this site, we relied on averaged nannofossil zonation ages placed within the averaged depths of drilled sediment where those zones were identified. Because of the dearth of nannofossil preservation, limited fossil identification, and the way that the hole was spot-cored, depths within the core are not all assigned a fossil zonation making the identification of bio-horizons difficult. Instead, we took the top and bottom age for each nannofossil zone, updated following Backman et al. (2012) and Raffi et al. (2016), and the top and bottom depth of the drilled sediment where that zone was identified and averaged the nannofossil zonation ages and sediment depths to arrive at tie-points (Fig. S2). This allows us to constrain depth ranges (the cored sediment where the nannofossil zone was found) to age ranges (the entire zonation age). Because there are no nannofossil zones identified in the Initial Reports Volume in the early Miocene, we instead used the entire taxon range of *Discoaster druggi* to create a tie point at 20.27 ± 2.32 Ma. This taxon was identified in Hole A, Core 16, section 4 by Bukry (1978). Young (1998) lists the taxon range for *D. druggi* as spanning NN2–NN3. Therefore, we used the updated ages as listed in Backman et al. (2012) for NN2–NN3 (17.96 – 22.59 Ma) to calculate the average age for this tie-point. We elected to use this tie-point because preservation in the early Miocene in this core is extremely sparse and there is no other identified nannofossil zones after CNM7 ends at 15.73 Ma until CNE6 begins at 48.37 Ma. Further, we also included the well-constrained radiolarian bio-horizon (*C. costata* first occurrence) at 17.69 Ma identified between 250.57 – 256.00 mbsf as an additional tie point for the early Miocene, which agrees well with the linear interpolation between nannoplankton zones (Nigrini et al., 2006). Next, we calculated sample ages by the linear interpolation between these 9 tie points. That this age model is a good approximation to the age-depth relationship is established by its agreement to both the *n*-alkane distribution and $\delta^{13}C$ curves at the much better constrained site of ODP 659 (Fig. S3 and S4). Splice and age model information for both sites is presented in the SI Appendix, Dataset S1.

S3: *n*-Alkane Extraction, Purification and Quantification

Plant waxes were extracted from freeze dried and homogenized sediments (average mass ~77 g) using a Dionex Accelerated Solvent Extractor (ASE-350) in 9:1 v/v dichloromethane:methanol. 100 µl of an internal recovery and quantification standard (~20 ng/µl each of 5α-androstane, 1-1'binapthyl, stearyl stearate, 11-eicosenol, 11-eicosenoic acid) was added to the total lipid extract for each sample to determine molecular concentrations by gas chromatography mass spectrometry (GC-MS). Samples were left to react with solvent-cleaned copper pellets for between 24 hours and 2 weeks in order to remove any elemental sulfur which can interfere with quantification and compound-specific $\delta^{13}C$ and $\delta^{2}H$ measurements. The total lipid extracts were evaporated under a steady stream of N_2 and brought up in 2:1 dichloromethane:isopropanol. They were then loaded onto 0.5 g aminopropyl (LC-NH2) columns and separated into neutral, acid and polar fractions with 4 ml each of 2:1 dichloromethane:isopropanol, 4% acetic acid in diethyl ether and methanol, respectively. Neutral fractions were then dried down under N_2 and brought up in 0.5 µl of hexane for silica gel column chromatography (following O'Mara et al., 2024) in order to separate the neutral fraction into an aliphatic, aromatic/ketone/ester and polar fractions. Plant wax *n*-alkane concentrations (C_{13} – C_{36}) were determined by GC-MS on an Agilent 7890A GC interfaced to an Agilent 5975B MS running in full scan mode and equipped

with a DB-5 silica capillary column (30.0m; 0.250 mm; 0.25μm). Quantification of target *n*-alkanes was completed by comparison of the m/z 57 ion peak area to that of an external authentic standard (Supleco, $C_8 – C_{40}$ Alkanes Calibration Standard, product #40147-U) run every 5 to 10 samples, relative to the m/z 245 ion peak area of the 5α-androstane added to the samples and C_8-C_{40} *n*-alkane standard mix. Sample replicates (duplicate – quadruplicate analyses) were run over a two-year period and the results of all analyses are reported in SI Appendix, Dataset S2. In our analysis we used several derived distribution metrics: $\%C_{31}$, $\%C_{33}$, $\%C_{35}$, $CPI_{(24-26)}$, and the relative abundances of C_{27}, C_{29}, C_{31} and C_{33} to the sum of those four chain lengths. We calculated the mean value of these metrics using the replicate data where it existed and estimated precision on each metric following methods for pooling replicate standard deviations adapted from Polissar and D'Andrea (2014). The 1σ standard deviation of $\%C_n$ across all sample replicates was always < 0.02, with an average value of 0.005, indicating excellent reproducibility. Results and error estimates are reported in the SI Appendix, Dataset S3. Distribution metrics ($\%C_n$) are displayed in Fig. S3.

After quantification by GC-MS, samples that contained large unresolved complex mixtures (UCMs) were purified using urea adduction before carbon isotope determination. Briefly, 300μl each of 10% urea in methanol (w/w), acetone and hexane were added to the dried aliphatic fraction, and this mixture was homogenized and frozen. Urea crystals, containing the long chain ($>C_{16}$) *n*-alkanes trapped as clathrates within the crystal structure, were washed repeatedly using hexane to remove all branched-cyclic compounds comprising the UCM. Urea crystals were then dissolved with 500μl each of organics-free de-ionized water and methanol, and liquid-liquid extraction was used to isolate *n*-alkanes in hexane. This process was repeated 2 to 3 times and samples were qualitatively re-assessed by GC-MS to ensure the complete removal of the UCM prior to isotope ratio analysis.

S4: *n*-Alkane $\delta^{13}C$ Determination

Sample *n*-alkane carbon isotope ratios were measured on a Thermo Scientific Delta V advantage coupled through a ConFlo IV to a Thermo Scientific Trace GC Ultra and Isolink. Samples were diluted in hexane and injected splitless into a PTV inlet held at 60°C and then ramped at 14.5°C/s to 320°C for transfer onto a Thermo Scientific TraceGOLD TG-5MS column (30.0m; 0.230mm; 0.25μm) at constant flow (1ml/min) of helium gas. The GC oven's initial temperature of 60°C was held for 1.5 minutes before ramping to 150°C (at 15°C/min) and to 320°C (at 4°C/min) with a final 10 minute hold. The combustion interface for $\delta^{13}C$ measurements was based upon the design and testing reported in Merritt et al. (1995). Sample molecules were combusted to CO_2 in a custom-built reactor consisting of three wires (nickel, copper, platinum) wound together and inserted inside a fused alumina tube. The reactor was fully oxidized with 100% O_2 gas initially, and a continuous bleed of 1% O_2 in helium was fed through the reactor at 0.05 ml/min using a mass-flow controller during each sample run to maintain the reactor's oxidation efficiency and ensure the complete combustion of sample carbon (Merritt et al., 1995). A standard containing *n*-alkanes of known isotopic composition (Mix A7, purchased from Arndt Schimmelmann, University of Indiana) was run every 2 – 5 samples to assess instrument performance and convert isotope values to the Vienna Pee Dee Belemnite (VPDB) scale. Samples were run in duplicate to triplicate spaced across the time frame of analysis and measurement precision was calculated using the methods of Polissar and D'Andrea (2014). The pooled 1σ standard error of the mean across all samples is ± 0.27, 0.20, 0.10, 0.09, 0.12 and

0.26‰ for C_{25}, C_{27}, C_{29}, C_{31}, C_{33} and C_{35}, respectively. This uncertainty includes the analytical uncertainty of our measurements and the uncertainty in assigning values on the VPDB scale. Carbon isotope measurements for chain lengths C_{25} – C_{35} (odd) are displayed in Fig. S4.

S5: Correction for Changing $\delta^{13}C$ of Atmospheric CO_2.

Plant wax *n*-alkane $\delta^{13}C$ can be affected by the $\delta^{13}C$ of the carbon source, atmospheric CO_2, which can potentially confound interpretation of their values. Therefore, we corrected all measured C_{25} – C_{35} carbon isotope ratios for changes in the $\delta^{13}C$ of atmospheric CO_2 through the Miocene using the benthic foraminifera-based estimates from Tipple et al. (2010) and normalized all results to the pre-industrial value of CO_2 (–6.5‰) following methods outlined in Polissar et al. (2019) and Polissar et al. (2021). Uncorrected and corrected $\delta^{13}C$ values, as well as the values used for each sample atmospheric $\delta^{13}C$, are reported in the SI Appendix, Dataset S3.

S6: *n*-Alkane Sourcing and Screening

Before *n*-alkane distributions can be used to understand historical vegetation dynamics, information must be obtained regarding the purity of the plant-wax signal. This is because thermal maturation, microbial activity, and petroleum contamination can alter a sediment's *n*-alkane profile, observable in a tendency towards equilibration between odd and even chain length abundances (Bray and Evans, 1961; Eglinton and Hamilton, 1967; Marzi et al., 1993). This relationship is often quantified using the Carbon Preference Index (CPI), a ratio of odd- to even-numbered *n*-alkanes in a sediment sample, as in Eq. S1:

(Eq. S1)
$$CPI_{(24\text{-}36)} = \frac{\Sigma_{odd}(C_{25-35})}{0.5C_{24} + \Sigma_{even}(C_{26-34}) + 0.5C_{36}}$$

where C_n is the concentration of that *n*-alkane. A value close to one is considered an indication of the overprinting of the plant-wax signal by non-plant-wax sources or alteration due to thermal maturation. NW African samples all display $CPI_{(24\text{-}36)}$ values above one (mean = 4.9, σ = 0.9, Fig. S5 and S6) which indicates that non-plant wax sources are not major contributors to the long-chain *n*-alkane reservoir for the majority of the record. We used CPI to screen samples and exclude those (CPI < 3.0) we thought were possibly not representative of a primary plant-wax signal. While CPI is an inherently qualitative metric, it is useful for providing information on possible alterations of *n*-alkanes distributions and isotopic content, and has been widely applied in this manner (Chevalier et al., 2015; Polissar et al., 2021). It is likely the degree to which alteration processes like petrogenic contamination, thermal maturation, and microbial activity affect the distribution of odd, long-chain compounds and their carbon isotopic values forms a continuum in our samples. The vast majority of samples are very little affected, which is substantiated by their high CPI values, carbon isotope signatures consistent with C_3 and C_4 vegetation, and terrestrial plant-wax distribution profiles. However, at some point samples become more affected, and their utility as paleo-environmental or climate indicators declines. CPI is a convenient way to capture the severity of this attenuation of the plant-wax signal, and while a cut-off value of 3.0 is inherently qualitative, those samples that fall below it tend to exhibit deviations from expected values for *n*-alkane $\delta^{13}C$ and distribution profiles in some fashion. For example, three samples we excluded all have $\%C_{35}$ values which are much higher than reasonable for a sedimentary plant wax, higher than all other values in the record (Fig. S3). Two others have anomalously low $\delta^{13}C$ measurements in more than one chain length (Fig. S4).

Additionally, these samples had unresolved complex mixtures, another indication of a petrogenic source (Liebezeit and Wöstmann, 2009). A cut off of CPI = 3.0 neatly separates these potentially problematic samples from the ones that are less altered.

Finally, we also note that our results are invariant to the inclusion or exclusion of those sample with CPI < 3.0. For example, when comparing the %C_{33} mean of all samples (including the ones we initially screened out) older than 16 Ma with those younger than 14 Ma using a two-tailed student t-test (the same test reported in our results for *n*-alkane distributions in the main text), the *p*-value is still robustly statistically significant (p=7.51x10^{-42}). Nor does it change the results of the running t-tests on %C_{33} (SI section S9) used to evaluate timing of initial landscape opening. Finally, there is also no effect on the carbon isotope record of the late Miocene as these six samples we excluded are middle Miocene and older. Therefore, although including these samples does not affect our interpretations or conclusions, we think there is sufficient reason to exclude them since they are not useful for providing information on past terrestrial ecosystems.

To evaluate the effect of non-plant-wax contributions on individual *n*-alkane compounds, we additionally computed the individual CPI (CPI_{indiv}) for odd and even long-chain *n*-alkanes (after Polissar et al., 2021):

(Eq. S2) $$\mathrm{CPI_{indiv}} = \frac{Cn}{0.5(C_{n-1} + C_{n+1})}$$

Like CPI, values of CPI_{indiv} are indicative of the potential for mixing between plant-wax and non-plant-wax sources, but focus on an individual homologue as opposed to the overall *n*-alkane distribution. Samples with lower values, and more mixing, are therefore of less use as a vegetation proxy. In samples from NW Africa, C_{25} and C_{27} are tightly clustered around low CPI_{indiv} means (1.7, 2.4, respectively) compared with *n*-C_{29} and above (C_{29} = 4.7, C_{31} = 8.1, C_{33} =8.2, C_{35} = 6.4; Fig. S7A). This could be because both *n*-C_{25} and *n*-C_{27} are exhibiting a greater influence from non-plant-wax sources compared to higher chain lengths, or because plants themselves produce more equal amounts of even and odd homologues below C_{28}. That the latter, plant-source cause, is the case is substantiated by a recent compilation of African plant *n*-alkane abundance measurements. These data show that modern east African plants have distributions of CPI_{indiv} values for C_{25} and C_{27} that are markedly lower than the four next higher odd chain lengths (Tweedy et al., 2025; Fig. S7B). We focus on odd chain lengths C_{29} – C_{33} in our discussion because (1) these are the most abundant in our samples and therefore least likely to be affected by minor contributions from other sources and (2) previous publications of modern plants and sediments as well as reconstructions using sedimentary *n*-alkanes for the region report these chain lengths. We include analysis of C_{35} as well because it has been used as an indicator of grasses in past publications (Polissar et al., 2019; Tweedy et al., 2025) and largely tracks C_{33} in our samples. Finally, our multidimensional scaling analysis outlined in the SI Appendix, section S12, includes C_{27} for completeness because (1) it occurs in fairly high abundance in our samples and as described above could be reflecting plant waxes, (2) all previous studies we use in that analysis published values for this chain length (O'Mara et al., 2024; Polissar et al., 2019) and (3) the main results of the multidimensional scaling analysis are invariant to its inclusion or exclusion.

S7: Modern Plant and Sediment *n*-Alkane Distribution Analysis

To evaluate the use of sedimentary *n*-alkane distributions as a proxy for ecosystem structure, we analyzed published *n*-alkane concentration data of modern African plants, sediments and soils, as well as Pleistocene NW African sediments. In these studies, knowledge of plants and ecosystem is constrained, allowing us to explore the relationships between *n*-alkane distributions and African plant growth form or ecosystem structure. We first compiled modern African plant *n*-alkane concentration data from the published literature for statistical analysis of chain length distributions (Ali et al., 2005; Badewien et al., 2015; Bezabih et al., 2011; Dodd et al., 1995; Garcin et al., 2014; Gensel et al., 2022; Griepentrog et al., 2019; Kristen et al., 2010; Magill et al., 2019; Rommerskirchen et al., 2006; Sonibare et al., 2010; Tweedy et al., 2025; Vogts et al., 2009). We kept all entries for which the sample was determined to a family level only (118 entries), genus level only (173 entries), or species level and below (676 entries), and cross referenced all listed taxa against the Taxonomic Name Resolution Service (TNRS) online database (Boyle et al., 2013) in order to standardize and identify all plant samples. If no suitable match could be found in this database (match score of less than 0.53) the samples were removed from our analysis (n = 18). This data curation resulted in 967 *n*-alkane distribution entries. Next, we calculated the $\%C_n$ metrics for each entry. To avoid biasing our analysis to plant species which had numerous entries, we averaged the relative distributions by species. For entries identified to the genus level or above, we did not perform any averaging, excepting those from Griepentrog et al. (2019) which the authors stated were collected from the same plant species. We focused on those growth forms for which there were more than 15 independent entries (includes tree, shrub, forb, grass, sedge, vine, succulent). Our final compilation includes 616 different taxa, each with $\%C_n$ information. Boxplots of the $\%C_n$ metrics by growth form are shown in Fig. S8. These plots support the use of these metrics to identify woody (tree and shrub) vs. herbaceous vegetation (especially grasses), as the measures of center for trees and shrubs are reliably lower compared to grasses (C_3 and C_4), particularly in $\%C_{33}$ and $\%C_{35}$.

To determine if modern African plant growth forms exhibit statistically different distributions of $\%C_n$, we used two-sample Kolmogorov-Smirnov tests to pairwise compare $\%C_n$ values of growth forms (tree, shrub, grass and forb). This non-parametric test evaluates whether the empirical distribution functions of two samples could be derived from the same probability distribution. Rejecting the null hypothesis at a given significance level suggests that the two sample distributions are not from the same underlying probability distribution; while failing to reject the null hypothesis suggests they may be from the same underlying distribution. Results are displayed in Fig. S9 and Table S1. In the table we have bolded all *p*-values which fall below the 1% confidence level when comparing two growth forms against each other. These tests suggests that the *n*-alkane distribution of modern African woody vegetation (trees and shrubs) is statistically different from that of grasses, supporting our use of $\%C_n$ to evaluate ecosystem structure. Conversely, trees and shrubs have much higher *p* values (especially for $\%C_{33}$ and $\%C_{35}$), implying that trees and shrubs may produce similar *n*-alkane distributions and are difficult to separate based on $\%C_n$.

The *n*-alkane distribution differences found in modern plants translate into modern tropical sediments: *n*-alkanes sourced from soils and lake sediments of open, grass-rich ecosystems tend to have higher values of $\%C_{31}$, $\%C_{33}$ and $\%C_{35}$ compared with those of forests and woodlands (Garcin et al., 2014; Garcin et al., 2012; Häggi et al., 2024; Schwab et al., 2015; Zhang et al.,

2021; Fig. S10). For example, soils taken from the top of the soil column from Serengeti grasslands (with grassy cover > 90%) have $\%C_{33}$ values that are reliably higher (mean and 1σ standard deviation= 0.68±0.07) than those from Serengeti open ecosystems containing more trees and shrubs (mean = 0.47±0.02; Zhang et al., 2021). Similarly, in Cameroon lake and soil sediments, dry savannas display more positive values ($\%C_{33}$ mean = 0.56±0.08) compared with transitional (treed savannas) or forested ecosystems ($\%C_{33}$ mean = 0.26±11) (Garcin et al., 2014; Garcin et al., 2012; Schwab et al., 2015). These patterns are also evident in South American soil samples spanning closed rainforests ($\%C_{33}$ mean = 0.24±0.09) to open savannas ($\%C_{33}$ mean =0.45±0.13), such as the Campo Cerrado, Campo Sujo/Limpo, and Llanos savanna (Häggi et al., 2024), suggesting these *n*-alkane chain length patterns may be a broad feature of woody and herbaceous tropical/subtropical ecosystems, despite major differences in species composition and evolutionary history.

That *n*-alkane metrics are a reliable proxy for shifts in ecosystem structure is supported by a recent study investigating *n*-alkane distributions from a suite of core-top sediments spanning the west African margin. Sites that received *n*-alkane deposition from grassland and savanna ecosystems both N and S of the equator (as modeled by atmospheric back trajectories of air parcels) exhibited higher values compared to those sites were *n*-alkanes were primarily sourced from the more tropical forested ecosystems. For example, sites that occur from 10°N – 30°N and 10°S – 30°S have mean $\%C_{33}$ values of 0.38±0.02 and 0.48±0.09 respectively, while sites between 10°N and 10°S have mean $\%C_{33}$ values of 0.33±0.04 (O’Mara et al., 2025).

The relationships between ecosystem structure and long-chain *n*-alkane distributions are found in Pleistocene sediments offshore NW Africa as well, supporting their use as a vegetation proxy (O’Mara et al., 2022). Between 520 and 360 ka, the NW African savanna underwent changes in distribution (shifts in latitude) and in composition (greening events) associated with atmospheric pCO_2 and orbital cyclicity. Changes in the C_4 grass component of the landscape as inferred from *n*-alkane $\delta^{13}C_{31}$ are well correlated with $\%C_{33}$ (Pearson’s r = 0.8). This is striking given that this historical ecosystem was likely always a savanna ($\%C_4$ inferred to have changed by a maximum of ~16% over the entire interval), suggesting that *n*-alkane distributions have the capability to track small changes in canopy cover under certain circumstances (c.f. O’Mara et al., 2024). Thus, the above evidence supports the use of these plant wax distribution metrics to identify shifts from open ecosystems (containing high abundances of herbaceous vegetation, particularly grasses) to a closed ecosystem (containing high abundances of woody vegetation) and *vice versa*.

Our new record uses *n*-alkane distributions to document a major ecosystem reorganization during the Miocene Climate Optimum and Miocene Climate Transition in NW Africa. Based on modern calibrations, we interpret this result as a switch from ecosystems dominated by woody vegetation to those that contain high abundances of grasses. To conclude that these middle Miocene ecosystems with high $\%C_{33}$ values are not composed of high abundances of herbaceous vegetation (grasses, but probably other forbs as well) would necessitate that either (1) *n*-alkane distribution patterns in plant lineages are not consistent through time or (2) these ecosystems are composed dominantly of the few trees and shrubs (or their ancestors) with very high $\%C_{33}$ values. While both scenarios are possible, they are not the most parsimonious explanations and are inconsistent with the other paleorecords outlined in the main text. Further, the continuity of distributions in plant lineages is supported by the increases in $\delta^{13}C$ of C_{35} and C_{33} prior to C_{31}

and C_{29} in our late Miocene record (the expected scenario based on C_4 grass distributions), suggesting that distinct distribution patterns have been a characteristic feature of plant lineages since at least 11 myr ago. There is currently no evidence from the literature for woodlands/shrublands with such high %C_{33} values in Africa, and therefore the second point, while possible, is not well supported.

We note that modern African succulents from the Succulent Karoo and the Fynbos biomes can have very high %C_n values (Fig. S8), but we find it unlikely that Miocene ecosystems were composed of plants from these lineages given that the evolution and radiation of succulents is generally considered a feature of the late Miocene (Arakaki et al., 2011), and floral records from Africa do not suggest high abundances of succulents. Likewise, we also think it unlikely that succulents or other plants using Crassulacean Acid Metabolism (CAM) were important part of the vegetation assemblages of NW during the middle or late Miocene because (1) the $\delta^{13}C$ record of the middle Miocene is inconsistent with high abundances of CAM photosynthesizers which have variable $\delta^{13}C$ values intermediate between C_3 and C_4, and (2) regional pollen records from the late Miocene do not record high abundances of pollen from lineages associated with CAM (e.g., Bonnefille, 2010; Feakins et al., 2013; Hoetzel et al., 2015).

Finally, while we interpret our record of high %C_{33} post 15 Ma as indicative of increasing C_3 and later C_4 grasses, we cannot exclude the possibility that some of this signal is due to the presence of C_3 and C_4 forbs on the landscape. In the main, forbs exhibit intermediate %C_{33} values between grasses on the one hand, and trees and shrubs on the other (Figs. S8 and S9). Pollen assemblage counts from regional records of late Miocene – Recent eastern Africa and Pliocene – Recent NW Africa show large quantities of Amaranthaceae, a family of forb and shrub well-adapted to arid and halophytic conditions (Bonnefille, 2010; Feakins et al., 2013; Hooghiemstra et al., 2006; Leroy and Dupont, 1994; Leroy and Dupont, 1997). This family is known to contain C_4 and C_3 species and is also present in deserts of North Africa today (Kherraze et al., 2018; Sage et al., 2007). Unfortunately, there are very few instances of this family that have been measured for either their carbon isotopic content or distributional profiles, and so it is unclear the degree to which our *n*-alkane biomarker record is consistent with this lineage's presence on the landscape of NW Africa. For example, our modern plant *n*-alkane database contains only 14 instances of this family, with a median value for %C_{33} and %C_{35} of 0.36 and 0.02, respectively. These values are intermediate between those of grasses and shrubs / trees, so from this limited data, it seems possible these plants could be contributing to the sedimentary archive, they can't explain the high values we observe post 15 Ma. However, more work investigating modern plant distributions and carbon isotope content would help elucidate this point. In summary, given our current understanding of the modern African plant distribution data as well as the Miocene vegetation records, we conclude that the simplest explanation for the change we see in *n*-alkane distributions is the proliferation of C_3, grass-rich savannas in the middle Miocene.

S8: Relationship of Middle Miocene Carbon Isotope Record to Modern Plant $\delta^{13}C$

The carbon isotopic composition of C_3 plant tissue is related to climate, most notably moisture availability but also temperature, humidity, light intensity, and pCO_2 (Diefendorf and Freimuth, 2017). In the modern, *n*-alkane $\delta^{13}C$ of C_3 plants shows a strong negative relationship with mean annual precipitation, with increasingly positive values under increasingly arid conditions. However, even in the same ecosystem, plant-wax $\delta^{13}C$ of modern plants can vary substantially,

perhaps due to phylogenetic and functional differences (Diefendorf and Freimuth, 2017 and references therein). How this variation will be integrated into the sedimentary record over geologic time is unclear. Our Miocene record of plant-wax $\delta^{13}C$ demonstrates no statistically significant differences before and after the timing of landscape opening in the middle Miocene at ~16 – 14 Ma (Fig. 3). For example, comparing the C_{29}, C_{31}, and C_{33} $\delta^{13}C$ values from the two-million-year intervals before and after this transition reveals no evidence for $\delta^{13}C$ change from a tropical / subtropical closed ecosystem to an open one (two-tailed t-tests, *p*-values > 0.05).

Whether or not this finding is unexpected is difficult to determine from modern data. There are overall few measurements of C_3 grasses from the tropics and subtropics and none that occur in open ecosystems (as opposed to forests) which would be the closest analog to the grasses that existed in the inferred open ecosystems of the Miocene. Vogts et al. (2009) measured modern plant *n*-alkane $\delta^{13}C$ from savanna ecosystems and found that C_3 savanna herbs are on average ~2‰ more negative compared to C_3 savanna trees (e.g., mean C_{31} of savanna trees and herbs = –33.7 and –35.7‰, respectively). Under a simple two endmember mixing model between trees and herbs, and assuming the mean values of C_{31} for both growth forms are a good approximation of the mean woody and herbaceous component of the landscape through the middle Miocene, an ecosystem that converted from 90% trees and 10% grasses to 10% trees and 90% grasses would have a $\delta^{13}C$ decrease of 1.5‰. However, this is the extreme case, and ecosystems composed of more trees would display a more muted $\delta^{13}C$ change (e.g., converting to a 60% grass and 40% tree ecosystem results in a negative shift of only 1‰). All the same, even a 1‰ change in $\delta^{13}C$ should be observable in our Miocene record as our analytical uncertainty is well below this (1σ standard error of the mean ≤ 0.27), if these values for savanna trees and herbaceous vegetation are good approximations for the differences in Miocene vegetation $\delta^{13}C$. With such a limited dataset (no C_3 grasses, C_3 savanna herbs = only 8 samples), it is quite possible that this is not the case.

Global compilations of plant-wax $\delta^{13}C$ suggest less variation between woody and herbaceous vegetation overall. For example, the mean difference between *n*-alkane $\delta^{13}C$ of trees and grasses from data compiled in Diefendorf and Freimuth (2017) is small, ~1‰. This finding is replicated in (Liu et al., 2022) when comparing median values of dicots and monocots (both monocots and grasses are more positive than dicots and trees in these two compilations). When comparing angiosperm woody vegetation (trees and shrubs) to herbaceous vegetation (graminoids and forbs) from (Diefendorf and Freimuth, 2017), this difference in mean $\delta^{13}C$ between growth forms is still small, with woody vegetation having mean $\delta^{13}C$ values of –34.9, –35.3, and –35.7‰ for C_{29}, C_{31} and C_{33}, respectively, while herbaceous vegetation has values of –35.0, –35.6 and –35.7‰. We note, however, that this compilation also contains very few measurements of C_3 grasses (8 only) and only one of these is from the PACMAD clade, which is the most likely clade of C_3 grasses to have existed in middle Miocene open ecosystems.

Because of the lack of (subtropical and tropical) C_3 grasses in modern *n*-alkane $\delta^{13}C$ compilations, we also investigated the differences due to growth form in modern plant bulk tissue $\delta^{13}C$. To do this, we first used the Taxonomic Name Resolution Service (Boyle et al., 2013) to identify the family, genus, and species of each entry in the leaf carbon isotope compilation of Cornwell et al. (2018).We then requested the trait information for plant growth

form from TRY, a global database of curated plant traits, for each species identified (Kattge et al., 2020). We condensed the output into six growth forms of interest: trees, shrubs, shrubs/trees (for those plants which span both growth form categories), forbs (including all herbs), graminoids, and grasses and matched these growth forms to the data in the carbon isotope compilation. The graminoid growth form includes all graminoids excepting the grasses, which are included as a separate growth form category. Entries with ambiguous growth forms or for which TNRS could not find any matches (match < 0.53) were excluded. This resulted in a total of 2,546 C_3 leaf carbon isotope measurements. Unfortunately, sampling density of different growth forms is highly variable, with 1,546 from trees and only 82 from grasses. Further, most grasses appear to have been collected in temperate regions with only 20 occurring in the tropics or subtropics (40°S – 40°N). Therefore, we opted to investigate the differences between woody (trees, shrubs, and shrubs/trees) and herbaceous (forbs, graminoids, grasses) growth forms. Comparing the distributions of woody to herbaceous growth forms from this dataset shows a statistically significant difference of ~1‰ between distribution means (t-test, $p = 2.34\times10^{-18}$; Fig. S11). However, when narrowing the region of interest to the drier regions of the tropics and subtropics which contain ecosystems that we think best match those of Miocene NW Africa (10 – 40°N and 10 – 40°S), the difference in means becomes statistically insignificant ($p = 0.83$) while sample density of both distributions is still high (n = 1,144 and 218 for woody and herbaceous growth forms, respectively; Fig. S11).

Overall, the modern data is inconclusive regarding whether we would expect to see a carbon isotopic shift in our middle Miocene $\delta^{13}C$ record when we infer an ecosystem change from a closed C_3 woody vegetation assemblage to an open C_3 grass-rich vegetation assemblage. It is possible that over geologic time, plant-wax $\delta^{13}C$ values are controlled more strongly by variables other than growth form and phylogeny, such as soils or precipitation (Cornwell et al., 2018; Diefendorf and Freimuth, 2017; Kohn, 2010). More data from modern subtropical and tropical C_3 grasses, for example from species measured by Tweedy et al. (2025) for *n*-alkane distributions, would be useful to investigate this further.

S9: Establishing the Timing of Open Ecosystem Expansion from %C_{33} Data

To constrain the timing of ecosystem opening in NW Africa in the middle Miocene we applied a series of running student t-tests to our %C_{33} record. While %C_{33} values clearly separate into two major groups through time in our record (SI Appendix, Fig. S12), the beginning age of the transition between the two groups is not readily apparent from visual inspection. We first identified an early Miocene interval of 23 – 17 Ma with consistent %C_{33} values to test our middle Miocene record against. We then segmented our record younger than 17 Ma into 1 million-year bins, such that the initial bin had a starting age of the sample closest to 17 Ma (16.7 Ma). We used Levene's test to determine if the variances between each 1 myr distribution and the 23 – 17 Ma reference interval could be taken as different at the 5% confidence interval. Next, we calculated the test statistics and *p*-values of the two-sample t-tests between the 1 myr bins and reference interval either assuming equal variances or unequal variances depending on the results of the Levene test. We then iterated this procedure 13 more times, updating the initial bin's start age to the next youngest sample to evaluate performance using different clusters of sample data. We found that when a 1 myr bin is composed of more than four samples and all from < 15.0 Ma, *p*-values are consistently much lower than 0.05, and therefore can be said to be significantly different from the early Miocene reference (Fig. S13A). When a bin contains samples that are >

16.0 Ma, *p*-values are either above 0.05 or very close to 0.05, making it difficult to determine if the distributions are truly different. We take 16 Ma as the approximate earliest time of landscape opening based on the results of this test but note that a higher resolution dataset that does not contain a gap between ~15.0 and ~15.5 Ma would be better positioned to pinpoint this time more exactly. Finally, we repeated this test using a reference range that included Oligocene samples to test if our results were affected by the choice of reference range, but resultant *p*-values for each 1-million-year bin, and their trend through time, were very similar (Fig. S13B).

S10: Trends in Late Miocene $\delta^{13}C$

We used the first and second derivative of a locally weighted scatterplot smoothing (lowess) curve fit to the $\delta^{13}C$ data of each plant-wax homolog to identify periods of faster and slower change within the late Miocene (Figs. S14 and S15). We employ this method because it is a simple, but objective approach to identifying periods of change with the carbon isotope record that are also visible to the eye. First, we implemented the lowess fit to the combined Polissar et al. (2019) and new carbon isotope data using the python package *statsmodel.api*. We had the model estimate each y value of the lowess fit using 20% of the data from 15 to 5 Ma (frac = 0.2) which amounted to a window of 25 data points for each y value calculation. Next, we estimated the first and second derivative of the output array with NumPy's gradient function. We fit a final lowess curve to the second derivative (frac = 0.15) to reduce the effect of outliers that made the trends in the second derivative difficult to observe. Minimum and maximum points within the second derivative correspond to locations where the rate of change is increasing or decreasing the most rapidly. We identified the ages of the local minima and maxima within the lowess fit to the second derivative and used these ages to segment our record into discrete periods (Fig. S14). While ages roughly agree across homologs, we use the C_{31} ages to identify timeframes in the late Miocene as that chain length is the most abundant and represents a mixture between C_3 and C_4 sources.

S11: Establishing the Initial Age of C_4 Expansion

Polissar et al. (2019) found that C_4 ecosystems began to expand at 10 Ma at the earliest in NW Africa, and our results are largely consistent with this finding. They used the C_{35} chain length because this homologue is present in high abundances in C_4 grasses and they considered it a sensitive indicator for C_4 vegetation on the landscape. Those authors used z-scores calculated on each $\delta^{13}C$ C_{35} sample value compared to a middle and early Miocene reference range to make this determination. They selected the sample age at which the corresponding sample z-score rose above 2, indicating that this sample had a $\delta^{13}C$ value 2 standard deviations away from the reference range mean. This is an appropriate technique because subsequent samples had z-scores that continued to increase suggesting that this sample was the first member of the trend to more positive $\delta^{13}C$ values, and greater relative amounts of C_4 vegetation. Initial increases in C_4 vegetation would therefore first manifest in sedimentary *n*-alkane C_{35} $\delta^{13}C$ as observed in their (and our) record. We applied the same method outlined above but to all homologues (Fig. S16), using a reference range of 11.5 – 13 Ma. Results show a sustained increase above 2σ occurs first in C_{35} and C_{33} between 11 and 10 Ma, followed by C_{31}, C_{29} and C_{27} between 10 and 9 Ma. Therefore, we locate the beginning of the expansion of C_4 ecosystems to between 11 and 10 Ma, or ~ 10.4 Ma.

S12: Calculation and MDS Analysis of Late Miocene C_3 and C_4 Distributions

To understand changes in late Miocene – Pliocene C_3 and C_4 vegetation assemblages, we calculated the relative amounts of C_{27} – C_{33} contributing to the C_3 and C_4 component of our distribution record from 9.0 – 0 Ma. We first compiled all datasets for which multi-homolog *n*-alkane distributions and carbon isotope ratios were published for NW Africa from the Miocene to Recent (this study; O'Mara et al., 2024; Polissar et al., 2019). Other publications either focus on the $\delta^{13}C$ values of one chain length (usually C_{31}) or do not publish individual distributions and, hence, were not useful for our purposes. This criterion limited the chain lengths of interest to C_{33} through C_{27}, since O'Mara et al. (2024) did not publish C_{35} carbon isotope data. We selected a cut off age of 9.0 Ma for our analysis because that is the age after which all chain lengths show carbon isotope ratios that are reliably two standard deviations above the 11.5 – 13 Ma mean (see SI Appendix, section S11), and when C_4 vegetation is reliably affecting all four chain lengths of interest. We then constructed a two-endmember mixing model to calculate the fractional abundance of C_4 contributing to each chain length using sample *n*-alkane $\delta^{13}C$ values and estimates of mean C_3 and C_4 plant *n*-alkane carbon isotope ratios from a compilation of modern African and Indian plant waxes (Polissar et al., 2021). This calculation is shown in Eq. S3 where fC_4 is the fractional contribution of C_4, *n* stands for the homolog of interest and $\delta^{13}C_{(Cn-C4)}$ and $\delta^{13}C_{(Cn-C3)}$ are the modern C_4 and C_3 plant values for that homolog, respectively (taken from the compilation published in Polissar et al. (2021)) and $\delta^{13}C_{(Cn-meas.)}$ is the measured $\delta^{13}C$ value of that homolog in a sample:

(Eq. S3)

$$fC_{4(Cn)} = \frac{\delta^{13}C_{(Cn-meas.)} - \delta^{13}C_{(Cn-C3)}}{\delta^{13}C_{(Cn-C4)} - \delta^{13}C_{(Cn-C3)}}$$

Next, we multiplied the fC_4 value for each homologue by its relative C_{27} – C_{33} concentration (Eq. S4), where square brackets [ ] indicates relative concentrations. This allows us to estimate the relative concentration of that homolog which is due to C_4 plant contributions, and by difference the C_3 plant contributions (Eq. S5).

(Eq. S4)

$$[C_{n-C4}] = fC_{4(Cn)} * [C_n]$$

(Eq. S5)

$$[C_{n-C3}] = (1 - fC_{4(Cn)}) * [C_n]$$

We then normalized each C_4 and C_3 concentrations separately by summing across all homologues of interest in a sample and calculated the relative distribution of the C_3 and C_4 components (Eq. S6 and Eq. S7). This results in a separate, normalized, *n*-alkane distribution (C_{27} – C_{33}) for both the C_3 and C_4 components of each sample.

(Eq. S6)

$$[C_{n-C4norm}] = \frac{[C_{n-C4}]}{\sum_{n=27}^{33}[C_{n-C4}]}$$

(Eq. S7)

$$[C_{n-C3norm}] = \frac{[C_{n-C3}]}{\sum_{n=27}^{33}[C_{n-C3}]}$$

We used metric multi-dimensional scaling analysis (metric MDS) implemented in python with Scikit Learn's *sklearn.manifold.MDS* package to assess similarities and differences between the distributions of the C_3 and C_4 components as calculated in Eq. S6 and Eq. S7 (Pedregosa et al., 2011). MDS is a technique which attempts to find the pairwise distances between samples in a reduced dimensional space such that the stress function (a measure of the difference between the original distances between samples and their approximated distance in the reduce spaced) is minimized. Metric MDS is suitable for interval or ratio data (as ours is) because it takes the distance values between samples as meaningful (compared to non-metric MDS, which is applied to ordinal data). Further, metric MDS is useful for our purposes because it allows us to identify clusters of samples which are dissimilar from each other (our goal), while also allowing for the expression of gradation in this difference, unlike other clustering techniques (such as hierarchical clustering). We further used *sklearn.manifold.MDS* to reduce our four-dimensional datasets (one each for the C_3 and C_4 component) into two dimensions using the Euclidean distance with a stress tolerance of $1x10^{-6}$ (the default value). This analysis results in the clustering of data into two major groups in both the C_3 and C_4 component corresponding roughly to sample age (Fig. S17A and B). Distributions show a strong tendency to separate out into (1) a dominantly late Miocene cluster and (2) a dominantly Pleistocene cluster, with the Pliocene samples clustering either with the Miocene samples as in the C_3 component or with the Pleistocene samples as in the C_4 component (Fig. S17A and B). It is unlikely this analysis is simply identifying the different datasets (our new data; O'Mara et al., 2024; Polissar et al., 2019) as the Polissar et al. (2019) dataset, which is the only to span the late Miocene and Plio-Pleistocene, splits between the two clusters. We take this analysis as evidence that the C_3 and C_4 components of the *n*-alkane pool contributing to the sedimentary archive changed between the late Miocene and Pleistocene, suggesting that the plants on the landscape during the different time periods did as well. We repeated this analysis using homologs C_{29}, C_{31}, and C_{33}, (Fig. S17C and D) and results replicate this main finding.

S13: Compilation and Analysis of Tropical and Subtropical C_4 Expansions

To evaluate the degree to which C_4 vegetation expansion in the subtropics and tropics was globally coeval on the million-year timescale, we compared estimations of C_4 vegetation development for fifteen regions from the literature (Andrae et al., 2018; Dupont et al., 2013; Feakins et al., 2013; Feakins et al., 2020; Fox and Koch, 2004; Hoetzel et al., 2013; Huang et al., 2007; Latorre et al., 1997; Passey et al., 2009; Polissar et al., 2021; Uno et al., 2016; Zhou et al., 2017). For this analysis, we were primarily interested in identifying and comparing the timing of greatest increase in $\delta^{13}C$ in each record, which we refer to as the timing of "C_4 expansion", as this marks the age interval when C_4 vegetation spread the fastest and were likely the most favored. We also undertook estimating the (often earlier) timing of initial increase in C_4 vegetation for each region, even if that increase was minor. This is a more difficult age to estimate, as it is heavily influenced by the resolution of the record and the type of data that are available. We refer to this age as the "inception age" of C_4 ecosystems for the region.

To make this compilation, we focused on studies that used the carbon isotope ratios of plant waxes recovered from marine sediments because (1) this proxy integrates a regional signal and (2) is a direct reflection of past vegetation, making it useful for reconstructing broad trends in C_4 dynamics on the landscape. If no such study existed for a region (the case for 5 localities), we then relied on records of soil carbonate $\delta^{13}C$ instead. We avoided studies of tooth enamel $\delta^{13}C$

for estimates of C_4 expansion because herbivores can be selective in their diets and their dietary consumption habits may not directly reflect the balance of C_3 vs C_4 vegetation on the landscape (Uno et al., 2011). In most cases, we relied on the original authors' interpretations for estimations of the timing of C_4 expansion, with several exceptions. For the Chinese Loess Plateau (Passey et al., 2009), Nepal (Polissar et al., 2021), Arabian Peninsula / Himalayan foreland (Huang et al., 2007), east Africa (Uno et al., 2016), South America (Latorre et al., 1997) and western South Africa (Dupont et al., 2013), we estimated the beginning and ending ages of C_4 expansion from the authors' published figures and data. We identified the interval of most rapid increase in $\delta^{13}C$ of each plant-wax or soil carbonate record and used the oldest age of that interval as the beginning age of the C_4 expansion and the youngest age as the ending age of the C_4 expansion. We used the age estimates detailed in Polissar et al. (2019) for the inception age of C_4 vegetation for each region, excepting the Indian subcontinent (the Indus Fan and Bengal Fan), the Arabian Peninsula/ Himalayan foreland, and NW Africa. In those cases, we used the original authors' estimates for initial C_4 ecosystem inception. This varied approach was taken because both studies that investigate the Indian subcontinent (Feakins et al., 2020; Polissar et al., 2021) were published after the analysis of Polissar et al. (2019), the Arabian Peninsula/Himalayan foreland dataset was not included in that study, and we made minor updates to the inception age of C_4 ecosystems in NW Africa in this study (see SI Appendix, section S11). We note that for both the east African and northeast African regions, we used the inception age of Polissar et al. (2019) as the beginning age for C_4 expansion (9.647 and 9.649 Ma, respectively), as these authors' reanalysis of inception ages occurs somewhat later than the original authors' estimates of C_4 expansion (10 and 11 Ma, respectively).

These approximations are suitable for our purposes because we are interested in the successive C_4 expansion over millions of years, and thus precision on the order of 10^5 years is not necessary for these comparisons. However, if a region had less than three data points per million years during the time when $\delta^{13}C$ was increasing the most rapidly, which was the case for Australia (Andrae et al., 2018), Nepal (Polissar et al., 2021), northeast Africa (Feakins et al., 2013), east Africa (Uno et al., 2016), and the North American Great Plains (Fox and Koch, 2004), we plotted it in grey with white hatches on Figure 4. This was done to indicate those regions for which estimating the timing of greatest increase in C_4 was difficult due to the data resolution or coverage. We used the latitude and longitude of the IODP core site, or main study site if such existed, to assign a latitude and longitude value to each region. If none was given, we estimated latitude and longitude from the maps provided in each publication, matching study sites to coordinates using Google Earth. These latitude values were then used to plot the data in Figure 4 from north to south.

All statistical analyses were implemented in python, version 3.11.13.

Supporting Figures

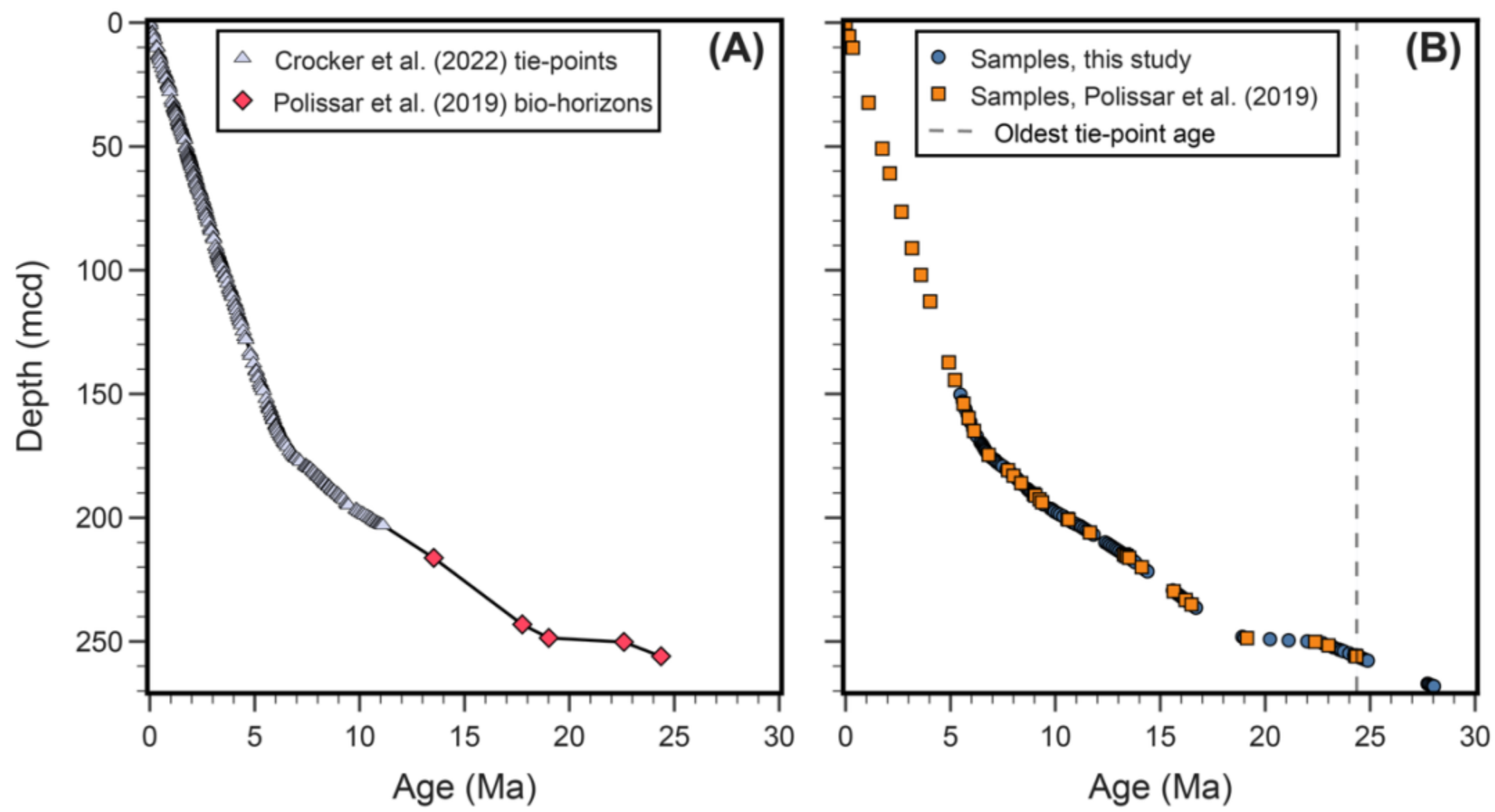

Fig. S1. Updated age model tie-points (A) and sample ages (B) for ODP 659, modified from Polissar et al. (2019) and Crocker et al. (2022). In (A), light grey triangles are tie-points generated by tuning XRF geochemical measurements to summer insolation (Crocker et al., 2022) and red diamonds are nannofossil biohorizons (Polissar et al., 2019). In (B), blue circles are new sample ages calculated by linear interpolation between tie-points after sample depths have been placed on an updated meter composite depth scale (mcd). Orange squares are the reanalyzed ages of samples from Polissar et al. (2019). The grey dashed line is the age of the last tie-point used for linear interpolation shown in (A), and all samples to the right of the line have extrapolated ages.

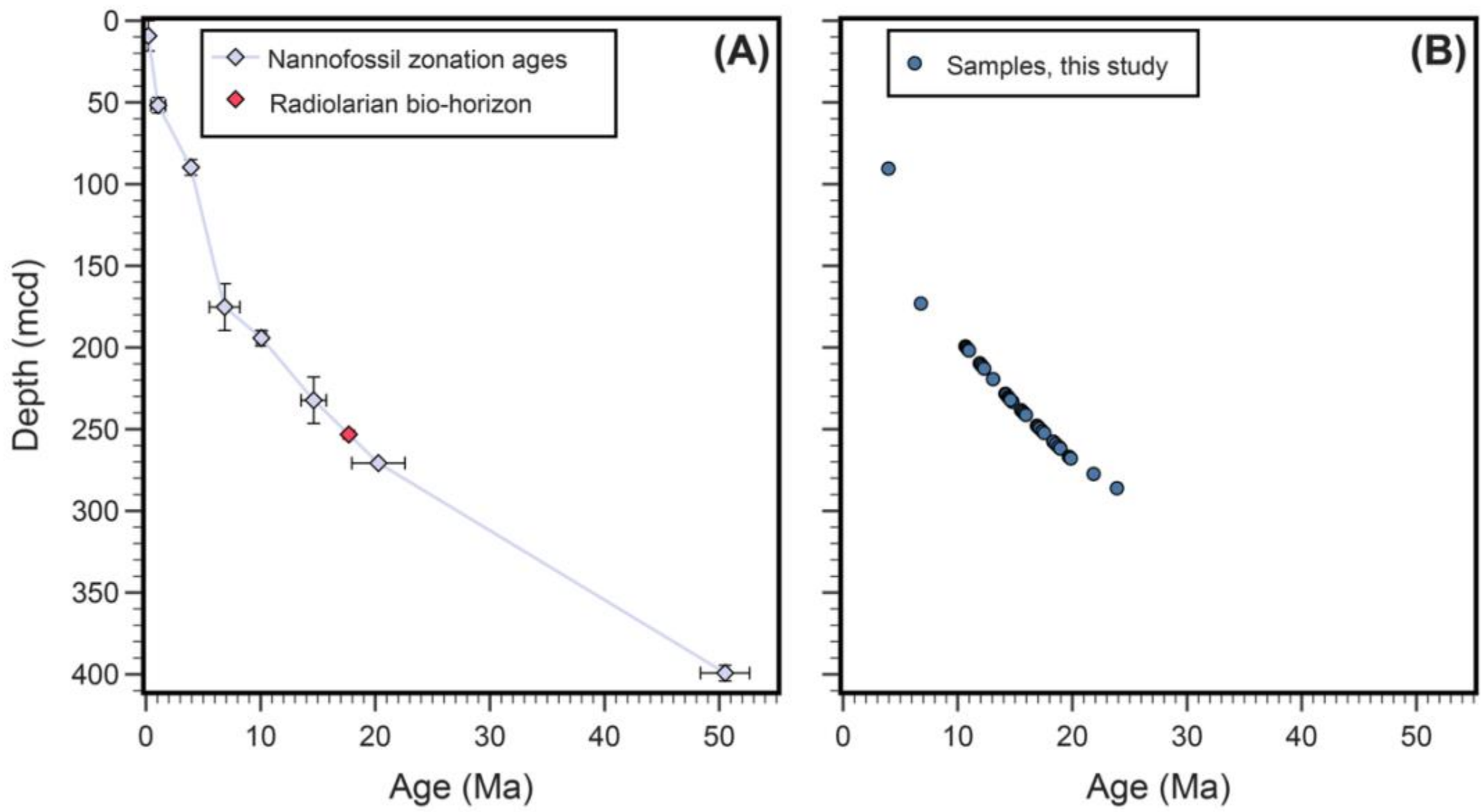

Fig. S2. Age model for DSDP 368 showing age-depth tie-points (A) and inferred sample ages (B) calculated by linear interpolation between tie-points. In (A), the light grey diamonds are averaged nannofossil zonation ages, and the red diamond is the single, well-constrained radiolarian biohorizon. The horizontal error bars represent the entire age range of the identified nannofossil zonation, while the vertical error bars represent the depth range in which that nannofossil zonation was identified.

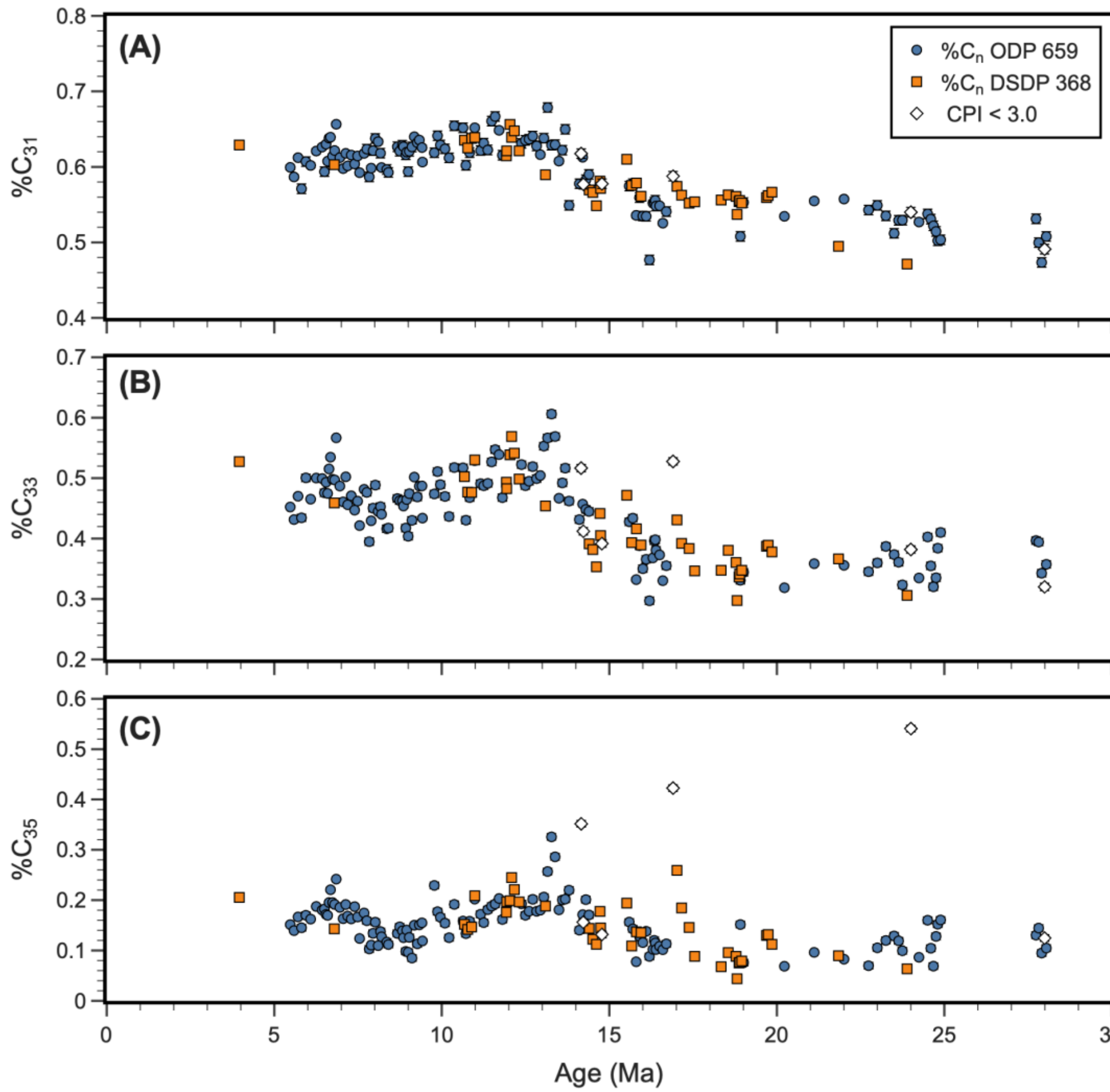

Fig. S3. $\%C_{31}$, $\%C_{33}$ and $\%C_{35}$ through time (plotted in A, B, and C, respectively), separated by core site. Samples from ODP 659 are blue circles, samples from DSDP 368 are orange squares, and samples with CPI < 3.0 are displayed with open symbols in all three plots. Samples from both core sites largely agree on timing of transition from lower values pre 16 Ma to higher values post 14 Ma. Note the different y-axis scales in all three plots.

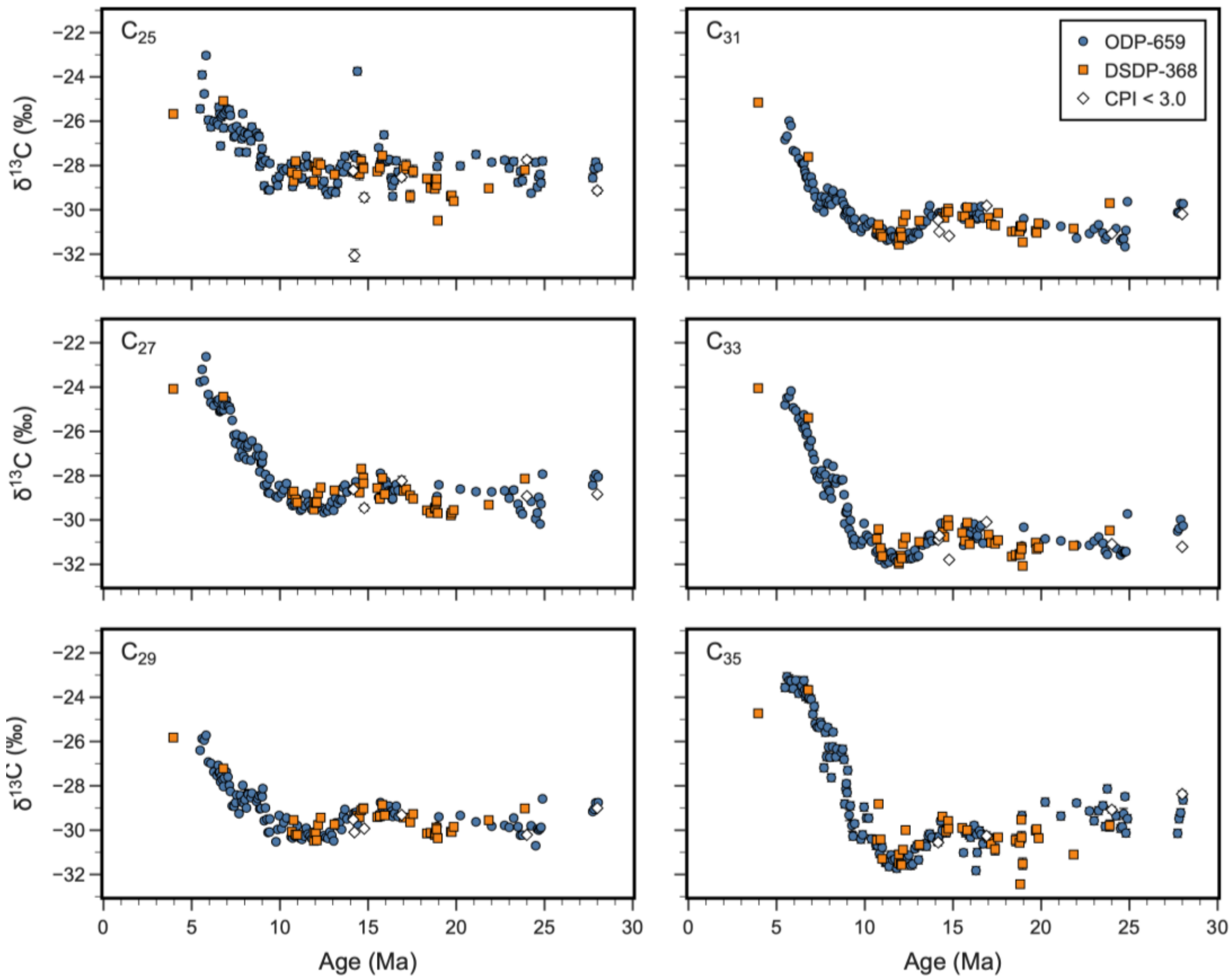

Fig. S4. Carbon isotope ratios of odd *n*-alkanes, C_{25} – C_{35} (uncorrected for changes in atmospheric $\delta^{13}C$). Samples from ODP 659 are blue circles, samples from DSDP 368 are orange squares, and samples with CPI < 3.0 are displayed with open symbols in all three plots. Sample $\delta^{13}C$ values show good agreement between both core sites across all homologs.

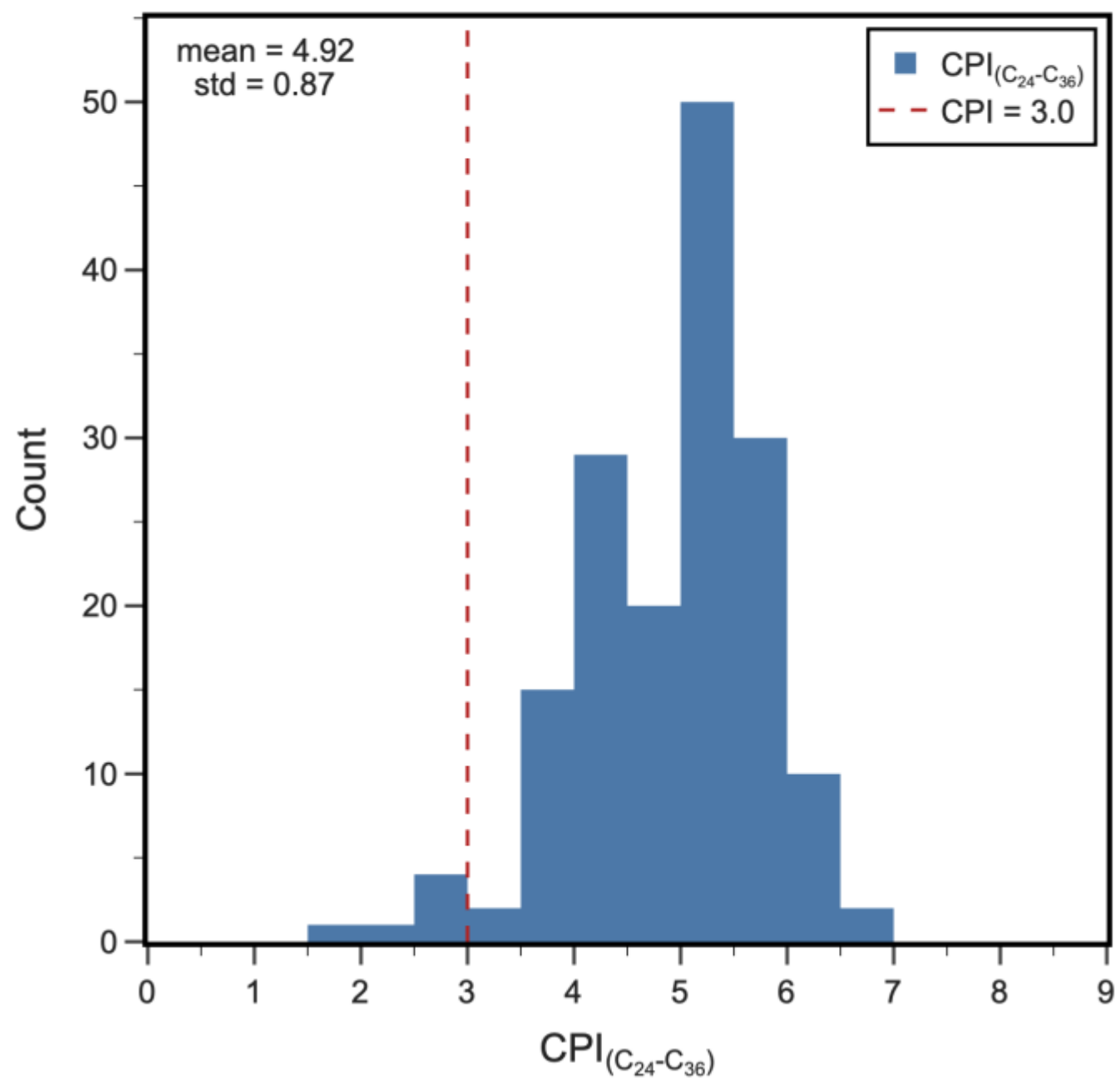

Fig. S5. Histogram of all Carbon Preference Index values ($CPI_{(24\text{-}36)}$), calculated from abundances of *n*-alkanes C_{24} – C_{36}. Red dotted line indicates the value below which samples were treated as potentially deviating from a primary plant wax signal (3.0). The mean and one sigma standard deviation (std) of all $CPI_{(24\text{-}36)}$ values are displayed at the top left.

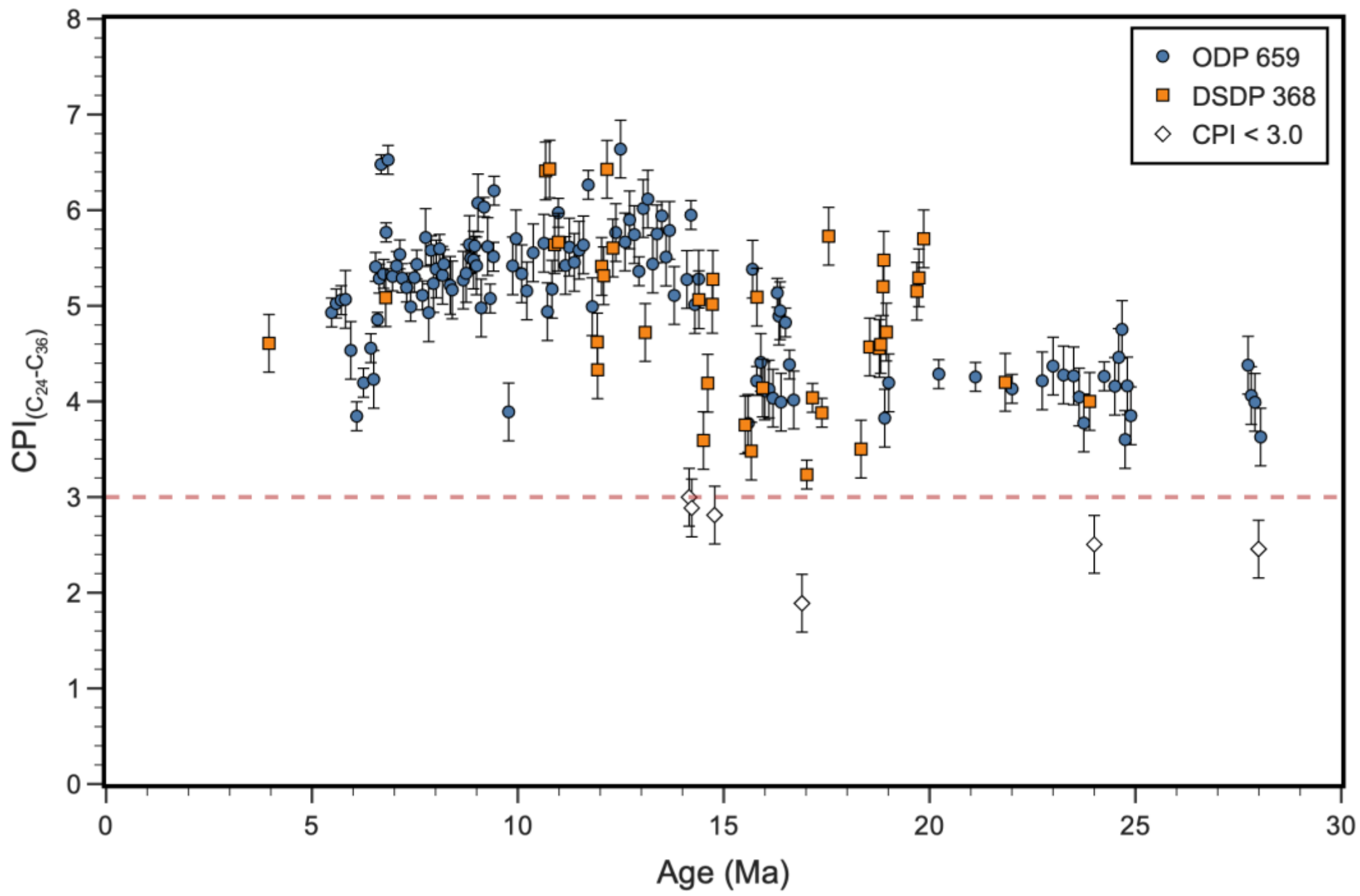

Fig. S6. $CPI_{(24-36)}$ values for all samples through time separated by core site (ODP 659 = blue circles; DSDP 368 = orange squares). The uncertainty is one standard error of the mean across all pooled replicates. Samples with $CPI_{(24-36)} < 3.0$ are indicated with open symbols below the dashed line.

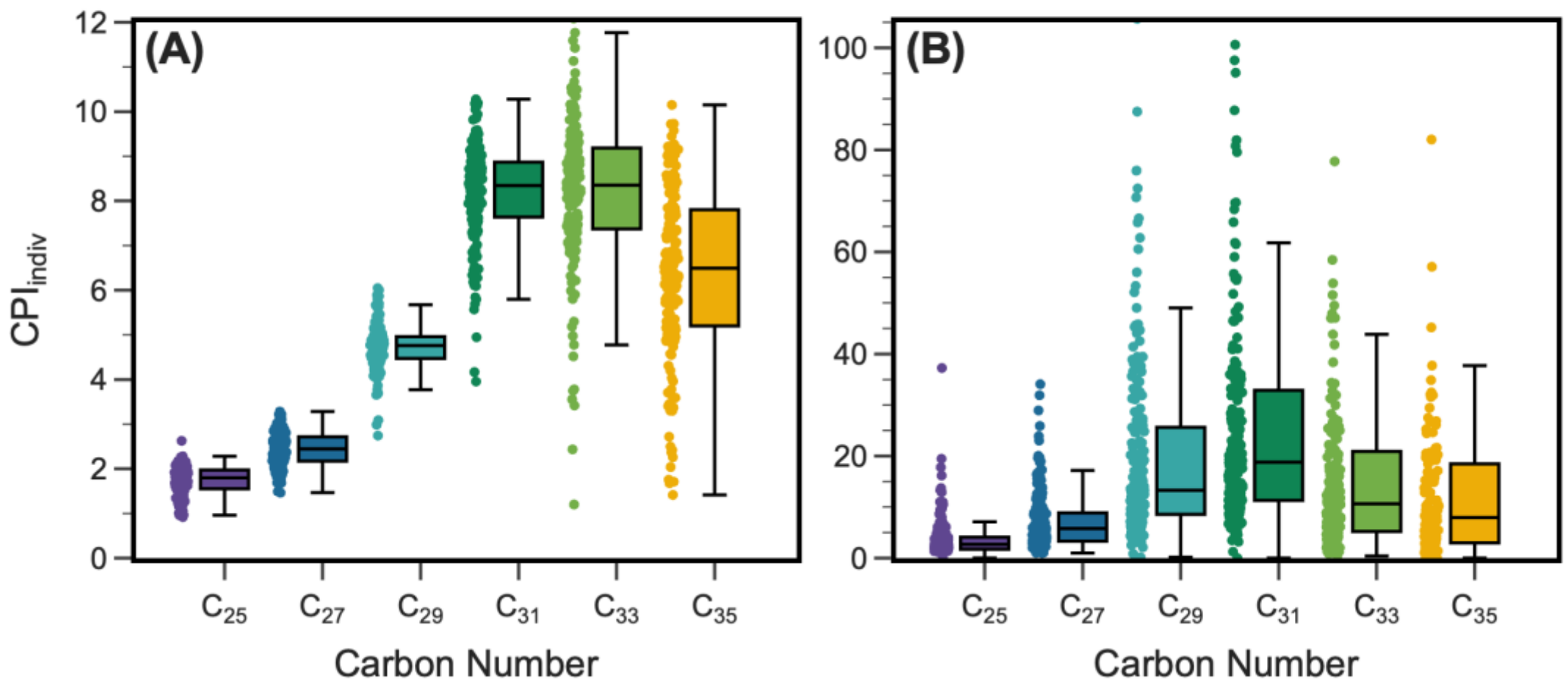

Fig. S7. Boxplots of sample CPI individual (CPI_{indiv}) values from ODP 659 and DSDP 368 in figure A, and modern African plants in figure B (Tweedy et al., 2025) (note the different y-axis scales). Longer chain lengths (C_{29}, C_{31}, C_{33}, and C_{35}) have higher measures of center and greater variability than lower chain lengths (C_{25}, C_{27}) in both plots. This suggests that sedimentary C_{25} and C_{27} may exhibit lower CPI individual values in part because of the nature of plant *n*-alkane production, and does not only represent thermal maturation, diagenesis or contamination from other sources.

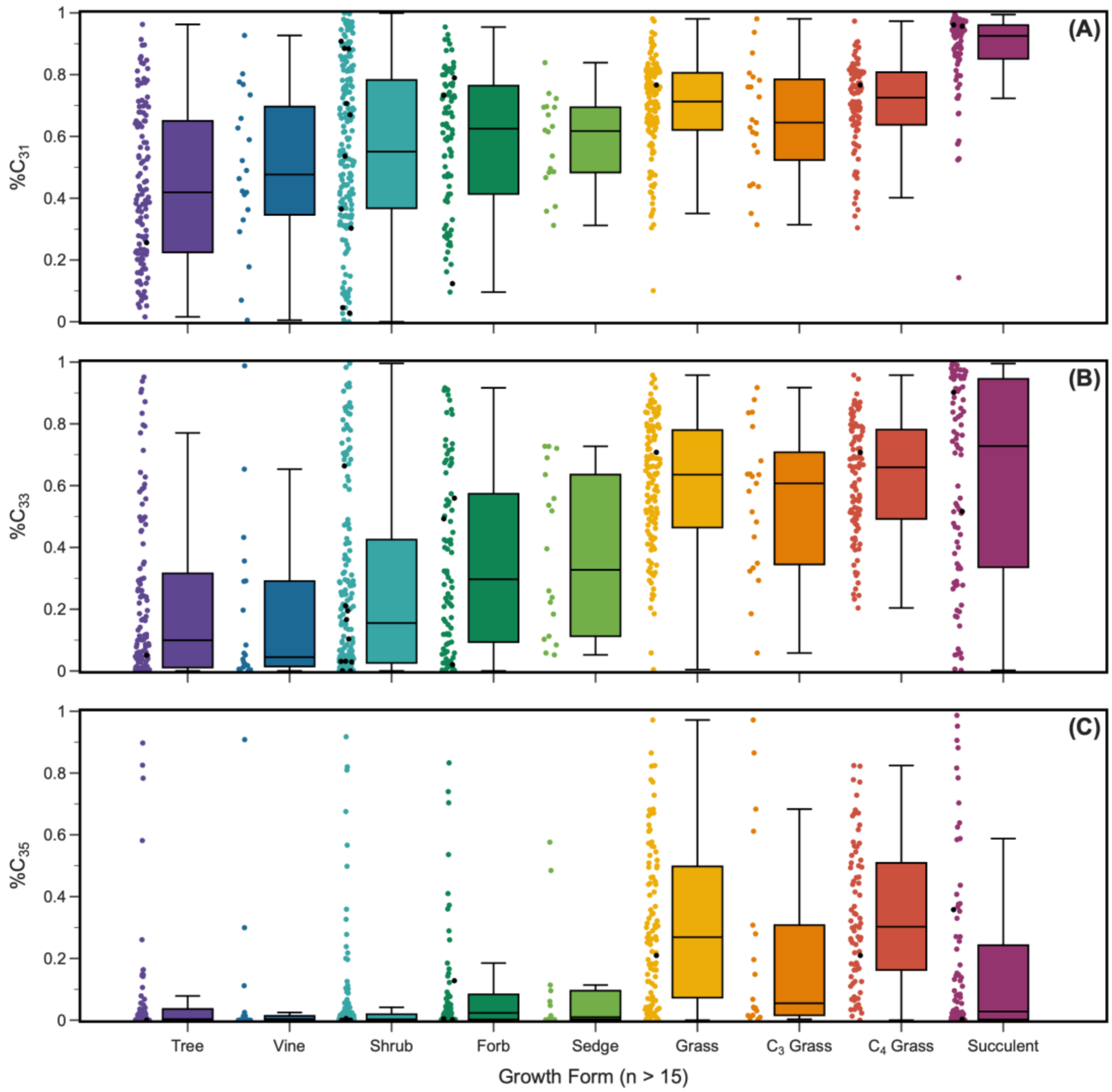

Fig. S8. Boxplots of modern African plant *n*-alkane %C_{31}, %C_{33}, and %C_{35} compiled from the literature and shown in A, B and C, respectively (Ali et al., 2005; Badewien et al., 2015; Bezabih et al., 2011; Dodd et al., 1995; Garcin et al., 2014; Gensel et al., 2022; Griepentrog et al., 2019; Kristen et al., 2010; Magill et al., 2019; Rommerskirchen et al., 2006; Sonibare et al., 2010; Tweedy et al., 2025; Vogts et al., 2009). Woody plants, such as trees and shrubs, have distributions that are skewed lower than those of grasses for all three metrics, particularly %C_{33} and %C_{35}. Taxa that could not be identified and were excluded from the analysis are shown with black dots.

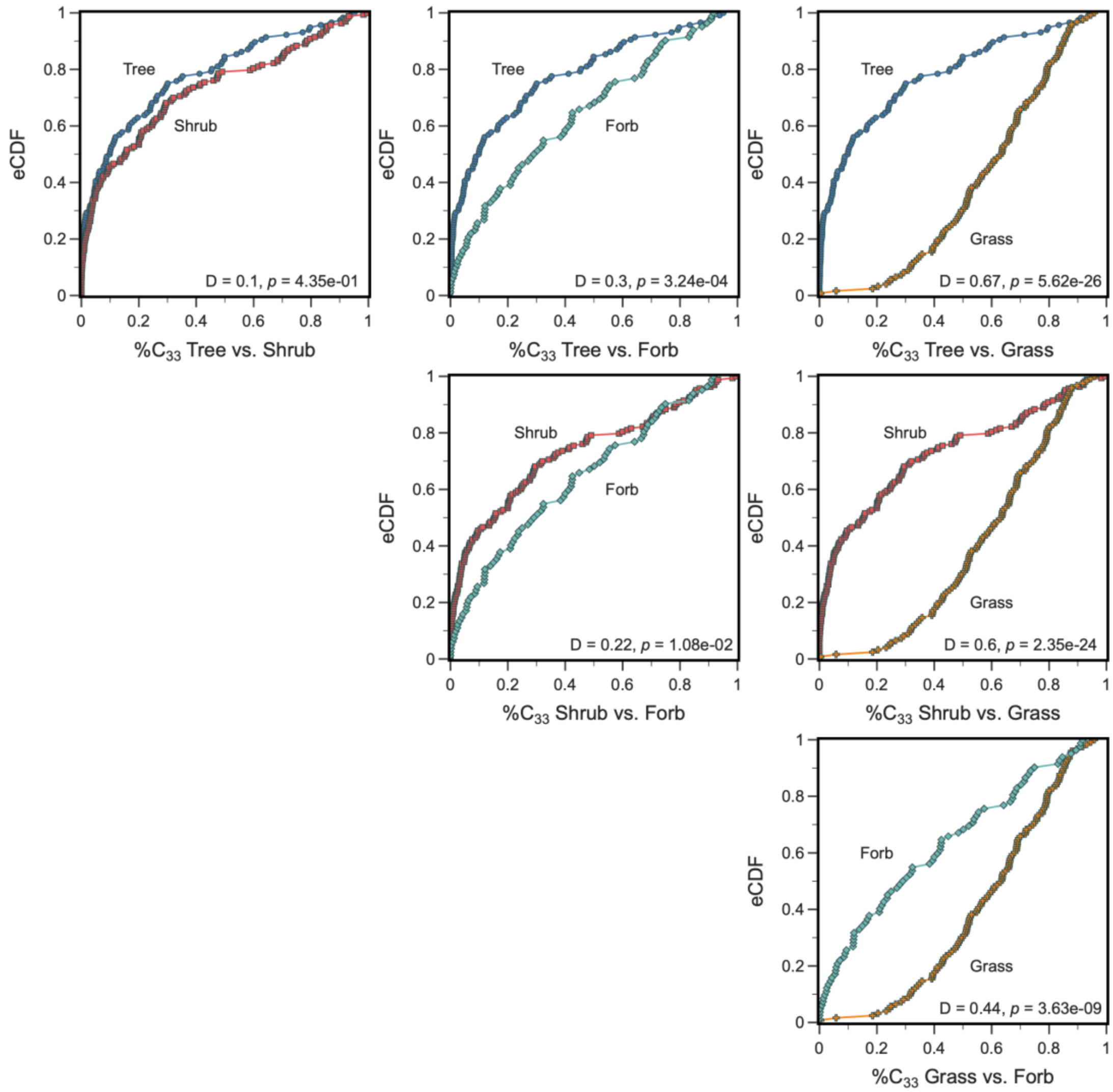

Fig. S9. Results of two-tailed Kolmogorov-Smirnov tests comparing distributions of %C_{33} by growth form. In all plots, the eCDF (empirical cumulative distribution function) is labeled with its corresponding growth form. The test statistic (D) and *p*-value of each Kolmogorov-Smirnov test is shown in the lower right. Grasses have very different distributions than trees, shrubs or forbs.

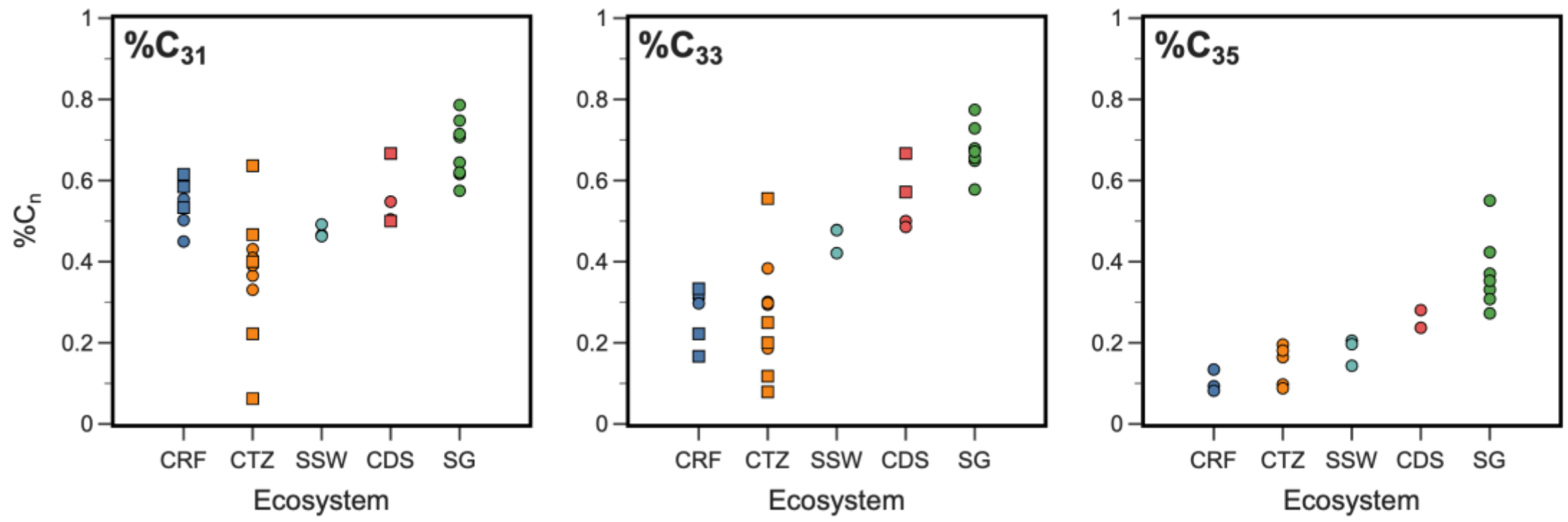

Fig. S10. Modern soil and sediment %C_n values from an ecosystem transect in Cameroon (Garcin et al., 2014; Garcin et al., 2012; Schwab et al., 2015) and grasslands and savannas in the Serengeti (Zhang et al., 2021). Those samples sourced from ecosystems that contain more woody plant material (left side each plot: forests, transitional ecosystems, woodlands, or shrublands) generally have lower metric values than those ecosystems where grasses are a larger component of the plant community (right side of each plot). CRF = Cameroon rain forest; CTZ = Cameroon transition zone; SSW = Serengeti shrublands and woodlands; CDS = Cameroon dry savanna; SG = Serengeti grassland. Cameroon data exclude all values for the "Bale" site as Garcin et al. (2014) report that this site was within a planted eucalyptus forest and Schwab et al. (2015) excluded it from analysis. Serengeti data are from the topmost soil depths reported at each site. Circles represent data from soils and squares represent data from lake sediments.

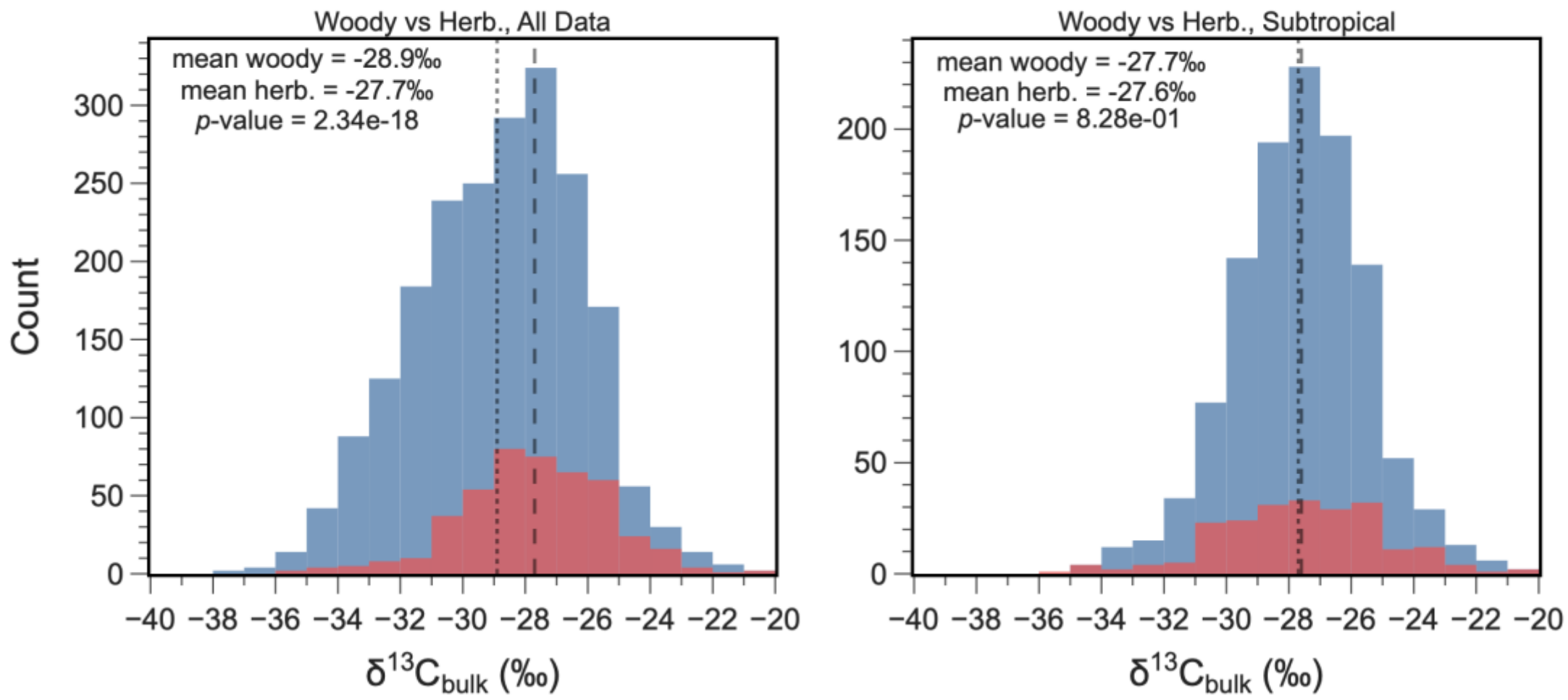

Fig. S11. Histograms of leaf $\delta^{13}C$ from a global compilation (Cornwell et al., 2018) for woody (tree, shrub, shrub/tree) growth forms in blue and herbaceous growth forms (forbs, graminoid, grass) in red. The left panel includes all data, while the right panel includes just those samples that occurred between 10 – 40°N or S of the equator. The dotted line is the mean of the woody growth form distribution while the dashed line is the mean of the herbaceous growth form distribution. The mean $\delta^{13}C$ value of woody and herbaceous subtropical plants are not significantly different at the 5% confidence level (two-tailed t-test, $p = 0.83$). Note the different y-axis scales.

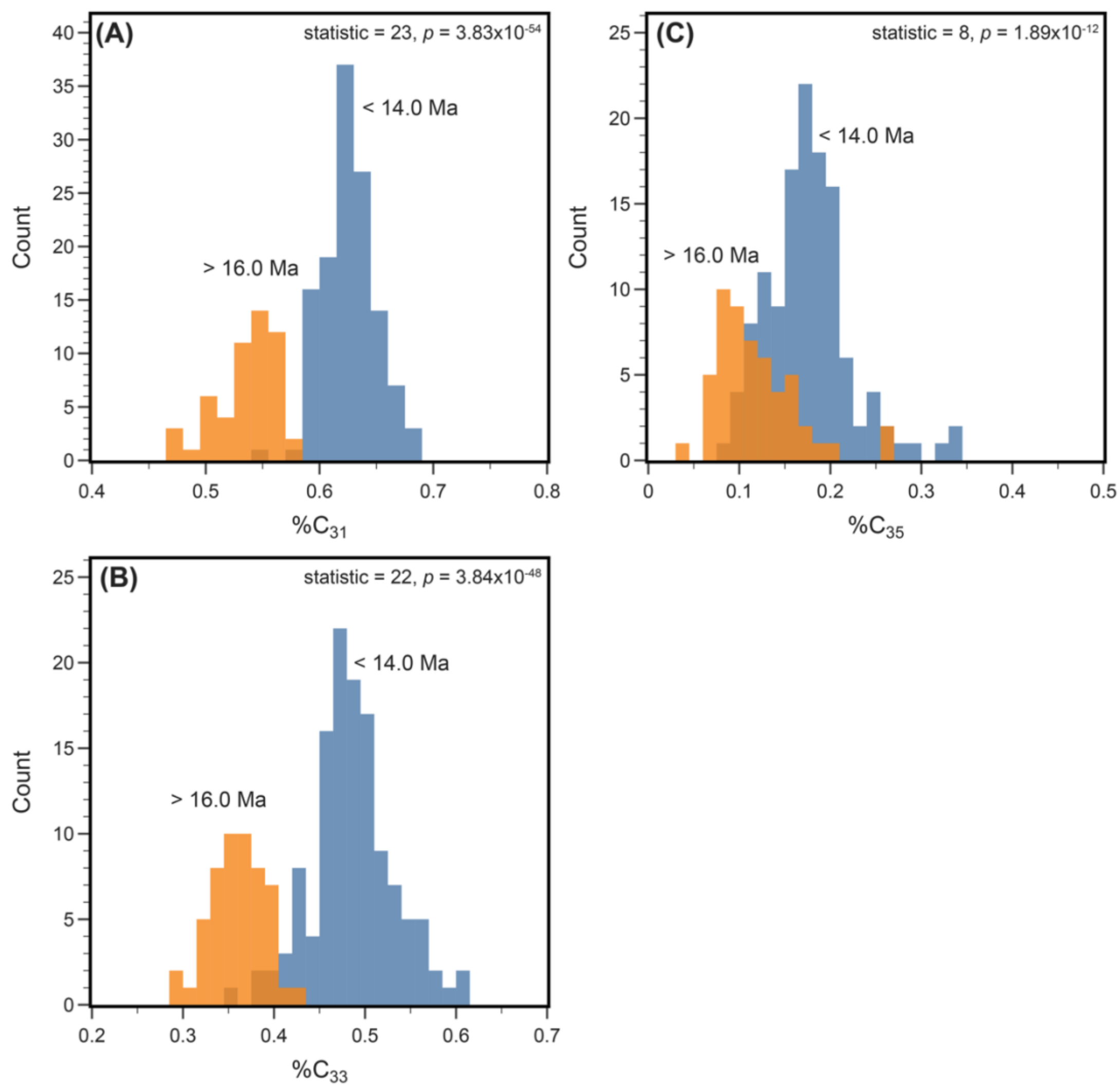

Fig. S12. Histograms of $\%C_{31}$, $\%C_{33}$, and $\%C_{35}$ separated by age of sample in A, B and C respectively. Blue histograms represent samples that are less than 14 Ma, and orange histograms samples that are greater than 16 Ma. Data includes new measurements integrated with results published in Polissar et al. (2019). Results (test statistic and *p*-value) of two tailed t-tests comparing distributions are shown at the top right and indicate statistically significant separation between sample distributions of $\%C_n$ based on age.

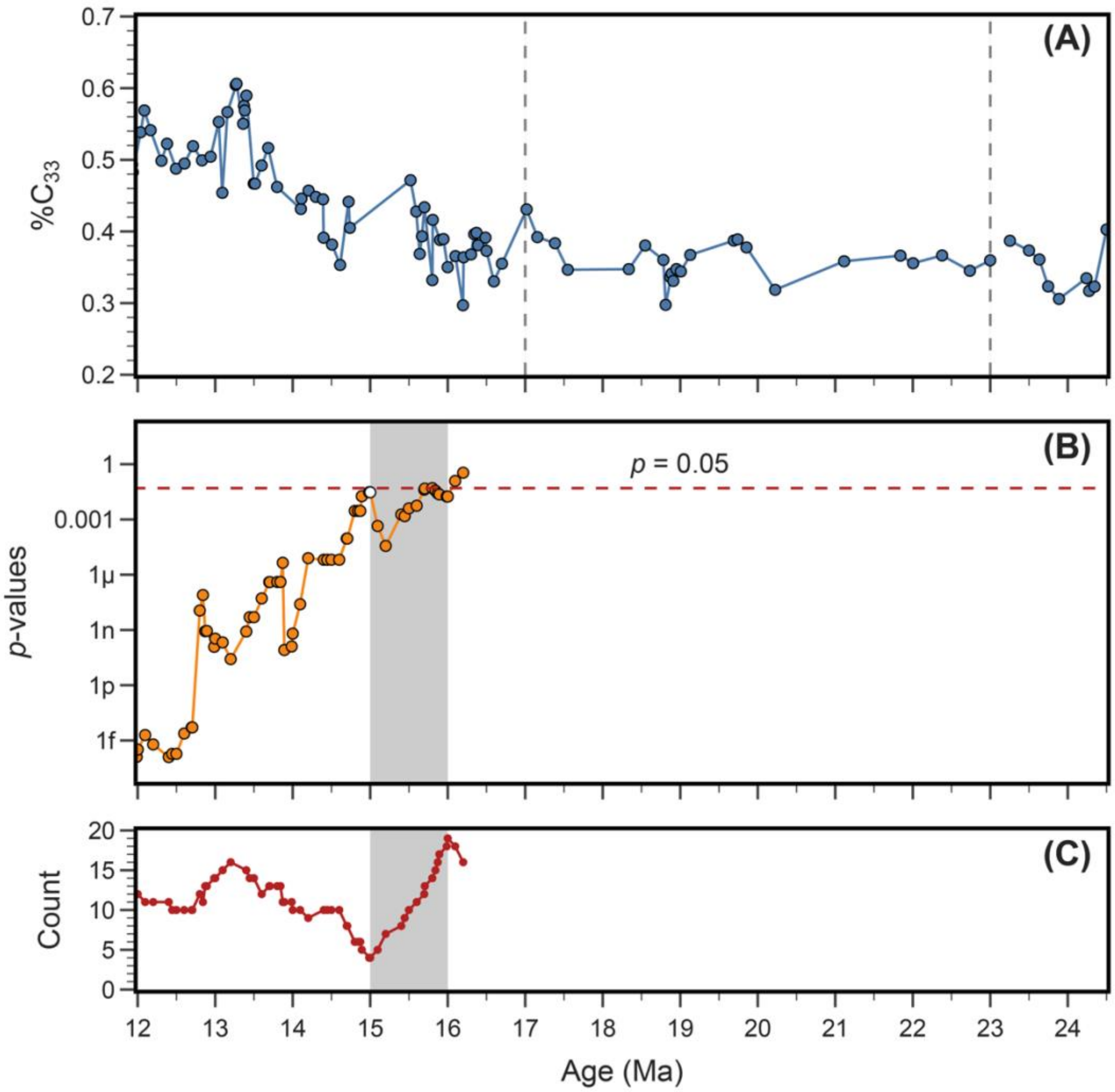

Fig. S13 A. Results of running student t-tests comparing sample %C_{33} values in 1-myr bins against a reference range of 17 – 23 Ma. %C_{33} values are shown in panel A and are comprised of new data and those published in Polissar et al. (2019).The dotted lines enclose the samples that were used to calculate the reference range. Plot B shows the *p*-values of the running t-tests with each point plotted in the middle of the sample window age range. A *p*-value of 0.05 is indicated with the dashed red line. Open circles are those *p*-values that were calculated on a sample window of $n < 5$ points. Plot C shows the count of each sample window. In plots B and C, the grey box indicates the sample windows that fall between 15 and 16 Ma, and that are not always significantly different from the reference range.

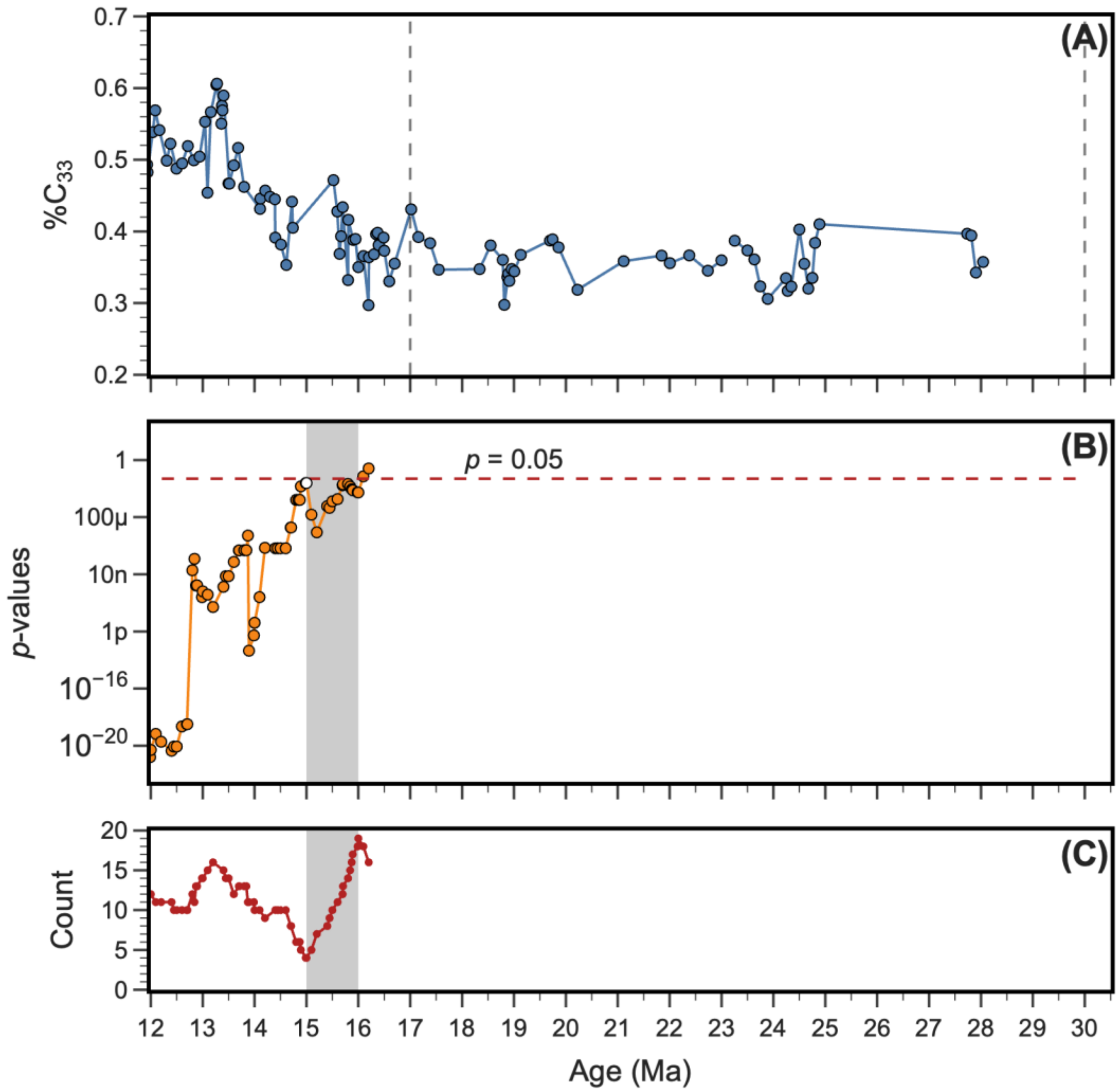

Fig. S13 B. Same as in Fig. S13A except reference range includes all samples between 17 Ma and the end of the record. Results of the running t-tests are invariant to the inclusion or exclusion of Oligocene aged samples.

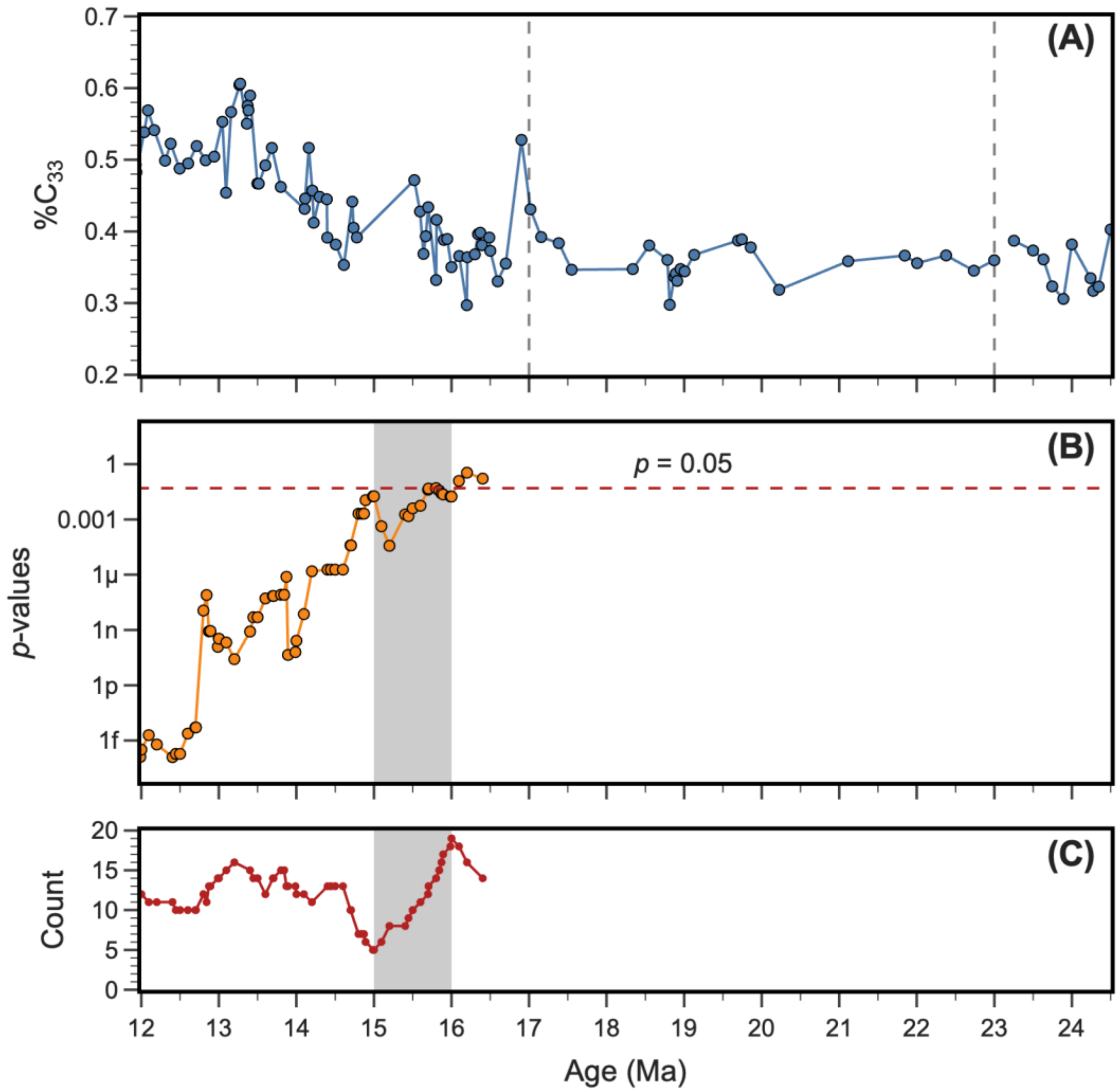

Fig. S13 C. Same as in Fig. S13A except t-tests now include the six samples that were originally screened out because they had a CPI < 3.0. Including these six samples does not meaningfully affect the results of the t-tests or the interpretations.

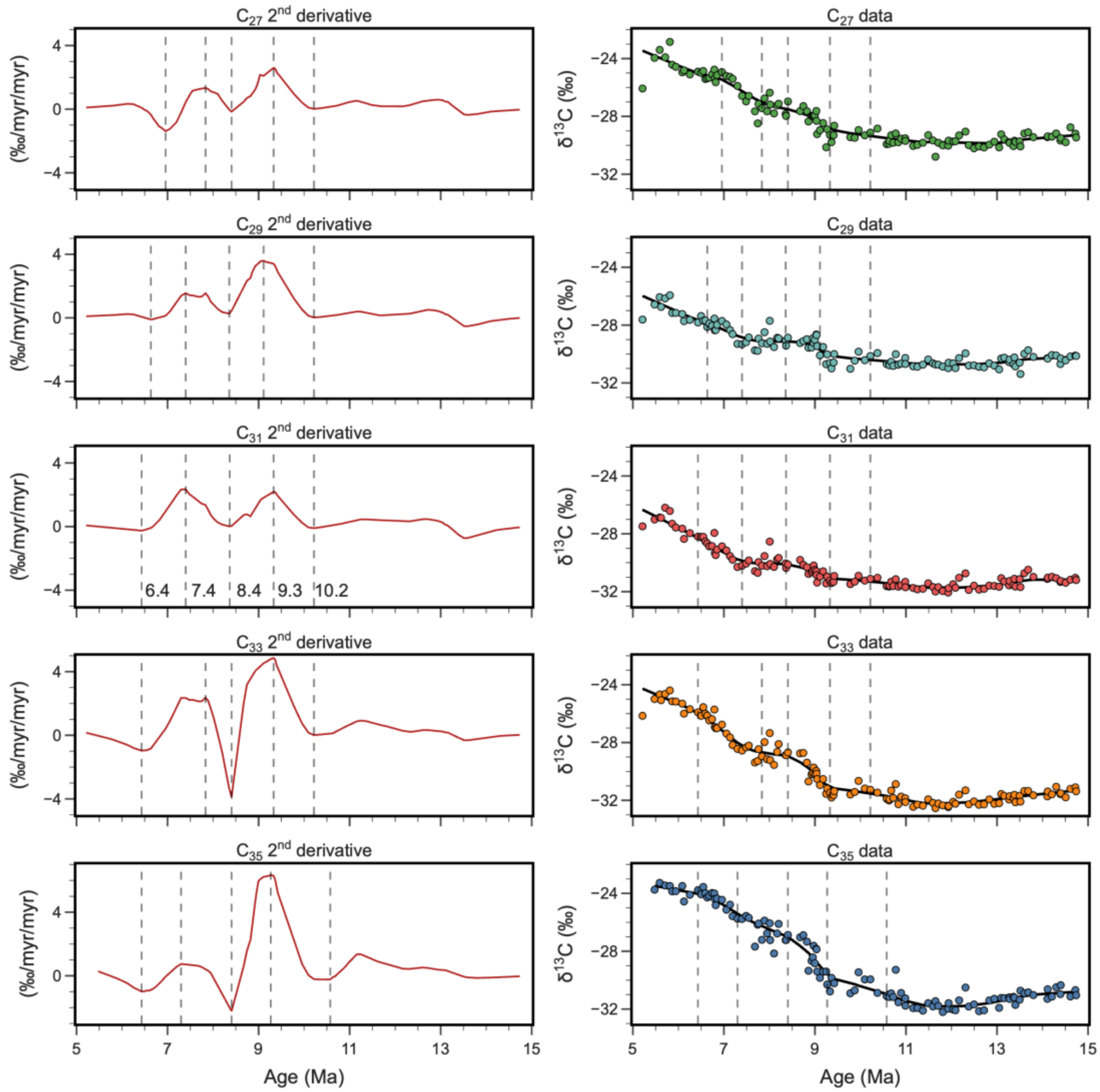

Fig. S14. Carbon isotope ratios of C_{27} – C_{35}, odd (right-hand column) and smoothed second derivative of a locally weighted scatterplot smoothing (lowess) curve fit to the data (left-hand column). In the right-hand column, data points are represented by colored circles, and the black lines are the lowess fits. In the left-hand column, the red line represents the smoothed second derivative. Dashed vertical lines are the local minimum and maximum points of the second derivative. Ages of the local minimum and maximum points are labeled to the right of the dashed lines in the left-hand C_{31} plot, and represent the approximate ages for C_4 expansions (9.3 – 8.4 Ma and 7.4 – 6.4 Ma) and pause (8.4 – 7.4 Ma) used in this study.

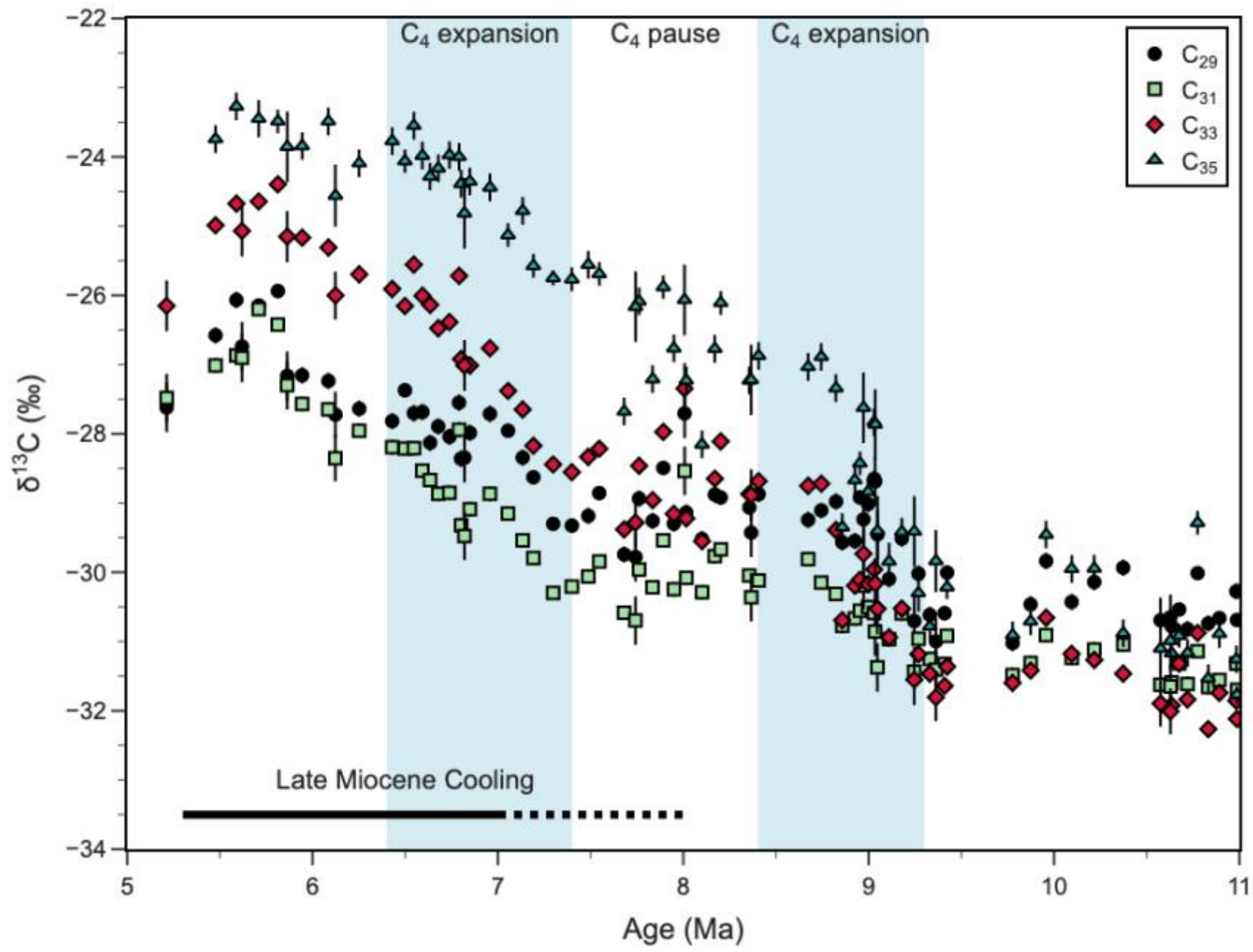

Fig. S15. The carbon isotope record of the late Miocene showing the pulsed increase in $\delta^{13}C$ between 9.3 – 8.4 Ma and 7.4 – 6.4 Ma. The pattern is apparent in all chain lengths but most well-developed in C_{35} and C_{33}, which are biased towards herbaceous vegetation and grasses.

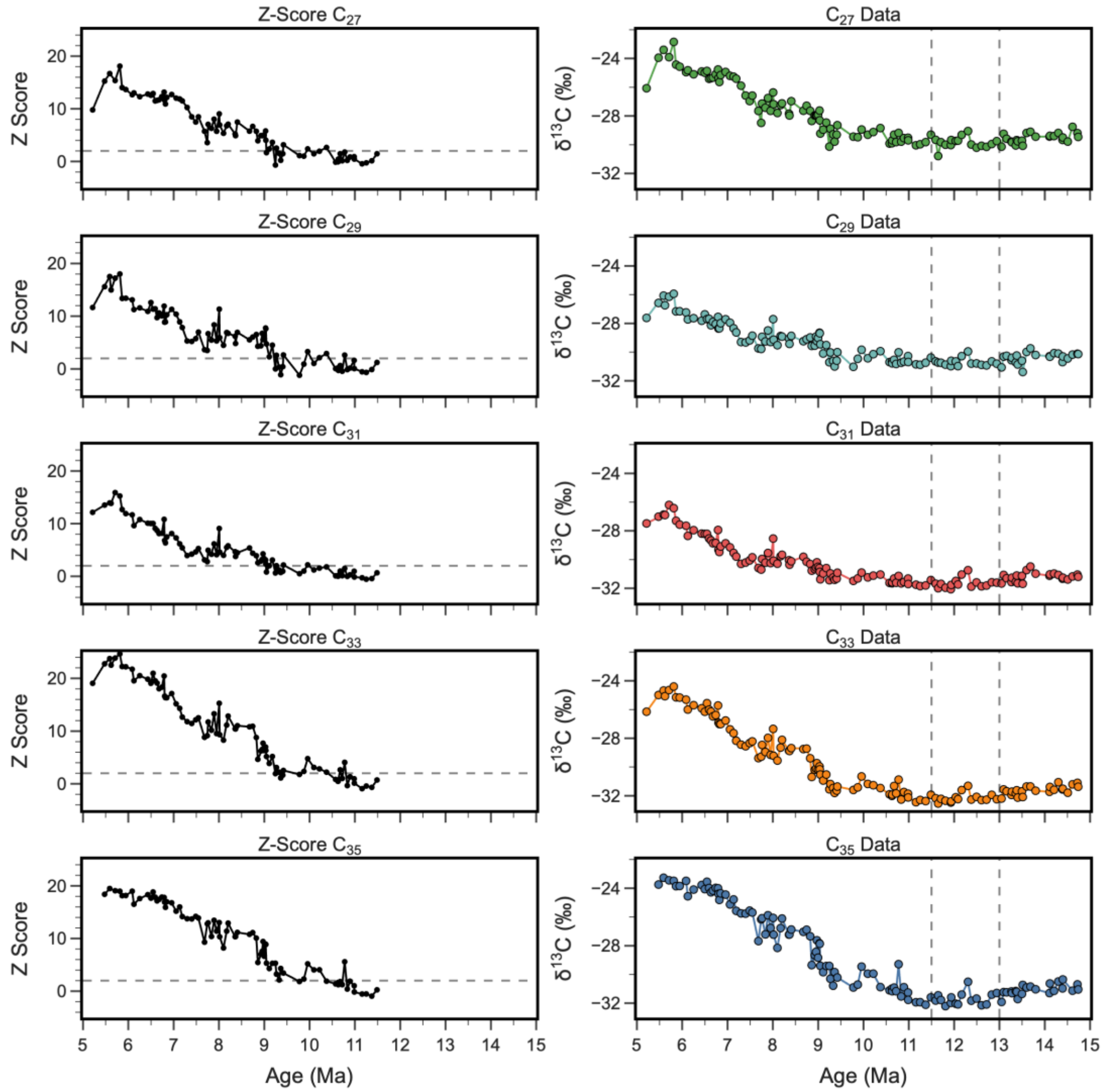

Fig. S16. Carbon isotope ratios for homologs C_{27} – C_{35}, odd (right-hand column) and Z scores (left-hand column) calculated for each $\delta^{13}C$ value from the 11.5 – 5.0 Ma interval compared to a reference distribution from the 13.0 – 11.5 Ma interval. In the right-hand column, dashed grey lines indicate the samples used to calculate the reference range mean and standard deviation. In the left-hand column, the dashed horizontal line is drawn at 2. Points above this line have values that are two standard deviations more positive than the reference distribution.

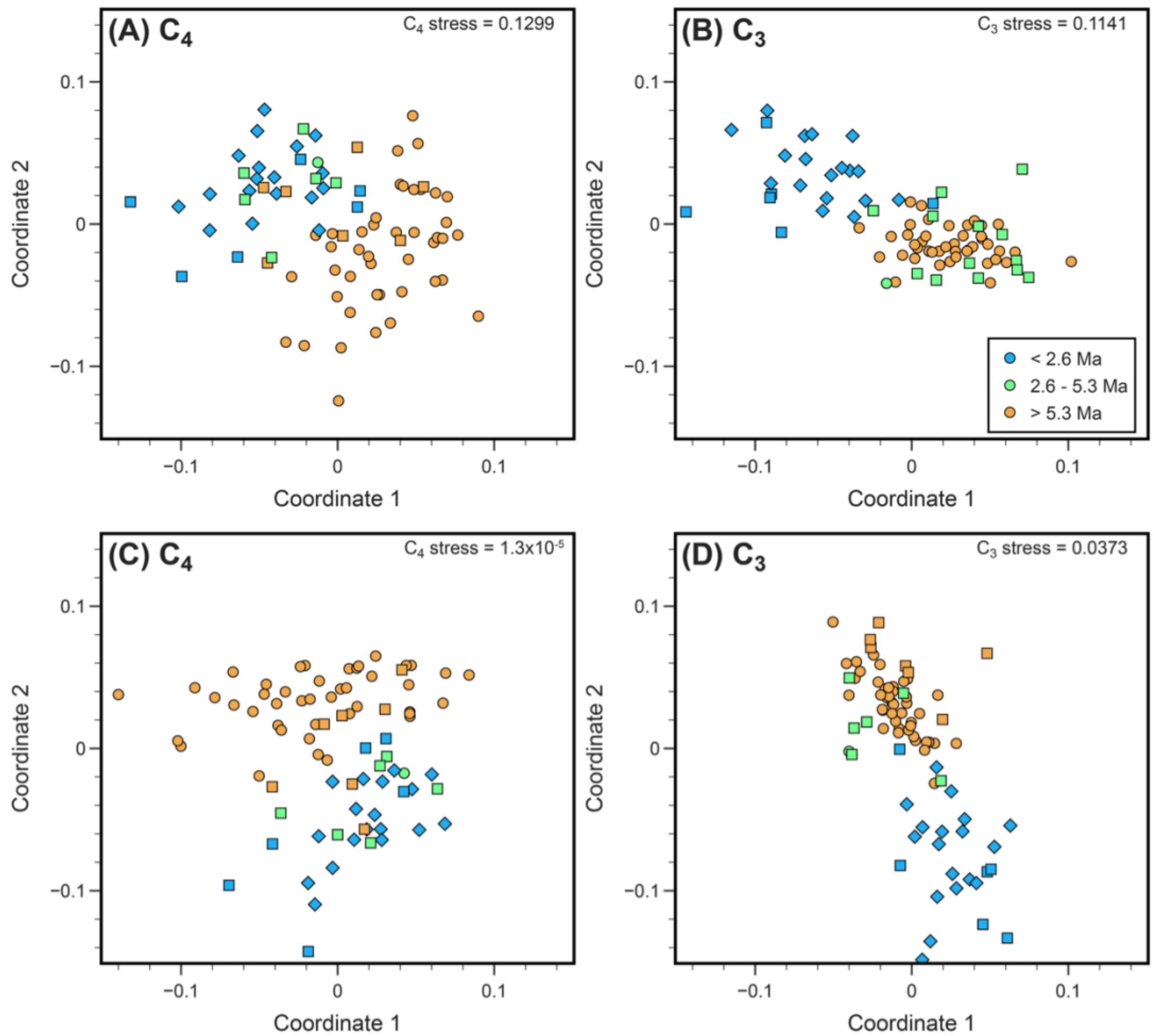

Fig. S17. Multidimensional scaling analysis (MDS) of the inferred distributions of the C_3 and C_4 plant components (right and left plots, respectively) from 9.0 Ma to Recent, including data from this publication, Polissar et al. (2019), and O'Mara et al. (2024) shown with circles, squares, and diamonds, respectively. Plot A and B show results from the analysis using the C_{27} – C_{33} homologs, while plot C and D shows results of the analysis using C_{29} – C_{33} homologs only. Samples are colored by age: Miocene samples are orange, Pleistocene samples are blue, and Pliocene samples are light green. The value of the minimized stress function, the residual sum of squares, for each MDS analysis is shown at the top right.

Supporting Tables

Table S1. P-values produced from the series of two-sample Kolmogorov-Smirnov tests evaluating growth form %C_n distributions against each other. Bolded values are those that fall below the 1% confidence level.

	%C_{31}			%C_{33}			%C_{35}		
Growth Form	Shrub	Forb	Grass	Shrub	Forb	Grass	Shrub	Forb	Grass
Tree	**0.00528**	**6.49×10^{-4}**	**9.58×10^{-13}**	0.435	**3.24×10^{-4}**	**5.62×10^{-26}**	0.404	0.0125	**4.85×10^{-15}**
Shrub		0.374	**1.02×10^{-7}**		.0108	**2.35×10^{-24}**		**7.75×10^{-5}**	**2.17×10^{-26}**
Forb			**0.00217**			**3.63×10^{-9}**			**3.10×10^{-9}**

Dataset S1 (separate file). Age-depth tie-points and updated splice used to create the age models for ODP 659 and DSDP 368.

Dataset S2 (separate file). Plant-wax concentration measurements from all replicates, C_{16} – C_{36}.

Dataset S3 (separate file). Plant-wax sample data including sample depth and location in core, inferred age, mean concentrations, carbon isotope ratios, derived metrics, and error estimates

SI References

Ali, H.A.M., Mayes, R.W., Hector, B.L., Verma, A.K., ØRskov, E.R., 2005. The possible use of *n*-alkanes, long-chain fatty alcohols and long-chain fatty acids as markers in studies of the botanical composition of the diet of free-ranging herbivores. The Journal of Agricultural Science 143(1), 85-95. doi.org/10.1017/S0021859605004958.

Andrae, J.W., McInerney, F.A., Polissar, P.J., Sniderman, J.M.K., Howard, S., Hall, P.A., Phelps, S.R., 2018. Initial expansion of C_4 vegetation in Australia during the late Pliocene. Geophysical Research Letters 45(10), 4831-4840. doi.org/https://doi.org/10.1029/2018GL077833.

Arakaki, M., Christin, P.-A., Nyffeler, R., Lendel, A., Eggli, U., Ogburn, R.M., Spriggs, E., Moore, M.J., Edwards, E.J., 2011. Contemporaneous and recent radiations of the world's major succulent plant lineages. Proceedings of the National Academy of Sciences 108(20), 8379-8384. doi.org/10.1073/pnas.1100628108.

Backman, J., Raffi, I., Rio, D., Fornaciari, E., Pälike, H., 2012. Biozonation and biochronology of Miocene through Pleistocene calcareous nannofossils from low and middle latitudes. Newsletters on Stratigraphy 45, 221-244. doi.org/10.1127/0078-0421/2014/0042.

Badewien, T., Vogts, A., Dupont, L., Rullkötter, J., 2015. Influence of Late Pleistocene and Holocene climate on vegetation distributions in southwest Africa elucidated from sedimentary *n*-alkanes – Differences between 12°S and 20°S. Quaternary Science Reviews 125, 160-171. doi.org/https://doi.org/10.1016/j.quascirev.2015.08.004.

Bezabih, M., Pellikaan, W.F., Tolera, A., Hendriks, W.H., 2011. Evaluation of *n*-alkanes and their carbon isotope enrichments ($\delta^{13}C$) as diet composition markers. Animal 5(1), 57-66. doi.org/https://doi.org/10.1017/S1751731110001515.

Bonnefille, R., 2010. Cenozoic vegetation, climate changes and hominid evolution in tropical Africa. Global and Planetary Change 72(4), 390-411. doi.org/https://doi.org/10.1016/j.gloplacha.2010.01.015.

Boyle, B., Hopkins, N., Lu, Z., Raygoza Garay, J.A., Mozzherin, D., Rees, T., Matasci, N., Narro, M.L., Piel, W.H., McKay, S.J., Lowry, S., Freeland, C., Peet, R.K., Enquist, B.J., 2013. The taxonomic name resolution service: an online tool for automated standardization of plant names. BMC Bioinformatics 14(1), 16. doi.org/10.1186/1471-2105-14-16.

Bray, E.E., Evans, E.D., 1961. Distribution of *n*-paraffins as a clue to recognition of source beds. Geochimica et Cosmochimica Acta 22(1), 2-15. doi.org/https://doi.org/10.1016/0016-7037(61)90069-2.

Bukry, D., 1978. Cenozoic coccolith and silicoflagellate stratigraphy, offshore Northwest Africa, Deep Sea Drilling Project, Leg 41, in: Gardner, J., Herring, J. (Eds.), Initial Reports of the Deep Sea Drilling Project, Vol. 41. Texas A & M University, Ocean Drilling Program, College Station, TX, United States, pp. 689-707. doi.org/doi: 10.2973/dsdp.proc.41.115.1978.

Čepek, P., Johnson, D., Krasheninnikov, V., Pflaumann, U., Herring, J., 1978. Synthesis of the Leg 41 biostratigraphy and paleontology, Deep Sea Drilling Project, in: Gardner, J., Herring, J. (Eds.), Initial Reports of the Deep Sea Drilling Project, Vol. 41. Texas A & M University, Ocean Drilling Program, College Station, TX, United States, pp. 1181-1198. doi.org/doi: 10.2973/dsdp.proc.41.149.1978.

Chevalier, N., Savoye, N., Dubois, S., Lama, M.L., David, V., Lecroart, P., le Ménach, K., Budzinski, H., 2015. Precise indices based on *n*-alkane distribution for quantifying sources of sedimentary organic matter in coastal systems. Organic Geochemistry 88, 69-77. doi.org/https://doi.org/10.1016/j.orggeochem.2015.07.006.

Cornwell, W.K., Wright, I.J., Turner, J., Maire, V., Barbour, M.M., Cernusak, L.A., Dawson, T., Ellsworth, D., Farquhar, G.D., Griffiths, H., Keitel, C., Knohl, A., Reich, P.B., Williams, D.G., Bhaskar, R., Cornelissen, J.H.C., Richards, A., Schmidt, S., Valladares, F., Körner, C., Schulze, E.-D., Buchmann, N., Santiago, L.S., 2018. Climate and soils together regulate photosynthetic carbon isotope discrimination within C_3 plants worldwide. Global Ecology and Biogeography 27(9), 1056-1067. doi.org/https://doi.org/10.1111/geb.12764.

Crocker, A.J., Naafs, B.D.A., Westerhold, T., James, R.H., Cooper, M.J., Röhl, U., Pancost, R.D., Xuan, C., Osborne, C.P., Beerling, D.J., Wilson, P.A., 2022. Astronomically controlled aridity in the Sahara since at least 11 million years ago. Nature Geoscience 15(8), 671-676. doi.org/10.1038/s41561-022-00990-7.

Diefendorf, A.F., Freimuth, E.J., 2017. Extracting the most from terrestrial plant-derived *n*-alkyl lipids and their carbon isotopes from the sedimentary record: A review. Organic Geochemistry 103, 1-21. doi.org/https://doi.org/10.1016/j.orggeochem.2016.10.016.

Dodd, R.S., Fromard, F., Rafii, Z.A., Blasco, F., 1995. Biodiversity among West African Rhizophora: foliar wax chemistry. Biochemical Systematics and Ecology 23(7), 859-868. doi.org/https://doi.org/10.1016/0305-1978(95)00083-6.

Dupont, L.M., Rommerskirchen, F., Mollenhauer, G., Schefuß, E., 2013. Miocene to Pliocene changes in South African hydrology and vegetation in relation to the expansion of C_4 plants. Earth and Planetary Science Letters 375, 408-417. doi.org/https://doi.org/10.1016/j.epsl.2013.06.005.

Eglinton, G., Hamilton, R.J., 1967. Leaf epicuticular waxes. Science 156(3780), 1322. doi.org/10.1126/science.156.3780.1322.

Feakins, S.J., Levin, N.E., Liddy, H.M., Sieracki, A., Eglinton, T.I., Bonnefille, R., 2013. Northeast African vegetation change over 12 m.y. Geology 41(3), 295-298. doi.org/10.1130/g33845.1.

Feakins, S.J., Liddy, H.M., Tauxe, L., Galy, V., Feng, X., Tierney, J.E., Miao, Y., Warny, S., 2020. Miocene C_4 grassland expansion as recorded by the Indus Fan. Paleoceanography and Paleoclimatology 35(6), e2020PA003856. doi.org/https://doi.org/10.1029/2020PA003856.

Fox, D.L., Koch, P.L., 2004. Carbon and oxygen isotopic variability in Neogene paleosol carbonates: constraints on the evolution of the C_4-grasslands of the Great Plains, USA. Palaeogeography, Palaeoclimatology, Palaeoecology 207(3), 305-329. doi.org/https://doi.org/10.1016/j.palaeo.2003.09.030.

Garcin, Y., Schefuß, E., Schwab, V.F., Garreta, V., Gleixner, G., Vincens, A., Todou, G., Séné, O., Onana, J.-M., Achoundong, G., Sachse, D., 2014. Reconstructing C_3 and C_4 vegetation cover using *n*-alkane carbon isotope ratios in recent lake sediments from Cameroon, Western Central Africa. Geochimica et Cosmochimica Acta 142, 482-500. doi.org/https://doi.org/10.1016/j.gca.2014.07.004.

Garcin, Y., Schwab, V.F., Gleixner, G., Kahmen, A., Todou, G., Séné, O., Onana, J.-M., Achoundong, G., Sachse, D., 2012. Hydrogen isotope ratios of lacustrine sedimentary *n*-alkanes as proxies of tropical African hydrology: Insights from a calibration transect across Cameroon. Geochimica et Cosmochimica Acta 79, 106-126. doi.org/https://doi.org/10.1016/j.gca.2011.11.039.

Gensel, J., Humphries, M.S., Zabel, M., Sebag, D., Hahn, A., Schefuß, E., 2022. Origin, transport, and retention of fluvial sedimentary organic matter in South Africa's largest freshwater wetland, Mkhuze Wetland System. Biogeosciences 19(11), 2881-2902. doi.org/10.5194/bg-19-2881-2022.

Griepentrog, M., De Wispelaere, L., Bauters, M., Bodé, S., Hemp, A., Verschuren, D., Boeckx, P., 2019. Influence of plant growth form, habitat and season on leaf-wax *n*-alkane hydrogen-isotopic signatures in equatorial East Africa. Geochimica et Cosmochimica Acta 263, 122-139. doi.org/https://doi.org/10.1016/j.gca.2019.08.004.

Häggi, C., Bertassoli Jr., D.J., Akabane, T.K., So, R.T., Sawakuchi, A.O., Chiessi, C.M., Mendes, V.R., Jaramillo, C.A., Feakins, S.J., 2024. Using multi-homolog plant-wax carbon isotope compositions to reconstruct tropical vegetation types. Journal of Geophysical Research: Biogeosciences 129(4), e2023JG007946. doi.org/https://doi.org/10.1029/2023JG007946.

Hoetzel, S., Dupont, L., Schefuß, E., Rommerskirchen, F., Wefer, G., 2013. The role of fire in Miocene to Pliocene C_4 grassland and ecosystem evolution. Nature Geoscience 6(12), 1027-1030. doi.org/10.1038/ngeo1984.

Hoetzel, S., Dupont, L.M., Wefer, G., 2015. Miocene–Pliocene vegetation change in south-western Africa (ODP Site 1081, offshore Namibia). Palaeogeography, Palaeoclimatology, Palaeoecology 423, 102-108. doi.org/10.1016/j.palaeo.2015.02.002.

Hooghiemstra, H., Lézine, A.-M., Leroy, S.A.G., Dupont, L., Marret, F., 2006. Late Quaternary palynology in marine sediments: A synthesis of the understanding of pollen distribution patterns in the NW African setting. Quaternary International 148(1), 29-44. doi.org/https://doi.org/10.1016/j.quaint.2005.11.005.

Huang, Y., Clemens, S.C., Liu, W., Wang, Y., Prell, W.L., 2007. Large-scale hydrological change drove the late Miocene C_4 plant expansion in the Himalayan foreland and Arabian Peninsula. Geology 35(6), 531-534. doi.org/10.1130/G23666A.1.

Huang, Y., Dupont, L., Sarnthein, M., Hayes, J.M., Eglinton, G., 2000. Mapping of C_4 plant input from North West Africa into North East Atlantic sediments. Geochimica et Cosmochimica Acta 64(20), 3505-3513. doi.org/https://doi.org/10.1016/S0016-7037(00)00445-2.

Jewell, A.M., Drake, N., Crocker, A.J., Bakker, N.L., Kunkelova, T., Bristow, C.S., Cooper, M.J., Milton, J.A., Breeze, P.S., Wilson, P.A., 2021. Three North African dust source areas and their geochemical fingerprint. Earth and Planetary Science Letters 554, 116645. doi.org/https://doi.org/10.1016/j.epsl.2020.116645.

Johnson, D., 1978. Cenozoic Radiolaria from the eastern tropical Atlantic, DSDP, Leg 41, in: Gardner, J., Herring, J. (Eds.), Initial Reports of the Deep Sea Drilling Project, Vol. 41. Texas A & M University, Ocean Drilling Program, College Station, TX, United States, pp. 763-789. doi.org/doi: 10.2973/dsdp.proc.41.118.1978.

Kattge, J., Bönisch, G., Díaz, S., Lavorel, S., Prentice, I.C., Leadley, P., Tautenhahn, S., Werner, G.D.A., Aakala, T., Abedi, M., Acosta, A.T.R., Adamidis, G.C., Adamson, K., Aiba, M., Albert, C.H., Alcántara, J.M., Alcázar C, C., Aleixo, I., Ali, H., Amiaud, B., Ammer, C., Amoroso, M.M., Anand, M., Anderson, C., Anten, N., Antos, J., Apgaua, D.M.G., Ashman, T.-L., Asmara, D.H., Asner, G.P., Aspinwall, M., Atkin, O., Aubin, I., Baastrup-Spohr, L., Bahalkeh, K., Bahn, M., Baker, T., Baker, W.J., Bakker, J.P., Baldocchi, D., Baltzer, J., Banerjee, A., Baranger, A., Barlow, J., Barneche, D.R., Baruch, Z., Bastianelli, D., Battles, J., Bauerle, W., Bauters, M., Bazzato, E., Beckmann, M., Beeckman, H., Beierkuhnlein, C., Bekker, R., Belfry, G., Belluau, M., Beloiu, M., Benavides, R., Benomar, L., Berdugo-Lattke, M.L., Berenguer, E., Bergamin, R., Bergmann, J., Bergmann Carlucci, M., Berner, L., Bernhardt-Römermann, M., Bigler, C., Bjorkman, A.D., Blackman, C., Blanco, C., Blonder, B., Blumenthal, D., Bocanegra-González, K.T., Boeckx, P., Bohlman, S., Böhning-Gaese, K., Boisvert-Marsh, L., Bond, W., Bond-Lamberty, B., Boom, A., Boonman, C.C.F., Bordin, K., Boughton, E.H., Boukili, V., Bowman, D.M.J.S., Bravo, S., Brendel, M.R., Broadley, M.R., Brown, K.A., Bruelheide, H., Brumnich, F., Bruun, H.H., Bruy, D., Buchanan, S.W., Bucher, S.F., Buchmann, N., Buitenwerf, R., Bunker, D.E., Bürger, J., Burrascano, S., Burslem, D.F.R.P., Butterfield, B.J., Byun, C., Marques, M., Scalon, M.C., Caccianiga, M., Cadotte, M., Cailleret, M., Camac, J., Camarero, J.J., Campany, C., Campetella, G., Campos, J.A., Cano-Arboleda, L., Canullo, R., Carbognani, M., Carvalho, F., Casanoves, F., Castagneyrol, B., Catford, J.A., Cavender-Bares, J., Cerabolini, B.E.L., Cervellini, M., Chacón-Madrigal, E., Chapin, K., Chapin, F.S., Chelli, S., Chen, S.-C., Chen, A., Cherubini, P., Chianucci, F., Choat, B., Chung, K.-S., Chytrý, M., Ciccarelli, D., Coll, L., Collins, C.G., Conti, L., Coomes, D., Cornelissen, J.H.C., Cornwell, W.K., Corona, P., Coyea, M., Craine, J., Craven, D., Cromsigt, J.P.G.M., Csecserits, A., Cufar, K., Cuntz, M., da Silva, A.C., Dahlin, K.M., Dainese, M., Dalke, I., Dalle Fratte, M., Dang-Le, A.T., Danihelka, J.,

Dannoura, M., Dawson, S., de Beer, A.J., De Frutos, A., De Long, J.R., Dechant, B., Delagrange, S., Delpierre, N., Derroire, G., Dias, A.S., Diaz-Toribio, M.H., Dimitrakopoulos, P.G., Dobrowolski, M., Doktor, D., Dřevojan, P., Dong, N., Dransfield, J., Dressler, S., Duarte, L., Ducouret, E., Dullinger, S., Durka, W., Duursma, R., Dymova, O., E-Vojtkó, A., Eckstein, R.L., Ejtehadi, H., Elser, J., Emilio, T., Engemann, K., Erfanian, M.B., Erfmeier, A., Esquivel-Muelbert, A., Esser, G., Estiarte, M., Domingues, T.F., Fagan, W.F., Fagúndez, J., Falster, D.S., Fan, Y., Fang, J., Farris, E., Fazlioglu, F., Feng, Y., Fernandez-Mendez, F., Ferrara, C., Ferreira, J., Fidelis, A., Finegan, B., Firn, J., Flowers, T.J., Flynn, D.F.B., Fontana, V., Forey, E., Forgiarini, C., François, L., Frangipani, M., Frank, D., Frenette-Dussault, C., Freschet, G.T., Fry, E.L., Fyllas, N.M., Mazzochini, G.G., Gachet, S., Gallagher, R., Ganade, G., Ganga, F., García-Palacios, P., Gargaglione, V., Garnier, E., Garrido, J.L., de Gasper, A.L., Gea-Izquierdo, G., Gibson, D., Gillison, A.N., Giroldo, A., Glasenhardt, M.-C., Gleason, S., Gliesch, M., Goldberg, E., Göldel, B., Gonzalez-Akre, E., Gonzalez-Andujar, J.L., González-Melo, A., González-Robles, A., Graae, B.J., Granda, E., Graves, S., Green, W.A., Gregor, T., Gross, N., Guerin, G.R., Günther, A., Gutiérrez, A.G., Haddock, L., Haines, A., Hall, J., Hambuckers, A., Han, W., Harrison, S.P., Hattingh, W., Hawes, J.E., He, T., He, P., Heberling, J.M., Helm, A., Hempel, S., Hentschel, J., Hérault, B., Hereş, A.-M., Herz, K., Heuertz, M., Hickler, T., Hietz, P., Higuchi, P., Hipp, A.L., Hirons, A., Hock, M., Hogan, J.A., Holl, K., Honnay, O., Hornstein, D., Hou, E., Hough-Snee, N., Hovstad, K.A., Ichie, T., Igić, B., Illa, E., Isaac, M., Ishihara, M., Ivanov, L., Ivanova, L., Iversen, C.M., Izquierdo, J., Jackson, R.B., Jackson, B., Jactel, H., Jagodzinski, A.M., Jandt, U., Jansen, S., Jenkins, T., Jentsch, A., Jespersen, J.R.P., Jiang, G.-F., Johansen, J.L., Johnson, D., Jokela, E.J., Joly, C.A., Jordan, G.J., Joseph, G.S., Junaedi, D., Junker, R.R., Justes, E., Kabzems, R., Kane, J., Kaplan, Z., Kattenborn, T., Kavelenova, L., Kearsley, E., Kempel, A., Kenzo, T., Kerkhoff, A., Khalil, M.I., Kinlock, N.L., Kissling, W.D., Kitajima, K., Kitzberger, T., Kjøller, R., Klein, T., Kleyer, M., Klimešová, J., Klipel, J., Kloeppel, B., Klotz, S., Knops, J.M.H., Kohyama, T., Koike, F., Kollmann, J., Komac, B., Komatsu, K., König, C., Kraft, N.J.B., Kramer, K., Kreft, H., Kühn, I., Kumarathunge, D., Kuppler, J., Kurokawa, H., Kurosawa, Y., Kuyah, S., Laclau, J.-P., Lafleur, B., Lallai, E., Lamb, E., Lamprecht, A., Larkin, D.J., Laughlin, D., Le Bagousse-Pinguet, Y., le Maire, G., le Roux, P.C., le Roux, E., Lee, T., Lens, F., Lewis, S.L., Lhotsky, B., Li, Y., Li, X., Lichstein, J.W., Liebergesell, M., Lim, J.Y., Lin, Y.-S., Linares, J.C., Liu, C., Liu, D., Liu, U., Livingstone, S., Llusià, J., Lohbeck, M., López-García, Á., Lopez-Gonzalez, G., Lososová, Z., Louault, F., Lukács, B.A., Lukeš, P., Luo, Y., Lussu, M., Ma, S., Maciel Rabelo Pereira, C., Mack, M., Maire, V., Mäkelä, A., Mäkinen, H., Malhado, A.C.M., Mallik, A., Manning, P., Manzoni, S., Marchetti, Z., Marchino, L., Marcilio-Silva, V., Marcon, E., Marignani, M., Markesteijn, L., Martin, A., Martínez-Garza, C., Martínez-Vilalta, J., Mašková, T., Mason, K., Mason, N., Massad, T.J., Masse, J., Mayrose, I., McCarthy, J., McCormack, M.L., McCulloh, K., McFadden, I.R., McGill, B.J., McPartland, M.Y., Medeiros, J.S., Medlyn, B., Meerts, P., Mehrabi, Z., Meir, P., Melo, F.P.L., Mencuccini, M., Meredieu, C., Messier, J., Mészáros, I., Metsaranta, J., Michaletz, S.T., Michelaki, C., Migalina, S., Milla, R., Miller, J.E.D., Minden, V., Ming, R., Mokany, K., Moles, A.T., Molnár V, A., Molofsky, J., Molz, M., Montgomery, R.A., Monty, A., Moravcová, L., Moreno-Martínez, A., Moretti, M., Mori, A.S., Mori, S., Morris, D., Morrison, J., Mucina, L., Mueller, S., Muir, C.D., Müller, S.C., Munoz, F., Myers-Smith, I.H., Myster, R.W., Nagano, M., Naidu, S., Narayanan, A., Natesan, B., Negoita, L., Nelson, A.S., Neuschulz, E.L., Ni, J., Niedrist, G., Nieto, J., Niinemets, Ü., Nolan, R., Nottebrock, H., Nouvellon, Y., Novakovskiy, A., The Nutrient, N., Nystuen, K.O., O'Grady, A., O'Hara, K., O'Reilly-Nugent, A., Oakley, S., Oberhuber, W., Ohtsuka, T., Oliveira, R.,

Öllerer, K., Olson, M.E., Onipchenko, V., Onoda, Y., Onstein, R.E., Ordonez, J.C., Osada, N., Ostonen, I., Ottaviani, G., Otto, S., Overbeck, G.E., Ozinga, W.A., Pahl, A.T., Paine, C.E.T., Pakeman, R.J., Papageorgiou, A.C., Parfionova, E., Pärtel, M., Patacca, M., Paula, S., Paule, J., Pauli, H., Pausas, J.G., Peco, B., Penuelas, J., Perea, A., Peri, P.L., Petisco-Souza, A.C., Petraglia, A., Petritan, A.M., Phillips, O.L., Pierce, S., Pillar, V.D., Pisek, J., Pomogaybin, A., Poorter, H., Portsmuth, A., Poschlod, P., Potvin, C., Pounds, D., Powell, A.S., Power, S.A., Prinzing, A., Puglielli, G., Pyšek, P., Raevel, V., Rammig, A., Ransijn, J., Ray, C.A., Reich, P.B., Reichstein, M., Reid, D.E.B., Réjou-Méchain, M., de Dios, V.R., Ribeiro, S., Richardson, S., Riibak, K., Rillig, M.C., Riviera, F., Robert, E.M.R., Roberts, S., Robroek, B., Roddy, A., Rodrigues, A.V., Rogers, A., Rollinson, E., Rolo, V., Römermann, C., Ronzhina, D., Roscher, C., Rosell, J.A., Rosenfield, M.F., Rossi, C., Roy, D.B., Royer-Tardif, S., Rüger, N., Ruiz-Peinado, R., Rumpf, S.B., Rusch, G.M., Ryo, M., Sack, L., Saldaña, A., Salgado-Negret, B., Salguero-Gomez, R., Santa-Regina, I., Santacruz-García, A.C., Santos, J., Sardans, J., Schamp, B., Scherer-Lorenzen, M., Schleuning, M., Schmid, B., Schmidt, M., Schmitt, S., Schneider, J.V., Schowanek, S.D., Schrader, J., Schrodt, F., Schuldt, B., Schurr, F., Selaya Garvizu, G., Semchenko, M., Seymour, C., Sfair, J.C., Sharpe, J.M., Sheppard, C.S., Sheremetiev, S., Shiodera, S., Shipley, B., Shovon, T.A., Siebenkäs, A., Sierra, C., Silva, V., Silva, M., Sitzia, T., Sjöman, H., Slot, M., Smith, N.G., Sodhi, D., Soltis, P., Soltis, D., Somers, B., Sonnier, G., Sørensen, M.V., Sosinski Jr, E.E., Soudzilovskaia, N.A., Souza, A.F., Spasojevic, M., Sperandii, M.G., Stan, A.B., Stegen, J., Steinbauer, K., Stephan, J.G., Sterck, F., Stojanovic, D.B., Strydom, T., Suarez, M.L., Svenning, J.-C., Svitková, I., Svitok, M., Svoboda, M., Swaine, E., Swenson, N., Tabarelli, M., Takagi, K., Tappeiner, U., Tarifa, R., Tauugourdeau, S., Tavsanoglu, C., te Beest, M., Tedersoo, L., Thiffault, N., Thom, D., Thomas, E., Thompson, K., Thornton, P.E., Thuiller, W., Tichý, L., Tissue, D., Tjoelker, M.G., Tng, D.Y.P., Tobias, J., Török, P., Tarin, T., Torres-Ruiz, J.M., Tóthmérész, B., Treurnicht, M., Trivellone, V., Trolliet, F., Trotsiuk, V., Tsakalos, J.L., Tsiripidis, I., Tysklind, N., Umehara, T., Usoltsev, V., Vadeboncoeur, M., Vaezi, J., Valladares, F., Vamosi, J., van Bodegom, P.M., van Breugel, M., Van Cleemput, E., van de Weg, M., van der Merwe, S., van der Plas, F., van der Sande, M.T., van Kleunen, M., Van Meerbeek, K., Vanderwel, M., Vanselow, K.A., Vårhammar, A., Varone, L., Vasquez Valderrama, M.Y., Vassilev, K., Vellend, M., Veneklaas, E.J., Verbeeck, H., Verheyen, K., Vibrans, A., Vieira, I., Villacís, J., Violle, C., Vivek, P., Wagner, K., Waldram, M., Waldron, A., Walker, A.P., Waller, M., Walther, G., Wang, H., Wang, F., Wang, W., Watkins, H., Watkins, J., Weber, U., Weedon, J.T., Wei, L., Weigelt, P., Weiher, E., Wells, A.W., Wellstein, C., Wenk, E., Westoby, M., Westwood, A., White, P.J., Whitten, M., Williams, M., Winkler, D.E., Winter, K., Womack, C., Wright, I.J., Wright, S.J., Wright, J., Pinho, B.X., Ximenes, F., Yamada, T., Yamaji, K., Yanai, R., Yankov, N., Yguel, B., Zanini, K.J., Zanne, A.E., Zelený, D., Zhao, Y.-P., Zheng, J., Zheng, J., Ziemińska, K., Zirbel, C.R., Zizka, G., Zo-Bi, I.C., Zotz, G., Wirth, C., 2020. TRY plant trait database – enhanced coverage and open access. Global Change Biology 26(1), 119-188. doi.org/https://doi.org/10.1111/gcb.14904.

Kherraze, M.E., Belhamra, M., Grigore, M.-N., 2018. Aspects of ecological anatomy of Traganum nudatum Del. (Amaranthaceae) from the Northeast of the Algerian Sahara. Acta Biologica Szegediensis 62(1), 25-36. doi.org/10.14232/abs.2018.1.25-36.

Kohn, M.J., 2010. Carbon isotope compositions of terrestrial C_3 plants as indicators of (paleo)ecology and (paleo)climate. Proceedings of the National Academy of Sciences 107(46), 19691-19695. doi.org/10.1073/pnas.1004933107.

Kristen, I., Wilkes, H., Vieth, A., Zink, K.G., Plessen, B., Thorpe, J., Partridge, T.C., Oberhänsli, H., 2010. Biomarker and stable carbon isotope analyses of sedimentary organic matter from Lake Tswaing: evidence for deglacial wetness and early Holocene drought from South Africa. Journal of Paleolimnology 44(1), 143-160. doi.org/10.1007/s10933-009-9393-9.

Lancelot, Y., Seibold, E., Čepek, P., Dean, W.E., Eremeev, V., Gardner, J., Jansa, L.F., Johnson, D., Krasheninnikov, V., Pflaumann, U., Rankin, J.G., Trabant, P., Bukry, D., 1978. Site 368; Cape Verde Rise, in: Gardner, J., Herring, J. (Eds.), Initial Reports of the Deep Sea Drilling Project, Vol. 41. Texas A & M University, Ocean Drilling Program, College Station, TX, United States, pp. 233-326. doi.org/https://doi.org/10.2973/dsdp.proc.41.1978.

Laskar, J., Robutel, P., Joutel, F., Gastineau, M., Correia, A.C.M., Levrard, B., 2004. A long-term numerical solution for the insolation quantities of the Earth. A&A 428(1), 261-285.

Lathuilière, C., Echevin, V., Lévy, M., 2008. Seasonal and intraseasonal surface chlorophyll-a variability along the northwest African coast. Journal of Geophysical Research: Oceans 113, C05007. doi.org/https://doi.org/10.1029/2007JC004433.

Latorre, C., Quade, J., McIntosh, W.C., 1997. The expansion of C_4 grasses and global change in the late Miocene: Stable isotope evidence from the Americas. Earth and Planetary Science Letters 146(1), 83-96. doi.org/https://doi.org/10.1016/S0012-821X(96)00231-2.

Leroy, S., Dupont, L., 1994. Development of vegetation and continental aridity in northwestern Africa during the Late Pliocene: the pollen record of ODP site 658. Palaeogeography, Palaeoclimatology, Palaeoecology 109(2), 295-316. doi.org/https://doi.org/10.1016/0031-0182(94)90181-3.

Leroy, S.A.G., Dupont, L.M., 1997. Marine palynology of the ODP site 658 (N-W Africa) and its contribution to the stratigraphy of Late Pliocene. Geobios 30(3), 351-359. doi.org/https://doi.org/10.1016/S0016-6995(97)80194-5.

Liebezeit, G., Wöstmann, R., 2009. *n*-Alkanes as indicators of natural and anthropogenic organic matter sources in the Siak River and its estuary, E Sumatra, Indonesia. Bulletin of Environmental Contamination and Toxicology 83(3), 403-409. doi.org/10.1007/s00128-009-9734-4.

Lisiecki, L.E., Raymo, M.E., 2005. A Pliocene-Pleistocene stack of 57 globally distributed benthic $\delta^{18}O$ records. Paleoceanography and Paleoclimatology 20(1), PA1003. doi.org/https://doi.org/10.1029/2004PA001071.

Liu, J., Zhao, J., He, D., Huang, X., Jiang, C., Yan, H., Lin, G., An, Z., 2022. Effects of plant types on terrestrial leaf wax long-chain *n*-alkane biomarkers: Implications and paleoapplications. Earth-Science Reviews 235, 104248. doi.org/https://doi.org/10.1016/j.earscirev.2022.104248.

Magill, C.R., Eglinton, G., Eglinton, T.I., 2019. Isotopic variance among plant lipid homologues correlates with biodiversity patterns of their source communities. PLOS ONE 14(2), e0212211. doi.org/10.1371/journal.pone.0212211.

Marzi, R., Torkelson, B.E., Olson, R.K., 1993. A revised carbon preference index. Organic Geochemistry 20(8), 1303-1306. doi.org/https://doi.org/10.1016/0146-6380(93)90016-5.

Merritt, D.A., Freeman, K.H., Ricci, M.P., Studley, S.A., Hayes, J.M., 1995. Performance and optimization of a combustion interface for isotope ratio monitoring gas chromatography/mass spectrometry. Analytical Chemistry 67(14), 2461-2473. doi.org/10.1021/ac00110a022.

Nicholson, S.E., 2013. The West African Sahel: a review of recent studies on the rainfall regime and its interannual variability. International Scholarly Research Notices, 453521. doi.org/10.1155/2013/453521.

Niedermeyer, E.M., Forrest, M., Beckmann, B., Sessions, A.L., Mulch, A., Schefuß, E., 2016. The stable hydrogen isotopic composition of sedimentary plant waxes as quantitative proxy for rainfall in the West African Sahel. Geochimica et Cosmochimica Acta 184, 55-70. doi.org/https://doi.org/10.1016/j.gca.2016.03.034.

Nigrini, C.A., Sanfilippo, A., Moore Jr., T.C., 2006. Cenozoic radiolarian biostratigraphy: a magnetobiostratigraphic chronology of Cenozoic sequences from ODP Sites 1218, 1219, and 1220, equatorial Pacific, in: Wilson, P.A., Lyle, M., Firth, J.V. (Eds.), Proc. ODP, Sci. Results, 199, Ocean Drilling Program. College Station, TX, pp. 1-76. doi.org/https://doi.org/10.2973/odp.proc.sr.199.225.2006.

O'Mara, N.A., de Menocal, P.B., Uno, K.T., Tierney, J.E., Winckler, G., Polissar, P.J., 2025. Capturing vegetation gradients along the West African margin using terrestrial plant biomarkers in marine sediments. Geochemistry, Geophysics, Geosystems 26(7), e2025GC012274. doi.org/https://doi.org/10.1029/2025GC012274.

O'Mara, N.A., Skonieczny, C., McGee, D., Winckler, G., Bory, A.J.M., Bradtmiller, L.I., Malaizé, B., Polissar, P.J., 2022. Pleistocene drivers of Northwest African hydroclimate and vegetation. Nature Communications 13(1), 3552. doi.org/10.1038/s41467-022-31120-x.

O'Mara, N.A., Skonieczny, C., McGee, D., Winckler, G., Bory, A.J.M., Bradtmiller, L.I., Malaizé, B., Polissar, P.J., 2024. Constraining Plio-Pleistocene shifts in Northwest African hydroclimate, ecosystem distributions, and marine productivity: new paleo-records across the mid-Pleistocene transition. Paleoceanography and Paleoclimatology 39(6), e2023PA004777. doi.org/https://doi.org/10.1029/2023PA004777.

Passey, B.H., Ayliffe, L.K., Kaakinen, A., Zhang, Z., Eronen, J.T., Zhu, Y., Zhou, L., Cerling, T.E., Fortelius, M., 2009. Strengthened East Asian summer monsoons during a period of high-latitude warmth? Isotopic evidence from Mio-Pliocene fossil mammals and soil carbonates from northern China. Earth and Planetary Science Letters 277(3), 443-452. doi.org/https://doi.org/10.1016/j.epsl.2008.11.008.

Pedregosa, F., Varoquaux, G., Gramfort, A., Michel, V., Thirion, B., Grisel, O., Blondel, M., Prettenhofer, P., Weiss, R., Dubourg, V., Vanderplas, J., Passos, A., Cournapeau, D., Brucher, M., Perrot, M., Duchesnay, É., 2011. Scikit-learn: machine learning in Python. The Journal of Machine Learning Research 12, 2825-2830. doi.org/https://doi.org/10.5555/1953048.2078195.

Polissar, P.J., D'Andrea, W.J., 2014. Uncertainty in paleohydrologic reconstructions from molecular δD values. Geochimica et Cosmochimica Acta 129, 146-156. doi.org/https://doi.org/10.1016/j.gca.2013.12.021.

Polissar, P.J., Rose, C., Uno, K.T., Phelps, S.R., deMenocal, P., 2019. Synchronous rise of African C_4 ecosystems 10 million years ago in the absence of aridification. Nature Geoscience 12(8), 657-660. doi.org/10.1038/s41561-019-0399-2.

Polissar, P.J., Uno, K.T., Phelps, S.R., Karp, A.T., Freeman, K.H., Pensky, J.L., 2021. Hydrologic changes drove the late Miocene expansion of C_4 grasslands on the northern Indian subcontinent. Paleoceanography and Paleoclimatology 36(4), e2020PA004108. doi.org/https://doi.org/10.1029/2020PA004108.

Prospero, J.M., Delany, A.C., Delany, A.C., Carlson, T.N., 2021. The discovery of African dust transport to the Western Hemisphere and the Saharan Air Layer: a history. Bulletin of the American Meteorological Society 102(6), E1239-E1260. doi.org/https://doi.org/10.1175/BAMS-D-19-0309.1.

Raffi, I., Agnini, C., Backman, J., Catanzariti, R., 2016. A Cenozoic calcareous nannofossil biozonation from low and middle latitudes: A synthesis. Journal of Nannoplankton Research 36, 121-132. doi.org/10.58998/jnr2206.

Rommerskirchen, F., Eglinton, G., Dupont, L., Güntner, U., Wenzel, C., Rullkötter, J., 2003. A north to south transect of Holocene southeast Atlantic continental margin sediments: Relationship between aerosol transport and compound-specific $\delta^{13}C$ land plant biomarker and pollen records. Geochemistry, Geophysics, Geosystems 4(12). doi.org/https://doi.org/10.1029/2003GC000541.

Rommerskirchen, F., Plader, A., Eglinton, G., Chikaraishi, Y., Rullkötter, J., 2006. Chemotaxonomic significance of distribution and stable carbon isotopic composition of long-chain alkanes and alkan-1-ols in C_4 grass waxes. Organic Geochemistry 37(10), 1303-1332. doi.org/https://doi.org/10.1016/j.orggeochem.2005.12.013.

Ruddiman, W., Sarnthein, M., Baldauf, J., Backman, J., Bloemendal, J., Curry, W., Farrimond, P., Faugeres, J.C., Janacek, T., Katsura, Y., Manivit, H., Mazzullo, J., Mienert, J., Pokras, E., Raymo, M., Schultheiss, P., Stein, R., Tauxe, L., Valet, J.-P., Weaver, P., Yasuda, H., 1988. Site 659, Proceedings of the Ocean Drilling Program, Part A: Initial Reports, Vol. 108. Texas A & M University, Ocean Drilling Program, College Station, TX, United States, p. 221. doi.org/doi:10.2973/odp.proc.ir.108.1988.

Sage, R.F., Sage, T.L., Pearcy, R.W., Borsch, T., 2007. The taxonomic distribution of C_4 photosynthesis in Amaranthaceae sensu stricto. American Journal of Botany 94(12), 1992-2003. doi.org/https://doi.org/10.3732/ajb.94.12.1992.

Sarnthein, M., Thiede, J., Pflaumann, U., Erlenkeuser, H., Fütterer, D., Koopmann, B., Lange, H., Seibold, E., 1982. Atmospheric and oceanic circulation patterns off Northwest Africa during the past 25 million years, in: von Rad, U., Hinz, K., Sarnthein, M., Seibold, E. (Eds.), Geology of

the Northwest African Continental Margin. Springer, Berlin, Heidelberg, Berlin, Heidelberg, pp. 545-604.

Schefuß, E., Ratmeyer, V., Stuut, J.-B.W., Jansen, J.H.F., Sinninghe Damsté, J.S., 2003. Carbon isotope analyses of *n*-alkanes in dust from the lower atmosphere over the central eastern Atlantic. Geochimica et Cosmochimica Acta 67(10), 1757-1767. doi.org/https://doi.org/10.1016/S0016-7037(02)01414-X.

Schwab, V.F., Garcin, Y., Sachse, D., Todou, G., Séné, O., Onana, J.-M., Achoundong, G., Gleixner, G., 2015. Effect of aridity on $\delta^{13}C$ and δD values of C_3 plant- and C_4 graminoid-derived leaf wax lipids from soils along an environmental gradient in Cameroon (Western Central Africa). Organic Geochemistry 78, 99-109. doi.org/10.1016/j.orggeochem.2014.09.007.

Sonibare, M., Soladoye, M., Ekine-Ogunlana, Y., 2010. A chemotaxonomic approach to the alkane content of three species of Anthocleista Afzel. (Loganiaceae). African Journal of Biotechnology 6, 1516-1520.

Tiedemann, R., Sarnthein, M., Shackleton, N.J., 1994. Astronomic timescale for the Pliocene Atlantic $\delta^{18}O$ and dust flux records of Ocean Drilling Program Site 659. Paleoceanography 9(4), 619-638. doi.org/https://doi.org/10.1029/94PA00208.

Tipple, B.J., Meyers, S.R., Pagani, M., 2010. Carbon isotope ratio of Cenozoic CO_2: A comparative evaluation of available geochemical proxies. Paleoceanography 25(3), PA3202. doi.org/https://doi.org/10.1029/2009PA001851.

Tweedy, R.R., Strömberg, C., Reichgelt, T., Kinyanjui, R.N., Kimeu, M., Munyaka, V., Uno, K.T., 2025. African C_3 grass *n*-alkane distributions & plant chemotaxonomy. Organic Geochemistry 208, 104994.

Uno, K.T., Cerling, T.E., Harris, J.M., Kunimatsu, Y., Leakey, M.G., Nakatsukasa, M., Nakaya, H., 2011. Late Miocene to Pliocene carbon isotope record of differential diet change among East African herbivores. Proceedings of the National Academy of Sciences 108(16), 6509-6514. doi.org/10.1073/pnas.1018435108.

Uno, K.T., Polissar, P.J., Jackson, K.E., deMenocal, P.B., 2016. Neogene biomarker record of vegetation change in eastern Africa. Proceedings of the National Academy of Sciences 113(23), 6355-6363. doi.org/10.1073/pnas.1521267113.

Vallé, F., Dupont, L.M., Leroy, S.A.G., Schefuß, E., Wefer, G., 2014. Pliocene environmental change in West Africa and the onset of strong NE trade winds (ODP Sites 659 and 658). Palaeogeography, Palaeoclimatology, Palaeoecology 414, 403-414. doi.org/https://doi.org/10.1016/j.palaeo.2014.09.023.

Vogts, A., Moossen, H., Rommerskirchen, F., Rullkötter, J., 2009. Distribution patterns and stable carbon isotopic composition of alkanes and alkan-1-ols from plant waxes of African rain forest and savanna C_3 species. Organic Geochemistry 40(10), 1037-1054. doi.org/https://doi.org/10.1016/j.orggeochem.2009.07.011.

Vogts, A., Schefuß, E., Badewien, T., Rullkötter, J., 2012. *n*-Alkane parameters from a deep sea sediment transect off southwest Africa reflect continental vegetation and climate conditions. Organic Geochemistry 47, 109-119. doi.org/https://doi.org/10.1016/j.orggeochem.2012.03.011.

Wubben, E., Spiering, B.R., Veenstra, T., Bos, R., Wang, Z., van Dijk, J., Raffi, I., Witkowski, J., Hilgen, F.J., Peterse, F., Sangiorgi, F., Sluijs, A., 2024. Tropical warming and intensification of the West African monsoon during the Miocene Climatic Optimum. Paleoceanography and Paleoclimatology 39(5), e2023PA004767. doi.org/https://doi.org/10.1029/2023PA004767.

Young, J.R., 1998. Neogene, in: Brown, P.R. (Ed.) Calcareous Nannofossil Biostratigraphy. Chapman & Hall, London, UK, pp. 225-265.

Zhang, D., Beverly, E.J., Levin, N.E., Vidal, E., Matia, Y., Feakins, S.J., 2021. Carbon isotopic composition of plant waxes, bulk organics and carbonates from soils of the Serengeti grasslands. Geochimica et Cosmochimica Acta 311, 316-331.

Zhou, B., Bird, M., Zheng, H., Zhang, E., Wurster, C.M., Xie, L., Taylor, D., 2017. New sedimentary evidence reveals a unique history of C_4 biomass in continental East Asia since the early Miocene. Scientific Reports 7(1), 170. doi.org/10.1038/s41598-017-00285-7.
